# Delineating Pixantrone Maleate’s adroit activity against cervical cancer proteins through multitargeted docking-based MM\GBSA, QM-DFT and MD simulation

**DOI:** 10.1371/journal.pone.0295714

**Published:** 2023-12-15

**Authors:** Hassan Hussain Almasoudi, Mohammed H. Nahari, Abdulfattah Yahya M. Alhazmi, Saleh Hussain A. Almasabi, Fares Saeed H. Al-Mansour, Mohammed Ageeli Hakami

**Affiliations:** 1 Department of Clinical Laboratory Sciences, College of Applied Medical Sciences, Najran University, Najran, Kingdom of Saudi Arabia; 2 Department of Clinical Pharmacy, Umm Al-Qura University, Makkah, Kingdom of Saudi Arabia; 3 Department of Clinical Laboratory Sciences, College of Applied Medical Sciences, Al-Quwayiyah, Shaqra University, Riyadh, Kingdom of Saudi Arabia; Anadolu University: Anadolu Universitesi, TURKEY

## Abstract

Cervical cancer poses a substantial worldwide health challenge, especially in low- and middle-income nations, caused by high-risk types of human papillomavirus. It accounted for a significant percentage of cancer-related deaths among women, particularly in areas with limited healthcare resources, necessitating innovative therapeutic approaches, and single-targeted studies have produced significant results, with a considerable chance of developing resistance. Therefore, the multitargeted studies can work as a beacon of hope. This study is focused on performing the multitargeted molecular docking of FDA-approved drugs with the three crucial proteins TBK1, DNA polymerase epsilon, and integrin α-V β-8 of cervical cancer. The docking studies using multisampling algorithms HTVS, SP, and XP reveal Pixantrone Maleate (DB06193) as a multitargeted inhibitor with docking scores of -8.147, -8.206 and -7.31 Kcal/mol and pose filtration with MM\GBSA computations with scores -40.55, -33.67, and -37.64 Kcal/mol. We also have performed QM-based DFT and pharmacokinetics studies of the compound and compared it with the standard values, which results in the compound being entirely suitable against cervical cancer proteins. The interaction fingerprints have revealed that PHE, VAL, SER and ALA are the residues among most interactions. We also explore the stability of the multitargeted potential of Pixantrone Maleate through 100ns MD simulations and investigate the RMSD, RMSF and intermolecular interactions between all three proteins-ligand complexes. All computational studies favour Pixantrone Maleate as a multitargeted inhibitor of the TBK1, DNA polymerase epsilon, and integrin α-V β-8 and can be validated experimentally before use.

## 1. Introduction

Cervical cancer, a pervasive global health menace, has cast a long and dark shadow across the lives of countless women, emerging as a poignant symbol of medical challenges and global health disparities [1]. This disease, primarily driven by persistent infections with high-risk human papillomaviruses (HPVs), has evolved into a silent global pandemic, affecting women predominantly in low- and middle-income countries where access to healthcare resources is often limited. It accounts for a significant portion of cancer-related deaths among women worldwide, emphasising the urgent need for innovative therapeutic strategies that can effectively combat its devastating effects [2, 3]. While significant progress has been made in understanding the biology of cervical cancer, single-targeted therapies have encountered a formidable adversary in drug resistance, underscoring the imperative for a paradigm shift in our approach [4, 5]. Within the intricate landscape of cervical cancer biology, three proteins have emerged as pivotal players, intricately woven into the fabric of this malignancy: epsilon B-subunit of DNA polymerase, Integrin alpha-v beta-8 and Human TBK1 (TANK-binding kinase 1) [6–8]. These proteins, each with its unique role and significance, contribute to the complex tapestry of cervical carcinogenesis. DNA polymerase epsilon B-subunit, a key player in DNA replication, repair, and cell cycle regulation, plays an important role in maintaining the integrity of the genetic code, and dysregulation is a well-documented phenomenon in various cancers; cervical cancer is no exception [6, 9, 10]. Aberrant expression or mutations in this protein can lead to unchecked cell division and the accumulation of genetic alterations, driving the disease’s progression. Human TBK1, a component of the innate immune response, has recently emerged as a potential protagonist in cervical cancer. While it was traditionally associated with antiviral defence mechanisms, growing evidence suggests its involvement in oncogenic transformation and tumour progression. TBK1’s intricate relationship with inflammation, cell proliferation, and evasion of immune surveillance adds a multifaceted dimension to its role in cervical cancer [8]. Understanding the nuances of its participation in the disease could unveil novel therapeutic opportunities. Integrin alpha-v beta-8, our third protagonist, is a cell surface receptor intimately involved in cell adhesion, signalling, and interactions with the extracellular matrix [7]. DNA polymerase epsilon B-subunit, responsible for maintaining genomic integrity, influences DNA repair mechanisms, thus impacting the stability of cervical cells [6]. This, in turn, can activate signalling pathways involving TBK1, potentially fostering a pro-inflammatory environment that promotes tumour growth. Integrin alpha-v beta-8, with its role in tumour cell adhesion and motility, can further enhance the metastatic potential of cervical cancer cells, exacerbating the disease’s aggressiveness, which interplay among the proteins underscores the need for a comprehensive, multitargeted approach [1, 7, 8, 11–13].

A multitargeted drug design approach offers a promising strategy in the battle against cervical cancer that simultaneously can target epsilon B-subunit of DNA polymerase, Integrin alpha-v beta-8, and Human TBK1, making it possible to disrupt multiple pro-cancer signalling pathways [6–8]. This approach holds the potential for enhanced therapeutic efficacy, reduced likelihood of drug resistance, and a more holistic assault on cervical cancer. It signifies a shift towards precision medicine, where treatments are tailored to address the intricate web of factors driving the disease’s progression. The triumvirate of epsilon B-subunit of DNA polymerase, Integrin alpha-v beta-8 and Human TBK1 play central roles in the complex narrative of cervical carcinogenesis [6–8]. Their interconnectedness within the disease’s biology calls for a multitargeted drug design approach that can simultaneously disrupt multiple facets of the disease [14–17]. Combining therapies targeting DNA polymerase epsilon B-subunit, Human TBK1, and Integrin alpha-v beta-8 could disrupt the pro-cancer signalling cascades while simultaneously addressing cervical cancer’s genomic instability and promising enhanced efficacy and reduced drug resistance [6–8, 18, 19].

In this study, we performed multitargeted docking studies of prepared and validated proteins and ligands to identify a compound. Also, we have performed pharmacokinetics and fingerprint studies that favoured the results and the complex was validated for its stability with MD simulation studies, and the deviation, fluctuations and intermolecular interactions were analysed.

## 2. Methods

**Fig 1:**
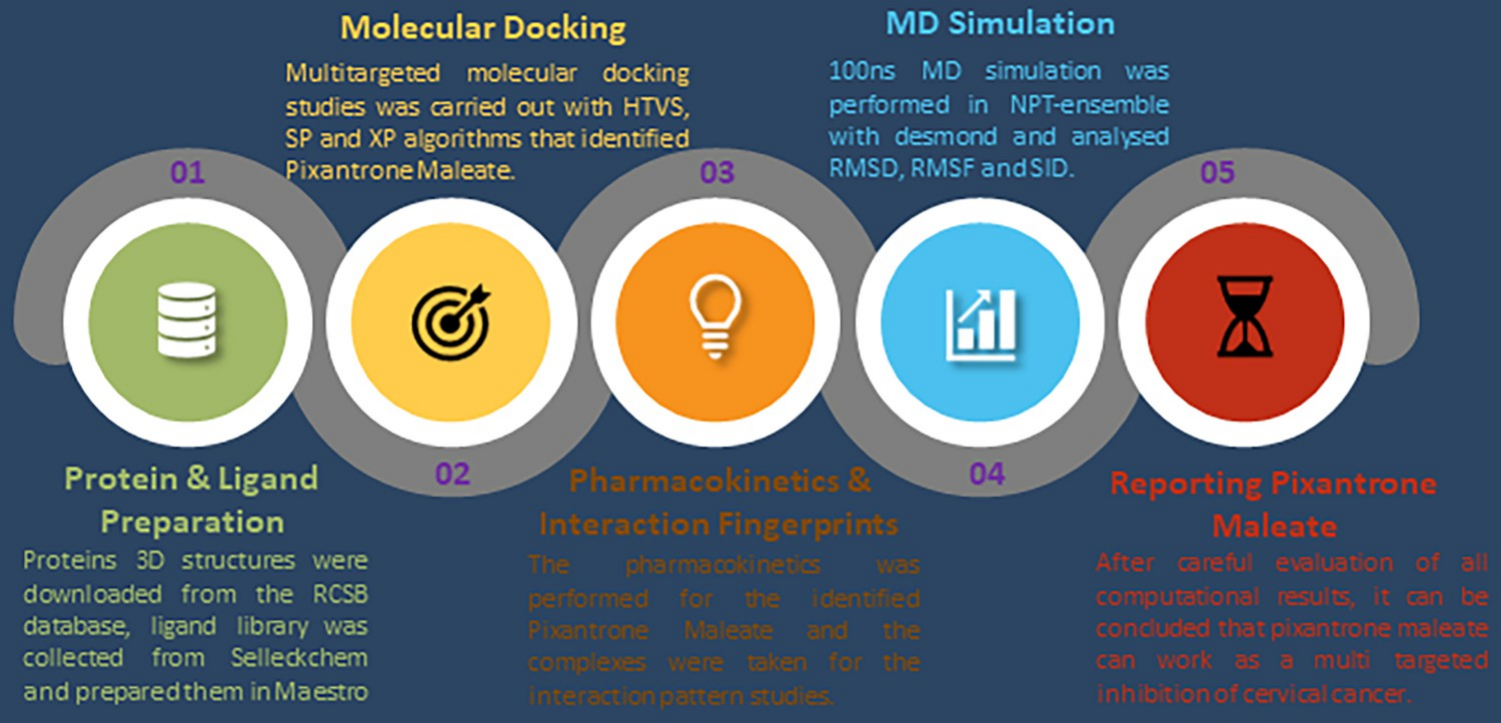
The flow of the study shows the complete flow from the protein-ligand collection preparation to computational identification and validation of the results.

The complete methods can be understood in Fig 1, which shows the complete flow, and the detailed followed methods in this study are as follows-

### 2.1 Ligand library and protein preparation

The ligand library was obtained from the website https://www.selleckchem.com/ and processed using Schrodinger’s Maestro software with the LigPrep tool (https://schrodinger.com/) [20, 21]. The FDA-approved drugs were chosen as they offer several advantages of being evaluated by rigorous safety and efficacy evaluations, well-documented pharmacological profiles, and known mechanisms of action provides valuable insights into potential interactions with target proteins and can expedite the drug development process, reducing time and costs. These drugs often have established manufacturing and formulation processes, facilitating their transition into clinical trials. In the LigPrep, we kept the atoms filter size of 500 atoms to screen only small molecules and used the OPLS4 forcefield [20, 22, 23]. Ionization was maintained to produce a potential state at a specified pH range of 7 ± 2. The Epik tool introduced a metal binding state while preserving the initial state [20, 24]. The tautomers generated during desalting were retained, and computations for stereoisomers with specified chiralities were performed, resulting in 32 stereoisomers per ligand. The resulting data was saved in SDF format. Protein structures were obtained from the RCSB database (http://rcsb.org/) [25, 26]. We identified PDBIDs for DNA polymerase epsilon B-subunit (PDBID: 5VBN), human TBK1 (PDBID: 6NT9) and integrin alpha-v beta-8 (PDBID: 6UJB) and downloaded and prepared using Protein Preparation Workflow tool (PPW) of Maestro (https://schrodinger.com/)[6–8, 20, 27, 28]. Preparing the 3D structures from the PDB (Protein Data Bank) database is crucial before molecular docking, as the raw PDB files may contain water molecules, ligands, or other nonessential components that need removal to focus on the specific protein-ligand interaction of interest. The structures may have missing atoms, incomplete residues, or structural irregularities that often require correction or refinement to ensure accuracy and energy minimisation may be necessary to optimise the protein’s geometry [29–31]. PDBID: 5VBN and 6UJB contain chains A, B, E, and F of proteins, solvents, and metals/ions, and 6NT9 contains chains A, B, and ligands [6–8]. In preprocessing, we capped the termini, filled in missing loops and side chains using Prime Module, assigned bond orders using the Chemical Compound Database (CCD) database, replaced hydrogens, created disulphide bonds and zero bond orders to metals and converted selenomethionines to methionine, generated heterostate using Epik at pH 7.4 ±2 [20, 24, 32]. In the section dedicated to optimizing hydrogen bond assignments, sample water orientations, considered crystal symmetry, examined minimised hydrogen configurations of modified species, and performed optimization utilizing PROPKA. In the minimisation and deleting water tab, converting heavy atoms to RMSD of 0.30 Å, deleting water beyond 5Å of ligand, and using OPLS4 forcefield were checked [20, 22]. The same parameter was kept for each case, and after preparation, we kept Chain A in 6NT9, Chain A and B in 6UJB and Chain A, B, E and F in 6UJB and these proteins were checked with Ramachandran Plot to check if the proteins were prepared.

### 2.2 Molecular docking studies

Generating a grid is essential for molecular docking because it defines the search space in which the ligand will interact with the target protein, and it restricts the exploration of possible binding orientations, making the docking process computationally efficient and ensuring the accurate prediction of ligand-protein interactions. We employed the Receptor Grid Generation (RGG) (https://www.schrodinger.com/products/glide) tool to create grids for each protein. We maintained a scaling factor of 1.0 and a partial charge cutoff of 0.25 [20, 33, 34]. In the site tab, we set the bounding box such that the docked ligand was confined within it, and the box was centred on the selected residues. We specified all residues to generate the grid for the entire protein. The box size was adjusted with dock ligands with length and advanced settings, and the rest of the advanced setting was kept default. Molecular docking is crucial for drug design because it predicts how potential drug compounds interact with specific target proteins at the molecular level. It helps identify candidate molecules with high binding affinity and optimal geometries for therapeutic efficacy, and it accelerates drug discovery by narrowing down the pool of compounds for experimental testing, reducing costs, and increasing the likelihood of developing effective medications to treat various diseases. We used the Virtual Screening Workflow (VSW) tool to screen the FDA-approved library, where the ligand source was provided with the prepared ligand library and combined to remove the duplicates and distribute for the sub-jobs [20, 33, 34]. In the filtering tab, we computed the pharmacokinetic properties and then filtered them with Lipinski’s rule, and the compounds not satisfying the criteria were filtered out [20, 35, 36]. We did not use the filtering tab since our library was already prepared. Instead, in the receptor tab, we examined the grid file generated with the RGG tool. Moving on to the docking tab, we employed the Epik state penalties for docking. We recorded interaction scores for residues within a 12Å radius of the grid centre, using a scaling factor of 0.80. Additionally, we maintained a partial charge cutoff of 0.15 [20, 24]. Docking experiments were conducted using the High Throughput Virtual Screening (HTVS), Standard Precision, and Extra Precision (XP) docking algorithms. Subsequently, the generated poses underwent filtration using the Molecular Mechanics-based Generalised Born Surface Area (MM/GBSA) algorithm [20, 32–34]. The highest-scoring 10% of HTVS data was transferred to SP, and a similar process was carried out when passing it to XP. In XP, we also applied a method to generate up to 4 possible positions for each compound state. Subsequently, all of the data from XP, totalling 100%, was forwarded to MM/GBSA to calculate binding free energy levels [32]. The same parameter was followed for each condition, and then we exported the data to CSV for analysis to identify multitargeted docking [37–41].

### 2.3 Pharmacokinetics and QM-based DFT studies

Pharmacokinetics properties of a drug candidate encompass its absorption, distribution, metabolism, and elimination within the body. Understanding these properties is crucial for assessing how the drug is absorbed, distributed to target tissues, metabolised, and eventually eliminated, influencing its bioavailability, half-life, and overall effectiveness and safety in clinical use [42]. For the computations of the pharmacokinetics properties of the identified compound, we used the QikProp tool (https://www.schrodinger.com/products/qikprop) and took the standard values to compare them to match if our compound satisfies the criteria of being a drug candidate [20, 35, 36]. We employed the Jaguar (https://www.schrodinger.com/products/jaguar) Optimization tool to fine-tune our chosen molecule for optimisation and used the default B3LYP-D3 theory and a 6-13G*** basis set for Density Functional Theory, keeping the SCF spin treatment on automatic mode [20, 43, 44]. In the initial guess, we set the SCF tab to quick and atomic overlap. For convergence criteria, we set a maximum of 48 iterations, an energy change of 5e-5 Hartree, and an RMS density matrix change of 5e-06. We utilised an SCF level shift of 0.0 Hartree to maintain stability and opted for no thermal smearing. Our convergence scheme was DIID, and we ensured that consistent orbital sets were used when input structures were isomers with the same basis set [45]. We allowed 100 steps in the optimisation tab with default criteria and used Schlegel’s guess and redundant internal coordinates for the initial hessian. For property calculations, we selected all properties with vibration frequencies at thermochemistry conditions of 1.0 atm pressure and an initial temperature of 298.15K in Kal/mol units. Surface calculations included electrostatic potential with no specified box size and an average local ionisation energy of 5 pts/Å Kcal/mol. We also considered noncovalent interactions with a grid density of 20 pts/Å and focused on density and spin density. Molecular Orbitals were set from α HOMO-0 to LUMO+0 with 2 orbitals and β HOMO-0 to LUMO+0 with the same two orbitals. Solvation was addressed using the PBF model in a water solvent medium, and the output was saved in Gaussian format after the computations. We utilised the QM convergence monitor tool in the Jaguar module to evaluate the results, locating the job directory for analysis [20, 43].

### 2.4 Molecular interaction fingerprints

The Molecular Interaction Fingerprints is a computational method to characterise and visualise the types and patterns of molecule interactions, typically in the context of molecular docking or drug discovery. They provide insight into how molecules interact, highlighting key residues or features involved in binding and aiding in designing potential drug candidates. We used the Interaction Fingerprints tool, where 6UJB was kept as a reference to align all three P-L complex sequences where we selected the option of receptor-ligand complexes [20]. We opted for the default settings when specifying the bond distance and proceeded to generate the fingerprints. Within the resulting interaction matrix, our choice was to highlight the connection from the N to C terminal of the proteins while retaining only the interacting residues. This decision was made to ensure an unambiguous plot. We also have shown the ligand interaction count and residues interacting with the ligand to understand the interaction pattern [20, 46].

### 2.5 System builder and molecular dynamics simulation studies

Molecular Dynamics (MD) Simulation is a computational method for investigating molecules’ and atoms’ dynamic interactions and motions throughout a given timeframe. It simulates their movements and interactions according to physical laws, providing insights into the structure, stability, and properties of biological macromolecules or materials, aiding in drug design. For molecular dynamics simulation, we used the Desmond package developed by D E Shaw Research (https://www.deshawresearch.com/), available freely for academic use [20, 47]. Before the production run, we needed to make the P-L complex in the solute system in a neutral state, and for that, we used the System Builder tool. The SPC water model was chosen for simulation within a rectangular box with dimensions of 10 × 10 × 10 Å. Any salt and ions were kept at least 20 Å away from the system during setup, and additional ions were introduced to maintain system neutrality. For neutralising the system, we added 12Na^+^ in 5VBN, 4Na^+^ in 6UJB, and 5Na^+^ in 6NT9, and then we minimised the volume and appropriately fixed at the P-L complexes and used the OPLS4 forcefield and kept the jobs to generate the system files [20, 22]. We conducted the production run using the Molecular Dynamics panel. In this run, we loaded the system builder file and set the simulation time to 100 nanoseconds, with a recording interval of 100 picoseconds and an energy level of 1.2, generating 1000 frames. We utilized the NPT-ensemble class, maintaining a temperature of 300 Kelvin and a pressure of 1.01325 bar, and we relaxed the system before starting the production run [20, 48]. Following the 100 nanoseconds MD simulation production, we employed the Simulation Interaction Diagram tool to analyze the trajectories. We then exported the data and figures to examine deviations, fluctuations, and intermolecular interactions.

## 3. Results

### 3.1 Protein preparation and structural studies

**Fig 2:**
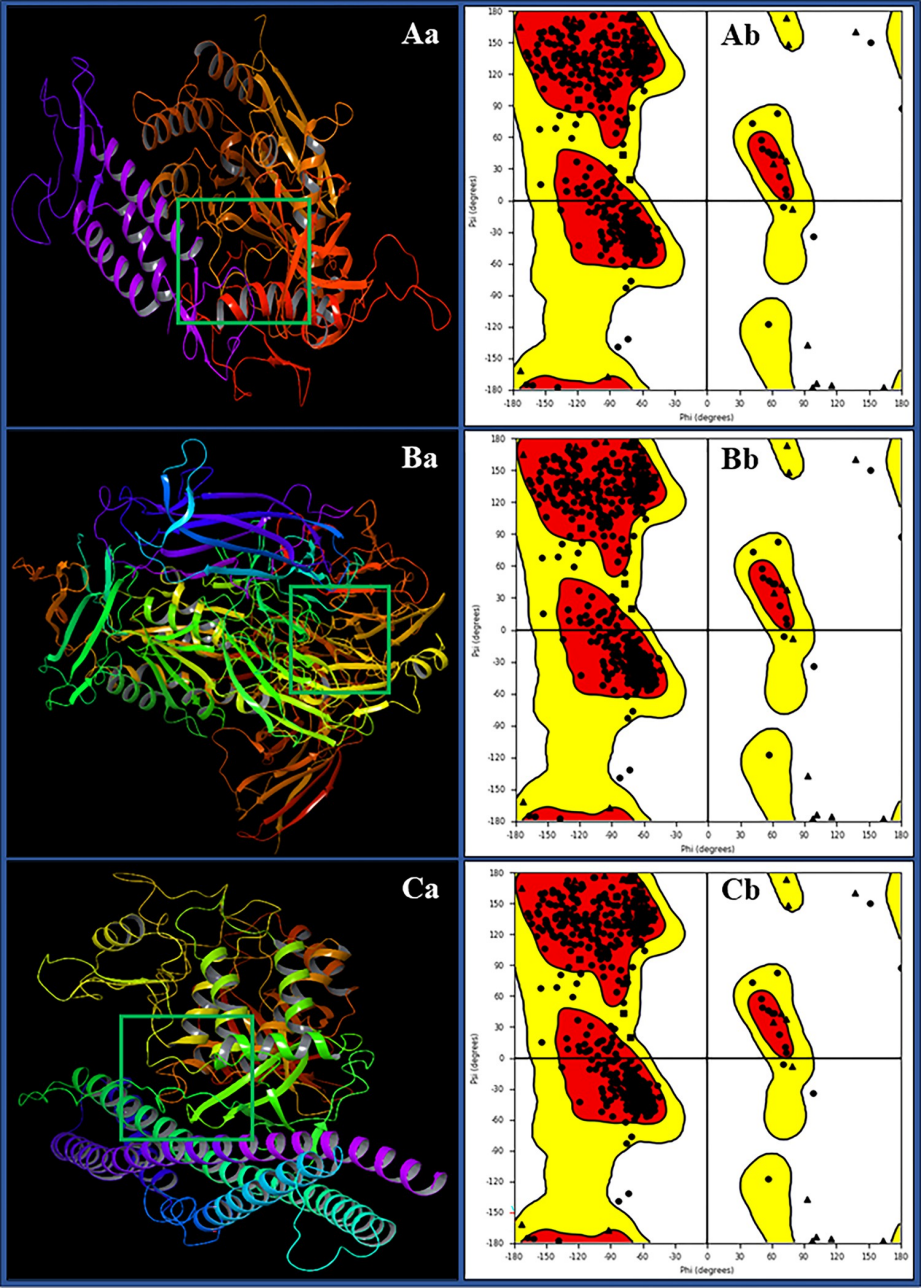
Prepared 3D structure and ligand binding site of **Aa)** DNA polymerase epsilon subunit 2 (PDBID: 5VBN), **Ba)** Integrin alpha-v beta-8 (PDBID: 6UJB) and **Ca)** TBK1 (PDBID: 6NT9), and Ramachandran plot for **Ab)** DNA polymerase epsilon subunit 2 (PDBID: 5VBN), **Bb)** Integrin alpha-v beta-8 (PDBID: 6UJB) and **Cb)** TBK1 (PDBID: 6NT9).

The meticulous use of the protein preparation workflow tool was instrumental in ensuring the accuracy and quality of the protein structures for our molecular docking studies involving three essential proteins are- DNA polymerase epsilon B-subunit (PDBID: 5VBN), Human TBK1 (PDBID: 6NT9), and Integrin alpha-v beta-8 (PDBID: 6UJB). This comprehensive process involved critical steps aimed at addressing structural irregularities and optimising the overall geometry of these proteins. Notably, a thorough check of the structures in Maestro revealed no errors, providing robust confirmation of the integrity of our preparation process. In Fig 2, we have shown the 3D structure, ligand binding site, and Ramachandran plot for each protein to understand its accuracy. Beginning with DNA polymerase epsilon B-subunit (5VBN), we noted a well-distributed distribution of residues throughout the protein’s structure. Further validation through the Ramachandran plot demonstrated that the vast majority of phi and psi angles fell within acceptable ranges, underscoring the high-quality preparation of this protein. Similarly, our assessment of Human TBK1 (6NT9) revealed consistent residue distribution, and the Ramachandran plot verified the appropriate conformation of phi and psi angles. These results instilled confidence in the structural reliability of both proteins, establishing a solid foundation for subsequent research. In the case of Integrin alpha-v beta-8 (6UJB), a complex structure involving multiple chains (A, B, E, and F), metals/ions, and solvents presented a unique challenge. However, our protein preparation workflow adeptly addressed these intricacies. Residue distribution analysis across the structure demonstrated uniform coverage, reassuring us of the thoroughness of our preparation. Notably, a meticulous examination of the Ramachandran plot reaffirmed that the phi and psi angles for all chains (A, B, E, and F) adhered to acceptable parameters, further validating the precision of our preparation process. The Protein Preparation Workflow (PPW), encompassing vital steps such as capping termini, modelling loops and side chains, assigning bond characteristics, adding hydrogen atoms, and conducting energy minimisation, was meticulously executed for all three proteins. The confirmation from Ramachandran plots for these proteins attested to their correctness and highlighted their quality, showcasing well-distributed residues and appropriate conformations of phi and psi angles. These rigorously prepared protein structures are a dependable foundation for our upcoming molecular docking and drug design studies. They ensure the utmost accuracy and integrity in our subsequent simulation experiments, allowing us to delve deeper into understanding these proteins’ interactions and their potential implications for bioinformatics and therapeutic development.

### 3.2 Molecular interaction studies

**Fig 3:**
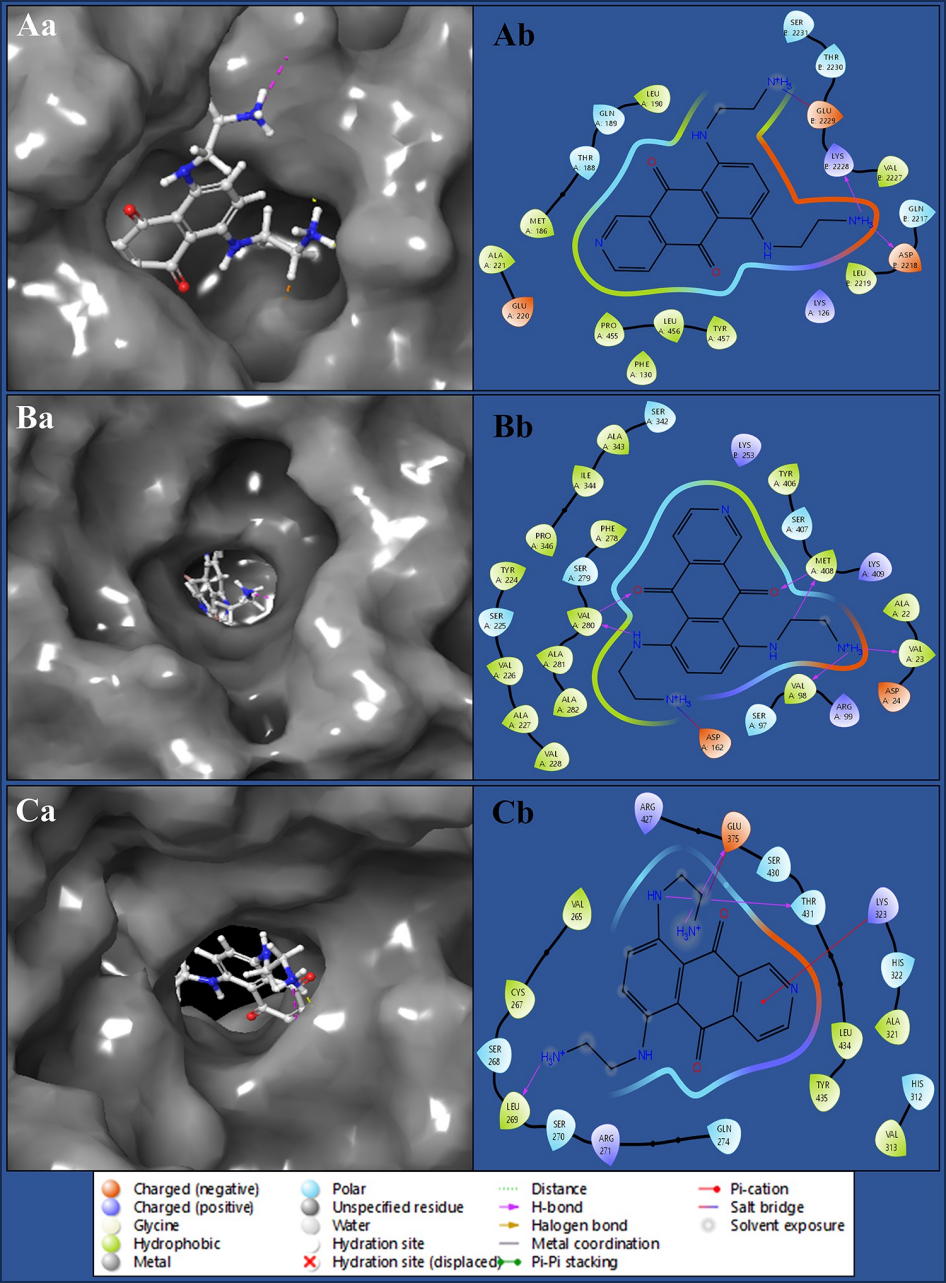
Showing the docked 3D poses Pixantrone Maleate in complex with **Aa)** DNA polymerase epsilon subunit 2 (PDBID: 5VBN), **Ba)** Integrin alpha-v beta-8 (PDBID: 6UJB) and **Ca)** TBK1 (PDBID: 6NT9), and docked 2D poses of **Ab)** DNA polymerase epsilon subunit 2 (PDBID: 5VBN), **Bb)** Integrin alpha-v beta-8 (PDBID: 6UJB) and **Cb)** TBK1 (PDBID: 6NT9).

Molecular docking, a cornerstone of drug discovery and computational biology, is pivotal in understanding the interactions between ligands and target proteins at the atomic level. This method simulates how these molecules fit together, predicting ligands’ binding affinity and orientation within protein binding sites and aids in exploring the mechanisms of biomolecular recognition, elucidating the role of specific residues in binding, and rationalising the design of novel therapeutics. In our cervical cancer study, molecular docking helped identify Pixantrone Maleate in all three proteins, guiding the development of multitargeted therapies. The complex of Pixantrone Maleate with DNA polymerase epsilon subunit 2 (PDBID: 5VBN) has produced a docking score of -8.147 Kcal/mol and MM/GBSA of -40.55 Kcal/mol and formed two hydrogen bonds between the ASP2218, and LYS2228 residues with NH_3_ atom, as well as a salt bridge along GLU2229 residue with the NH_3_ atom (Fig 3A, Table 1). The Integrin alpha-v beta-8 (PDBID: 6UJB) in complex with Pixantrone Maleate has shown a docking score of -8.206 Kcal/mol and MM/GBSA of -33.67 Kcal/mol and formed the interaction of six hydrogen bonds connecting among VAL23, and VAL98 residues with NH_3_ atom, also both VAL280 and MET408 residues connects 2NH and 2O atoms and a salt bridge formed with ASP162 residue along the NH_3_ atom of the ligand (Fig 3B, Table 1). The complex of Pixantrone Maleate with TBK1(PDBID: 6NT9) has produced a docking score of -7.310 Kcal/mol and MMGBS/A of -37.64 Kcal/mol and involved 3H bonds among LEU269 and GLU375 residues with 2 NH_3_ atoms, THR431 residue interact NH atom and one salt bridge formed among GLU375 and NH_3_ atom, and a pi-cation bond contact with LYS323 residue along the benzene ring of the ligand (Fig 3C, Table 1). The molecular docking revealed strong binding affinities and specific interactions between Pixantrone Maleate and all three target proteins, DNA polymerase epsilon, Integrin alpha-v beta-8, and TBK1, involved in cervical cancer. These findings provide valuable insights into the potential of Pixantrone Maleate as a multitargeted inhibitor, supporting its further investigation and development as a therapeutic agent for cervical cancer.

**Table 1:** Showing the docking and MM\GBSA score along with other computations during molecular docking studies.

SNo	PDBID	Resolution	Docking Score	MMGBSA dG Bind	ligand efficiency sa	ligand efficiency ln	Evdw	Ecoul
**1**	5VBN	2.35 Å	-8.147	-40.55	-2.534	-9.706	-35.961	-13.943
**2**	6UJB	3.51 Å	-8.206	-33.67	-2.105	-8.06	-31.09	-20.577
**3**	6NT9	3.30 Å	-7.31	-37.64	-2.353	-9.009	-27.82	-24.152

### 3.3 Pharmacokinetics and QM-based DFT studies

The pharmacokinetics properties of a drug candidate encompass its absorption, distribution, metabolism, and elimination within the body. These properties determine how the drug is absorbed, distributed to target tissues, metabolised, and eventually removed from the body, influencing its bioavailability, half-life, and overall effectiveness and safety in clinical use. Pixantrone Maleate’s pharmacokinetic properties and descriptors have been analysed using the QikProp tool and compared with the standard values, providing valuable insights into its potential as a multitargeted drug candidate for cervical cancer. Pixantrone Maleate exhibits favourable pharmacokinetic characteristics in several key areas. It demonstrates moderate acidity, indicating its potential for efficient absorption in the body. The presence of two amine groups suggests opportunities for hydrogen bonding and enhanced interaction with target proteins, and it has an optimal number of rotatable bonds (8), which is advantageous for its binding flexibility within protein binding sites. The QikProp analysis reveals that Pixantrone Maleate has a low molecular weight (325.369 g/mol), within the desirable range for drug candidates. The compound exhibits a moderate dipole moment (2.207 D), indicating its potential to interact with charged regions in protein binding sites. It also shows a reasonable globularity value (0.825654), suggesting a well-balanced molecular shape. Pixantrone Maleate demonstrates favourable human oral absorption characteristics, estimated at 26.004%. While it falls slightly below the high absorption threshold of 80%, it indicates potential suitability for oral administration. The PSA (138.829 Å^2^) and QPlogS (-0.842) values suggest reasonable solubility, essential for effective drug delivery. In terms of blood-brain barrier permeability (QPlogBB), Pixantrone Maleate exhibits a value of -1.299, indicating a lower likelihood of central nervous system (CNS) penetration. This property is desirable for drugs targeting cervical cancer as it minimises potential CNS-related side effects. Pixantrone Maleate also complies with the Rule of Five, with a maximum of one violation, making it suitable for further drug development efforts. The Rule of Three is also met, indicating its potential as a lead compound (Table 2). The QikProp analysis suggests that Pixantrone Maleate possesses favourable pharmacokinetic properties, molecular characteristics, and drug-like qualities, making it a promising multitargeted drug candidate for cervical cancer. Its potential for efficient absorption, moderate molecular weight, solubility, and adherence to essential drug development rules underscore its suitability for further preclinical and clinical investigations.

**Table 2:** Showing the pharmacokinetics properties of Pixantrone Maleate against descriptors and standard values.

Descriptor	Standard Values	Pixantrone Maleate	Descriptor	Standard Values	Pixantrone Maleate
#acid	0–1	0	IP(eV)	7.9–10.5	7.953
#amide	0–1	0	Jm	N/A	0
#amidine	0	0	mol MW	130.0–725.0	325.369
#amine	0–1	2	%HumanOralAbs	>80% is high, <25% is poor	26.004
#in34	N/A	0	PISA	0.0–450.0	194.649
#in56	N/A	14	PSA	7.0–200.0	138.829
#metab	1–8	11	QPlogBB	−3.0–1.2	-1.299
#NandO	2–15	7	QPlogHERG	concern below −5	-6.734
#noncon	N/A	0	QPlogKhsa	−1.5–1.5	-0.361
#nonHatm	N/A	24	QPlogKp	−8.0 –−1.0	-8.295
#ringatoms	N/A	14	QPlogPC16	4.0–18.0	11.708
#rotor	0–15	8	QPlogPo/w	−2.0–6.5	-0.02
#rtvFG	0–2	0	QPlogPoct	8.0–35.0	20.63
#stars	0–5	1	QPlogPw	4.0–45.0	15.488
accptHB	2.0–20.0	7.5	QPlogS	−6.5–0.5	-0.842
ACxDN^.5/SA	0.0–0.05	0.0249135	QPPCaco	<25 poor, >500 great	4.764
CIQPlogS	−6.5–0.5	-1.554	QPPMDCK	<25 poor, >500 great	1.872
CNS	−2 (inactive), +2 (active)	-2	QPpolrz	13.0–70.0	32.275
dip^2/V	0.0–0.13	0.0046744	RuleOfFive	maximum is 4	1
dipole	1.0–12.5	2.207	RuleOfThree	maximum is 3	2
donorHB	0.0–6.0	4	SAamideO	0.0–35.0	0
EA(eV)	−0.9–1.7	1.668	SAfluorine	0.0–100.0	0
FISA	7.0–330.0	222.683	SASA	300.0–1000.0	602.083
FOSA	0.0–750.0	184.752	Type	N/A	Small
glob	0.75–0.95	0.825654	volume	500.0–2000.0	1042.16
HumanOralAbs	N/A	1	WPSA	0.0–175.0	0

**Fig 4:**
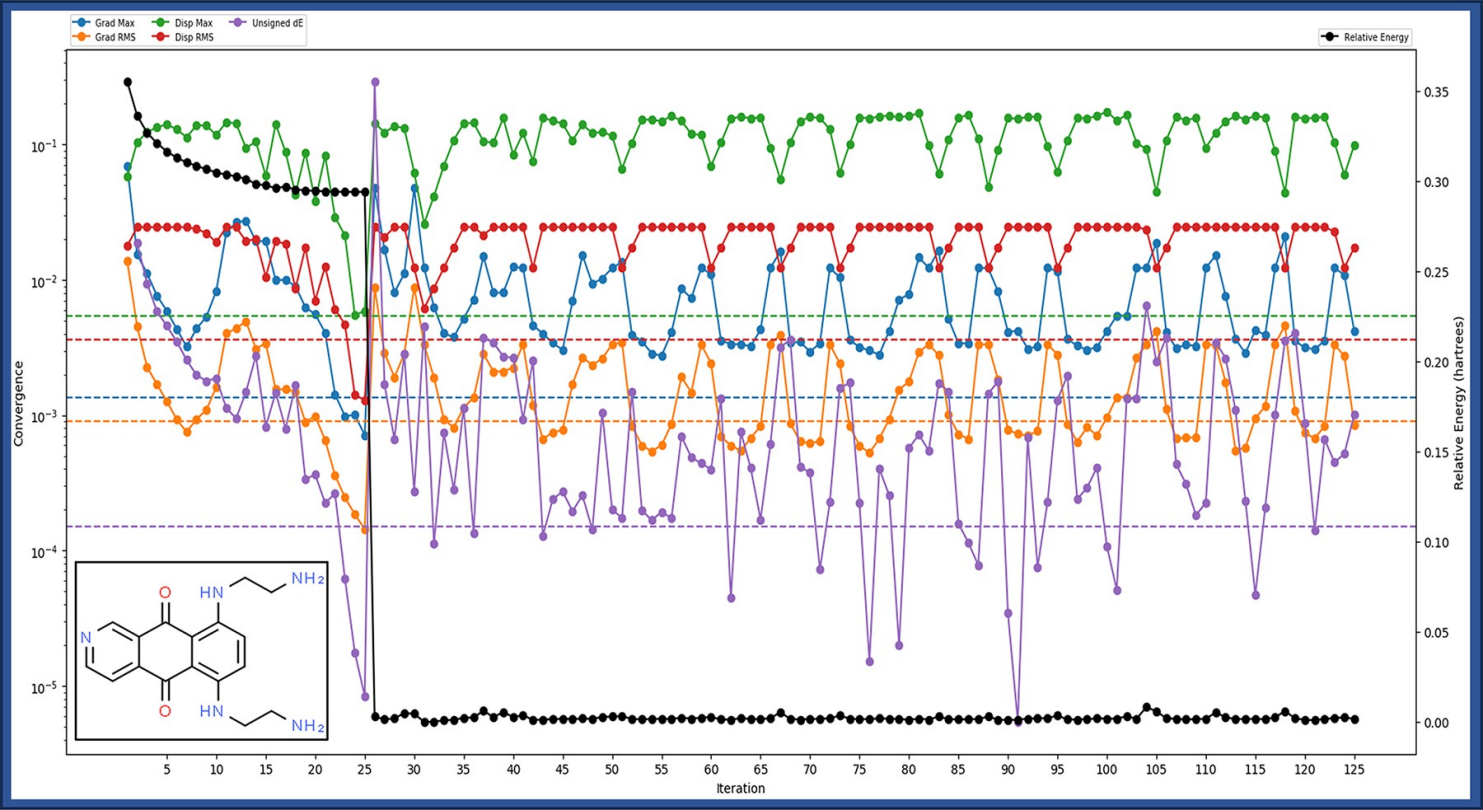
Showing the Quantum Mechanics-based Density Functional Theory computations of Pixantrone Maleate where Grad max, Disp Max, unsigned dE, Grad RMS and Disp RMS are in different colours while the relative energy is in black.

The data presented provides an in-depth characterisation of Pixantrone Maleate through Quantum Mechanics (QM) calculations using the Density Functional Theory (DFT) method with the B3LYP-D3 functional and a 6-31g** basis set. This analysis delves into various molecular properties of Pixantrone Maleate in the gas phase. It begins with determining 463 canonical orbitals, shedding light on the distribution of electrons within the molecule. The gas phase energy is calculated at -1084.190725 kcal/mol, a fundamental indicator of the molecule’s stability and reactivity. Further insights into the molecule’s electronic structure are gained through the examination of the Highest Occupied Molecular Orbital (HOMO) and Lowest Unoccupied Molecular Orbital (LUMO) energies, which stand at -0.410674 and -0.312769, respectively. These values offer valuable information regarding the potential for chemical reactions. Additionally, the molecule’s polarizability, with a value of 249.335, reveals its susceptibility to polarisation by external electric fields. The data also encompasses dipole moment information, providing the total dipole moment and its components in different directions (X, Y, and Z). These values elucidate the molecule’s polarity and its interactions with other molecules. Vibrational frequencies, including the lowest, highest, and second lowest, along with the count of negative frequencies, offer insights into Pixantrone Maleate’s vibrational properties and stability. The zero-point energy, calculated at 237.677 kcal/mol, accounts for quantum mechanical effects. Thermodynamic properties such as entropy (130.737 Kcal/mol/), enthalpy (11.435173 kcal/mol), free energy (-27.543965 kcal/mol), and internal energy (10.842687 kcal/mol) provide crucial information about the molecule’s behaviour at standard conditions (Fig 4). Heat capacity (72.388 Kcal/mol/) reveals how temperature changes the molecule’s heat content. Moreover, the data includes electrostatic potential (ESP) information, encompassing the minimum, maximum, mean, and local polarity. These values shed light on charge distribution and reactivity within the molecule. Additionally, Average Local Ionization Energy (ALIE) information offers insights into ionisation energy distribution, highlighting regions of high and low reactivity. This study is pivotal for comprehending its behaviour in various drug design application contexts.

### 3.4 Molecular interaction fingerprint studies

**Fig 5:**
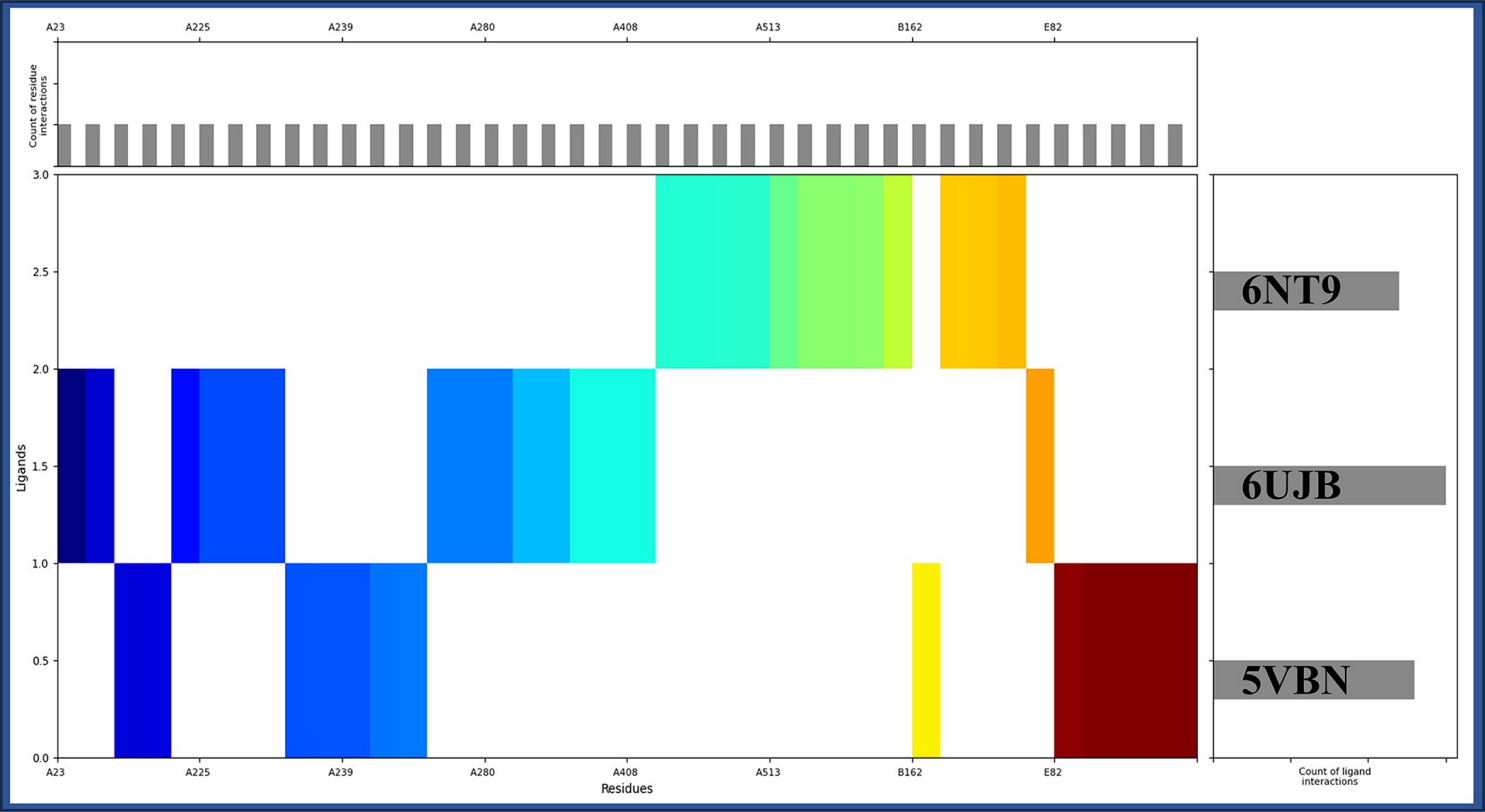
Showing the interaction fingerprints of the Pixantrone Maleate with DNA polymerase epsilon subunit 2 (PDBID: 5VBN), Integrin alpha-v beta-8 (PDBID: 6UJB) and TBK1 (PDBID: 6NT9) and colour shows the residue distribution from N to C terminal.

From the molecular interaction fingerprinting study, we identified most interacting residue with counts among all three proteins with the Pixantrone Maleate were 6PHE, 5VAL, 4SER, 4ALA, 3LYS, 3ASP, 2TYR, 2LEU, 2ILE, 1THR, 1PRO, 1MET, 1GLY, 1GLU, 1GLN, 1CYS, and 1ASN to make the complexes stable (Fig 5). The molecular interaction fingerprinting study provided valuable insights into Pixantrone Maleate’s interactions with the proteins involved in cervical cancer. Among all three proteins, namely DNA polymerase epsilon subunit 2 (PDBID: 5VBN), Integrin alpha-v beta-8 (PDBID: 6UJB), and TBK1 (PDBID: 6NT9), several residues exhibited prominent interactions with the compound, contributing to the stability of the P-L complexes and ligand displayed a robust affinity for specific amino acid residues across these proteins. Among these, phenylalanine [35] emerged as the most frequently interacting residue, forming six interactions. Valine [2] followed closely with five interactions, emphasising its significance in stabilising the complexes. Serine (SER) and alanine [16] also displayed substantial interactions, each contributing four stabilising contacts. The amino acids lysine (LYS) and aspartic acid exhibited three interactions each, further reinforcing the stability of the complexes. Tyrosine (TYR), leucine (LEU), and isoleucine (ILE) demonstrated two interactions each, while threonine (THR), proline, methionine (MET), glycine (GLY), glutamic acid (GLU), glutamine (GLN), cysteine (CYS), and asparagine (ASN) contributed one interaction each. These interactions collectively create a network of stabilising forces within the protein-ligand complexes, enhancing their overall stability and potential efficacy. The specific residues involved in these interactions play crucial roles in maintaining the integrity of the complexes and facilitating the binding of Pixantrone Maleate to its target proteins. This molecular interaction fingerprinting analysis underscores Pixantrone Maleate’s suitability as a multitargeted drug candidate for cervical cancer.

### 3.5 Molecular dynamics simulation studies

Molecular Dynamics (MD) simulation is a computational technique that models the dynamic behaviour of molecules over time and calculates the movement of atoms and molecules, simulating their interactions under controlled temperature and pressure and helps to understand the conformational changes, stability, and interactions. The system builder file has generated 73197 atoms for 5VBN in complex with Pixantrone Maleate, 140861 atoms for 6UJB in complex with Pixantrone Maleate and 110141 atoms for 6NT9 in complex with Pixantrone Maleate. Further, the system builder files were used for the production run, and the generated trajectories were analysed extensively to interpret the deviation, fluctuations and intermolecular interactions.

#### 3.5.1 Root Mean Square Deviation

**Fig 6:**
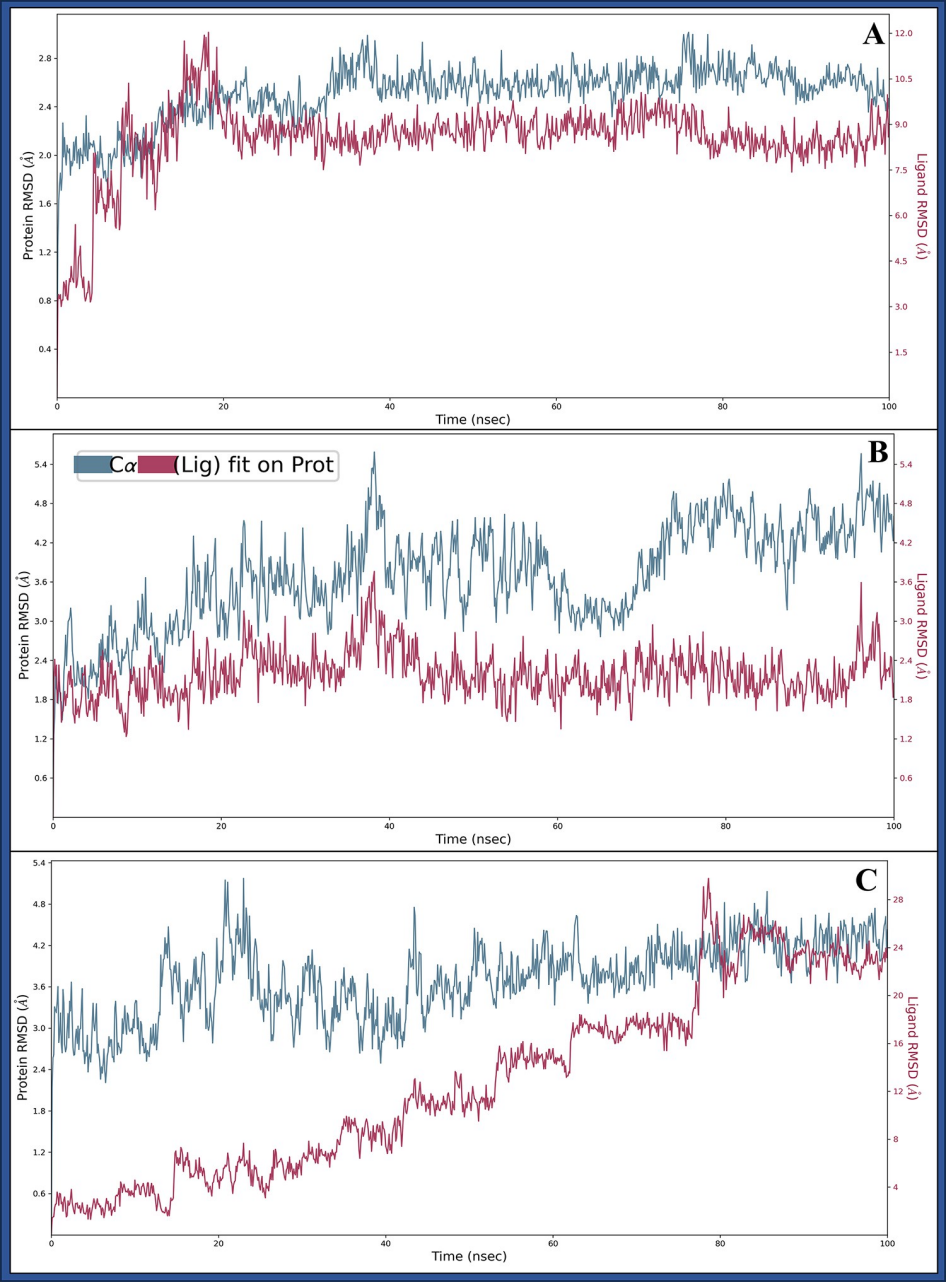
Showing the Root Mean Square Deviation (RMSD) of Pixantrone Maleate [2] in complex with **A)** DNA polymerase epsilon subunit 2 (PDBID: 5VBN), **B)** Integrin alpha-v beta-8 (PDBID: 6UJB) and **C)** TBK1 (PDBID: 6NT9) in blue.

Root Mean Square Deviation (RMSD) is a metric used in structural biology and molecular dynamics simulations to measure the difference between the positions of atoms or molecules at different time points. It quantifies how much a molecule’s structure varies compared to a reference structure that helps to assess the stability and structural changes of proteins and ligands, aiding in analysing stability. The complex of DNA polymerase epsilon subunit 2 (PDBID: 5VBN) with Pixantrone Maleate initially deviated, the protein showed 1.24 Å, and the ligand showed 2.16 Å at 0.10 ns, and then the entire simulation exhibited a stable performance after ignoring the initial 1ns. The protein deviated to 2.47 Å, and the ligand deviated to 8.58Å at 100ns, meaning the proteins were stable, and the ligand showed the deviation either because of sudden heat or a change in solute medium (Fig 6A). The Integrin alpha-v beta-8 (PDBID: 6UJB) in complex with Pixantrone Maleate has shown some stability after initial deviation, which was noted for the protein of 1.26 Å, while for the ligand, it was on 2.09 Å at 0.10ns and at 100 ns, the proteins have deviated to 4.23 Å whereas the ligand deviated to 1.84 Å, indicating stability and dependability in this simulative period and making the complex most stable among all (Fig 6B). TBK1(PDBID: 6NT9) in complex with Pixantrone Maleate shows the initial deviation of 2.14Å and 1.40Å at 0.10 ns for the proteins and ligand, respectively. After ignoring the first 1ns complex during simulation, the protein deviated to 4.10Å at 100 ns, demonstrating stability and dependability (Fig 6C). These RMSD results confirm the overall stability of the protein-ligand complexes formed with Pixantrone Maleate. While there were initial fluctuations, likely due to system equilibration, the complexes reached stable states, indicating the reliability of these interactions. These findings highlight the potential of Pixantrone Maleate as a promising multitargeted drug candidate for cervical cancer, as it forms stable complexes with key proteins.

#### 3.5.2 Root Mean Square Fluctuations

**Fig 7:**
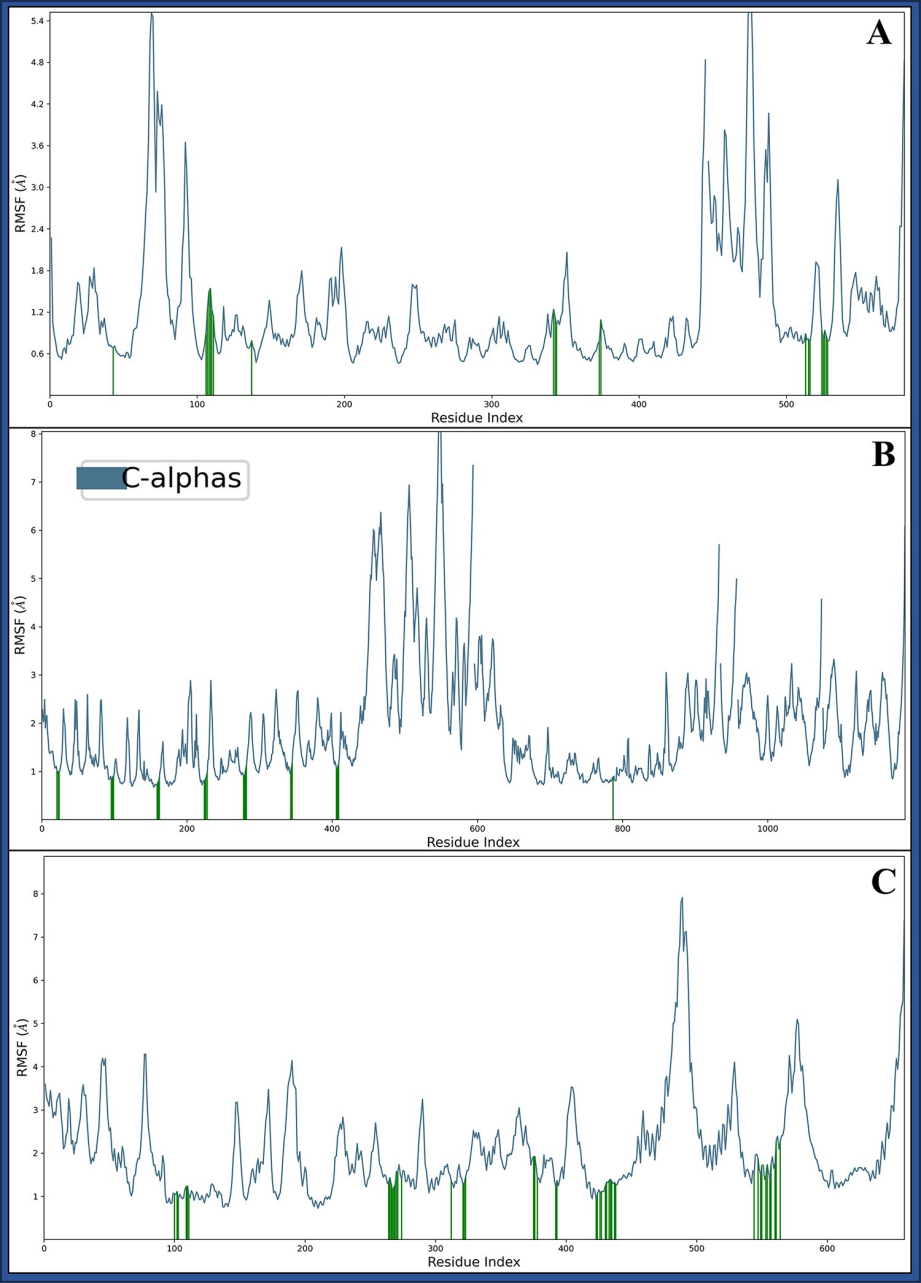
Showing the Root Mean Square Fluctuations (RMSF) of **A)** DNA polymerase epsilon subunit 2 (PDBID: 5VBN), **B)** Integrin alpha-v beta-8 (PDBID: 6UJB) and **C)** TBK1 (PDBID: 6NT9) in blue and green line shows the Pixantrone Maleate contact with proteins.

The Root Mean Square Fluctuation (RMSF) is a structural biology and molecular dynamics simulation measure that assesses the flexibility or fluctuation of individual atoms within a molecule over time. It quantifies the deviations from the average positions of atoms, highlighting regions of high flexibility or rigidity. RMSF is crucial for understanding the dynamic behaviour of biomolecules, identifying important binding sites, and evaluating structural stability during simulations, aiding drug discovery and structural biology studies. The complex DNA polymerase epsilon subunit 2 (PDBID: 5VBN) with Pixantrone Maleate has shown a few residues fluctuating beyond 2 Å are HIS84, PHE147-SER161, GLY173-LYS177, GLU281, ASN434, GLY526, and PHE527 and many residues were forming interaction with the ligand are LYS126, GLN189-LYS194, GLU220, ARG425-CYS427, LEU456, TYR457, LEU2216-LEU2219, and VAL2227-SER2231 (Fig 7A). The Integrin alpha-v beta-8 (PDBID: 6UJB) in complex with Pixantrone Maleate has shown a few residues fluctuating beyond 2 Å are PHE1-SER31, THR46, PRO48, GLY49, SER63, ALA81-ASP83, MET118, THR134, ILE202-ASN206, ALA213, ASN232-GLY235, GLY287-ASP289, SER305, ASP306, ARG321-ASP325, ASP351-GLY354, ARG379-GLY382, ARG398, SER399, THR412, and ARG438-LEU593, and many residues were forming interaction with ligand are PHE21, VAL23, ASP24, ALA96-ARG99, PHE159-ASP162, TYR224-VAL228, PHE278-ALA282, ALA343-ALA345, TYR406-LYS409, and LYS253 (Fig 7B). The TBK1 (PDBID: 6NT9) in complex with Pixantrone Maleate has shown a few residues fluctuating beyond 2 Å are MET1-LEU34, VAL39-GLN52, ARG54, GLU57, LYS60, LYS61, GLU74-GLN150, GLN169-ASP193, LYS196, GLU225-ASN230, GLU232, GLY240, SER243, LYS251-PRO256, ALA287-LYS291, SER328-ILE334, GLU337, LYS341, LYS344, ILE346-ASN349, LEU359-LYS372, PRO400-ASP411, ASN455 and a few more and many residues were forming interaction with the ligand are GLU100, SER102, ASN103, GLU109-GLU111, PRO264-ARG271, GLN274, HIS312, ALA321-LYS323, GLU375, GLU376, PRO378, LEU392, ILE393, CYS423, TYR424-ARG427, SER430, THR431, LEU433-TYR435, GLU437, LEU438, PRO544, ARG547, VAL549, GLU550, GLN553, VAL554-ASN557, THR560, GLU561, and TYR564 to make the complex stable (Fig 7C). Various factors, including temperature changes or alterations in the solute medium, may influence these fluctuations. Despite these fluctuations, numerous residues interact with Pixantrone Maleate, contributing to the overall stability of the complex. These residues, distributed across different protein regions, contribute to its dynamic behaviour during the simulation. However, the complex remains stable, thanks partly to the interactions between many residues and the ligand Pixantrone Maleate. These interactions, including hydrogen bonds, salt bridges, and pi-cation contacts, are crucial in stabilising the complex and ensuring its structural integrity. The observed fluctuations in these residues may be attributed to the dynamic nature of biomolecules in solution, where thermal energy leads to atomic movements. Solvent effects and transient interactions with neighbouring molecules can also influence these fluctuations. Understanding these dynamic behaviours is essential for comprehending the structural and functional aspects of protein-ligand complexes and aids in the rational design of effective drug candidates.

#### 3.5.3 Simulation Interaction Diagram

**Fig 8:**
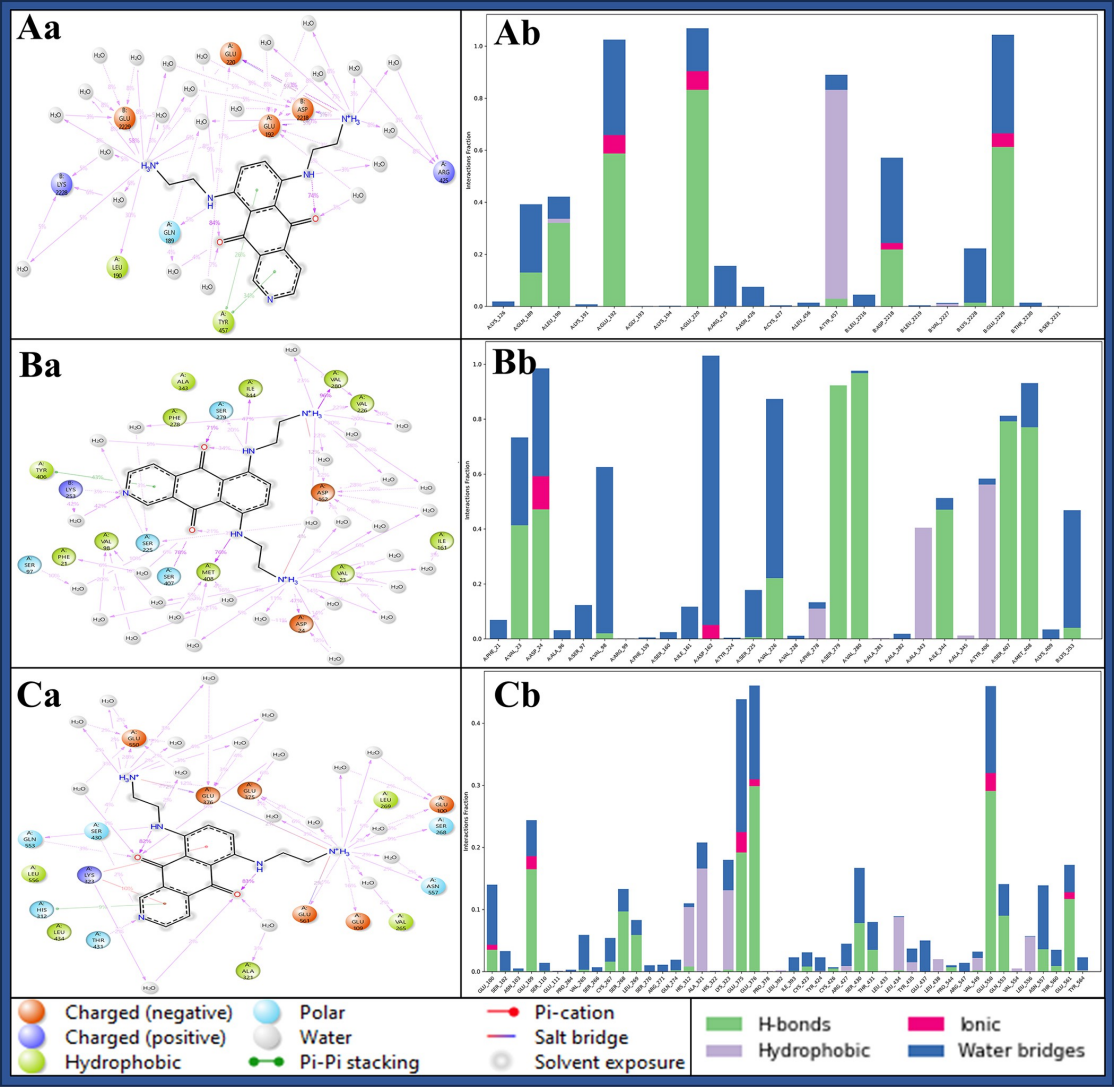
The Simulation Interaction Diagram (SID) of Pixantrone Maleate in complex with **Aa)** DNA polymerase epsilon subunit 2 (PDBID: 5VBN), **Ba)** Integrin alpha-v beta-8 (PDBID: 6UJB) and **Ca)** TBK1 (PDBID: 6NT9) and histogram representation of count of interactions of Pixantrone Maleate in complex with **Ab)** DNA polymerase epsilon subunit 2 (PDBID: 5VBN), **Bb)** Integrin alpha-v beta-8 (PDBID: 6UJB) and **Cb)** TBK1 (PDBID: 6NT9) where green shows the hydrogen bonds, red shows the ionic bonds, grey shows the hydrophobic bonds and blue shows the water bridges.

The Simulation Interaction Diagram (SID) is a visual representation used in molecular dynamics simulations to illustrate the interactions between atoms or molecules within a system. It provides insights into the various forces, such as hydrogen bonds, van der Waals, and electrostatic interactions, between particles during the simulation and helps to understand the complex interactions driving molecular behaviour and stability. In interaction among DNA polymerase epsilon subunit 2 (PDBID: 5VBN) and Pixantrone Maleate involves fourteen water molecules which act as a water bridge to provide stability and LEU190, GLU2229, ASP2218, GLU220, and GLU192 residues have formed hydrogen bonds contact with 2NH_3_ atoms, GLN189 residue along NH atom and GLU220 and ASP2218 residues with water molecules interact with 2O atoms. It has also formed two salt bridges among ASP2218 and GLU220 residues with 2NH_3_ atoms and two pi-pi stacking contacts among TYR457 and two benzene rings of the ligand (Fig 8Aa). Integrin alpha-v beta-8 (PDBID: 6UJB) in complex with Pixantrone Maleate involves 15 water molecules that form water bridges and hydrogen bonds among VAL280, VAL226, ASP24 and VAL23 residues with 2NH_3_ atoms, ILE344, SER278, and MET408 residues with 2NH atoms, LYS253 residue interacts with N atom and 2O atoms along SER279, SER407, residues and SER97, PHE21 residues with water molecules. Additionally, ASP162 residue formed two salt bridges with 2NH_3_ atoms and a pi-pi stacking contact along TYR406 with the benzene ring of the ligand (Fig 8Ba). The complex of TBK1(PDBID: 6NT9) and Pixantrone Maleate involves more than 15 water molecules, which act as a water bridge to provide complex stability. The residues GLU561, ASN557, SER268, GLU100, GLU109, LEU269, GLU375, SER430, and GLU376 residues connect with hydrogen bonds along 2NH_3_ atoms, THR431, and SER430 with hydrogen bonds along N atom, GLN553 along NH atom and 2O atoms with hydrogen bonds interact along GLN553 residue, and GLU550, GLU375, ALA321 residues with water molecules. It has formed 2 salt bridges among GLU561, GLU376, and GLU375 with 2NH_3_ atoms, 2 pi-cation contact LYS323 residue with two benzene rings, and HIS321 residue along pi-pi stacking with benzene ring of the Pixantrone Maleate ligand (Fig 8Ca). Further, the interaction count for each case is shown in Fig 8Ab–8Cb in a different colour to make it clear. These intricate interaction patterns revealed by the SID demonstrate these complex protein-ligand interactions and their stabilising nature, reinforcing Pixantrone Maleate’s potential as a robust multitargeted drug candidate for combating cervical cancer.

## 4. Discussion

The methods employed, including ligand library preparation, molecular docking studies, pharmacokinetics analysis, molecular interaction fingerprinting, and molecular dynamics simulations, have collectively provided valuable insights into the potential of Pixantrone Maleate as a multitargeted drug candidate for cervical cancer treatment. First and foremost, assessing the reliability and validity of the methods used throughout the study is crucial. The meticulous preparation of the ligand library from FDA-approved drugs, based on rigorous safety and efficacy evaluations, is a critical step that ensures the selection of compounds with well-documented pharmacological profiles and known mechanisms of action. This choice expedites drug development and reduces the time and costs of transitioning compounds into clinical trials. The hierarchical use of docking algorithms, such as High Throughput Virtual Screening (HTVS), Standard Precision, and Extra Precision (XP), followed by Molecular Mechanics-based Generalised Born Surface Area (MM/GBSA) analysis, further refines the selection of compounds based on binding free energy levels. This approach not only narrows down the pool of candidate molecules but also aids in identifying potential multitargeted drugs. The consistency of these findings across multiple proteins underscores the robustness of the molecular docking results. Assessing the pharmacokinetics properties of a drug candidate is a critical step in drug development, as it directly impacts its absorption, distribution, metabolism, and elimination within the body. The QikProp analysis of Pixantrone Maleate reveals favourable characteristics, including moderate acidity, multiple amine groups for potential hydrogen bonding, and low molecular weight, all aligning with drug-like qualities. The compound’s reasonable globularity and human oral absorption estimate enhance its attractiveness as a drug candidate, and compliance with the Rule of Five and Rule of Three signifies its potential as a lead compound for further development. These pharmacokinetic properties support Pixantrone Maleate’s suitability for oral administration and underscore its solubility, a crucial factor in effective drug delivery. The molecular interaction fingerprinting study provides a deeper understanding of Pixantrone Maleate’s interactions with key residues in the target proteins. The identification of specific amino acids, such as phenylalanine [35], valine [2], and serine (SER), forming stable interactions with the ligand highlights the compound’s ability to establish a network of stabilising forces within protein-ligand complexes. These interactions are vital in enhancing Pixantrone Maleate’s overall stability and potential efficacy as a drug candidate. The molecular interaction fingerprinting analysis reinforces the compound’s suitability as a multitargeted drug for cervical cancer by demonstrating its ability to form stable and specific interactions with essential residues. Molecular dynamics simulations are instrumental in gaining insights into the dynamic behaviour of molecules and atoms over time. The simulation of Pixantrone Maleate in complex with its target proteins, DNA polymerase epsilon subunit 2, Integrin alpha-v beta-8, and TBK1, using the Desmond package, provides valuable information about the stability, structural changes, and interactions of these complexes. The Root Mean Square Deviation (RMSD) analysis reveals that, despite initial fluctuations during system equilibration, the protein-ligand complexes reach stable states, indicating the reliability of the interactions. These fluctuations are likely attributed to the solute medium’s thermal energy and transient interactions. The Root Mean Square Fluctuation (RMSF) analysis further assesses the flexibility and rigidity of individual residues within the complexes. Although some residues exhibit fluctuations beyond 2 Å, they maintain stable interactions with Pixantrone Maleate. The complex interplay between dynamic behaviours and stable interactions highlights the intricate nature of these protein-ligand complexes. The Simulation Interaction Diagram (SID) visually represents these interactions, emphasising hydrogen bonds, van der Waals forces, electrostatic interactions, salt bridges, and pi-cation contacts, all contributing to complex stability. These findings collectively validate the potential of Pixantrone Maleate as a robust multitargeted drug candidate for cervical cancer.

Identifying Pixantrone Maleate as a multitargeted inhibitor with strong binding affinities for key proteins involved in cervical cancer opens new avenues for therapeutic development. Its favourable pharmacokinetic properties and adherence to drug development rules underscore its potential as a lead compound for further investigation. The stable interactions observed in molecular dynamics simulations reinforce its suitability as a drug candidate. These findings contribute to our understanding of cervical cancer and serve as a template for the rational design of novel therapeutics targeting multiple proteins. Furthermore, investigating potential synergistic effects with existing cervical cancer treatments should be a priority and combination therapies incorporating Pixantrone Maleate may enhance treatment outcomes and reduce the risk of drug resistance. To expedite the drug development process, virtual screening of additional compound libraries beyond FDA-approved drugs could identify novel candidates with even more significant potential. Employing advanced computational techniques, such as deep learning-based approaches for drug discovery, can aid in the identification of new lead compounds and optimise the selection of multitargeted drug candidates. The quantum mechanical calculations employing DFT with the B3LYP-D3 functional and a 6-31g** basis set provided valuable insights into its electronic structure, stability, and reactivity. The HOMO-LUMO energy gap indicates its potential for chemical reactivity, while polarizability hints at its responsiveness to external influences. Thermodynamic properties and vibrational frequencies offer critical data for understanding its behaviour under various conditions. Electrostatic potential and ALIE data reveal charge distribution and reactivity patterns. These findings are essential for tailoring Pixantrone Maleate’s application in drug design and optimising its pharmaceutical properties. Also, studying the mechanisms underlying Pixantrone Maleate’s interactions with target proteins at the atomic level can guide the design of analogues and derivatives with improved binding affinities and selectivities. In parallel, comprehensive toxicology and safety assessments are essential to ensure the suitability of Pixantrone Maleate for clinical trials. Addressing potential side effects and evaluating its safety profile in preclinical models will be critical before advancing, and exploring the potential of Pixantrone Maleate in personalised medicine approaches is worth considering and tailoring treatment regimens based on individual patient profiles and genetic variations may optimise therapeutic outcomes and reduce adverse effects.

## 5. Conclusion

Our comprehensive computational screening and validation study have identified Pixantrone Maleate as a promising multitargeted drug candidate for cervical cancer treatment. The reliability of the methods, strong binding affinities, favourable pharmacokinetic properties, stable interactions, and dynamic behaviour observed in simulations collectively support its potential. However, it is essential to move beyond computational predictions and embark on a rigorous journey of experimental validation, toxicological assessment, and clinical trials to bring this promising compound closer to benefiting cervical cancer patients.

## Supporting information

S1 Dataset The exported data of SID to understand the trajectory of each MDS are provided. (ZIP)
