# Supplementary material for: Delineating Pixantrone Maleate’s adroit activity against cervical cancer proteins through multitargeted docking-based MM\GBSA, QM-DFT and MD simulation
Source: PLoS One. 2023 Dec 15;18(12):e0295714. doi: 10.1371/journal.pone.0295714 (PMC10723688; doi:10.1371/journal.pone.0295714)
Supplement: S1 Dataset — (ZIP) [file pone.0295714.s001.zip › Data_3_6NT9/desmond_md_job_6NT9.pdf]

Simulation Interactions Diagram Report

Simulation Details

Jobname: desmond_md_job_6NT9
Entry title: 6NT9 - minimized S5059 Pixantrone Maleate.cdx

CPU #	Job Type	Ensemble	Temp. [K]	Sim. Time [ns]	# Atoms	# Waters	Charge
1	mdsim	NPT	300.0	100.102	110141	33135	0

Protein Information

Tot. Residues	Prot. Chain(s)	Res. in Chain(s)	# Atoms	# Heavy Atoms	Charge
660	'A'	ict_values([660]	10686	5337	-7

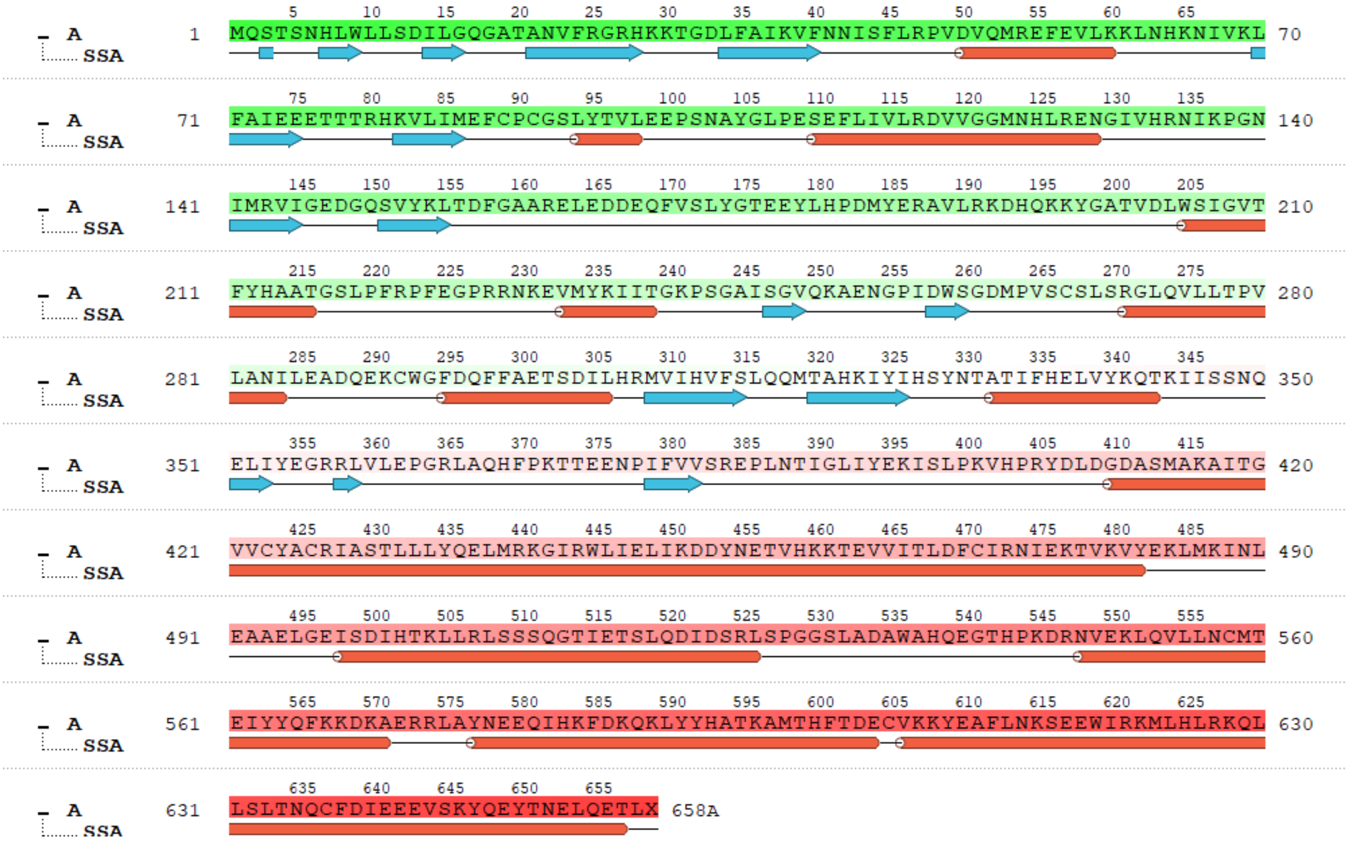

Ligand Information

SMILES	[NH3+]CCNc1ccc(NCC[NH3+])c(c12)C(=O)c3c(C2=O)ccnc3
PDB Name	'UNK'
Num. of Atoms	45 (total) 24 (heavy)
Atomic Mass	327.389 au
Charge	+2
Mol. Formula	C17H21N5O2
Num. of Fragments	3
Num. of Rot. Bonds	6

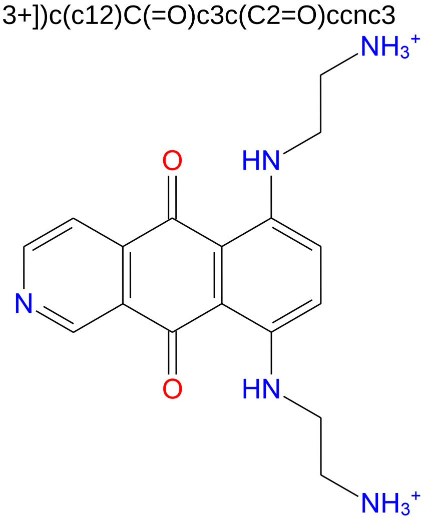

Counter Ion/Salt Information

Type	Num.	Concentration [mM]	Total Charge
Na	5	2.744	+5

Protein-Ligand RMSD

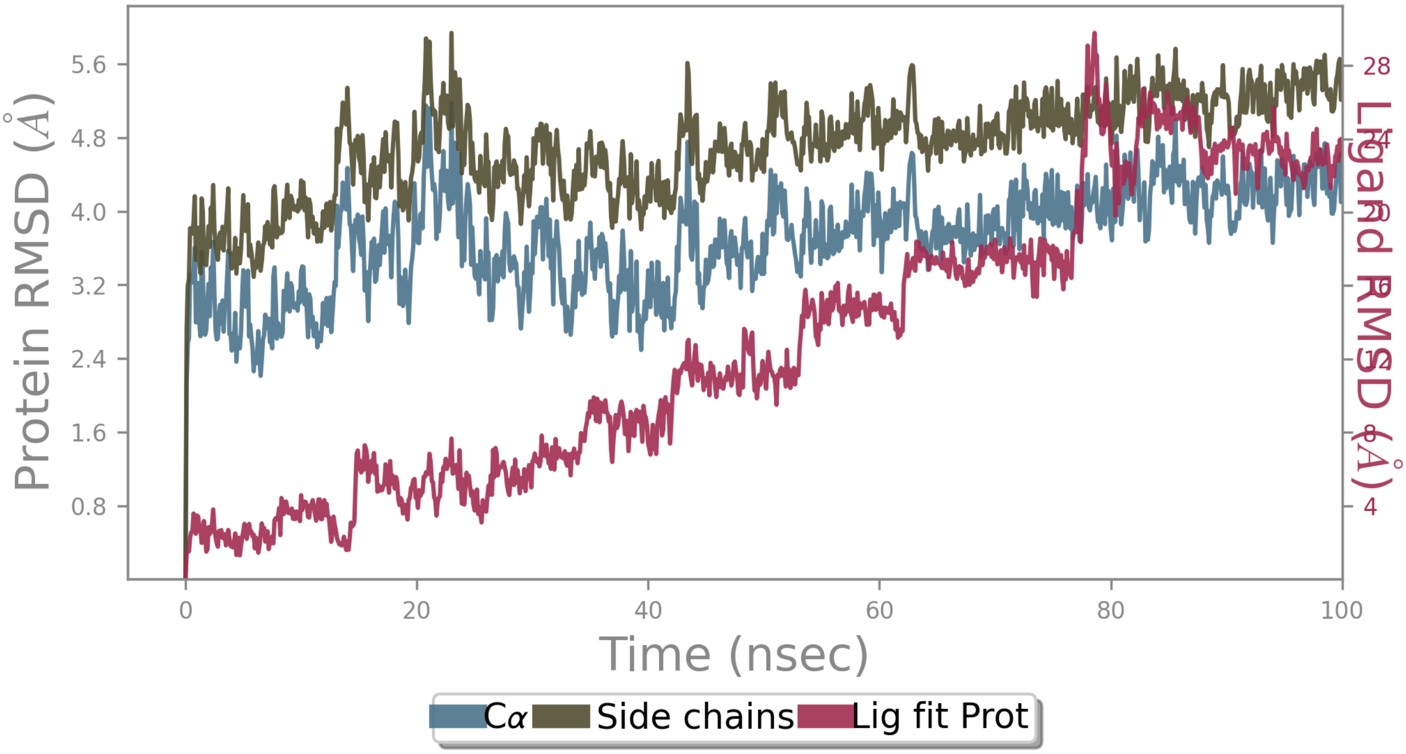

The Root Mean Square Deviation (RMSD) is used to measure the average change in displacement of a selection of atoms for a particular frame with respect to a reference frame. It is calculated for all frames in the trajectory. The RMSD for frame *x* is:

$$RMSD_x = \sqrt{\frac{1}{N}\sum_{i=1}^{N}(r_i'(t_x)) - r_i(t_{ref}))^2}$$

where *N* is the number of atoms in the atom selection; t_{ref} is the reference time, (typically the first frame is used as the reference and it is regarded as time *t*=0); and *r'* is the position of the selected atoms in frame *x* after superimposing on the reference frame, where frame *x* is recorded at time t_x. The procedure is repeated for every frame in the simulation trajectory.

<u>Protein RMSD</u>: The above plot shows the RMSD evolution of a protein (left *Y*-axis). All protein frames are first aligned on the reference frame backbone, and then the RMSD is calculated based on the atom selection. Monitoring the RMSD of the protein can give insights into its structural conformation throughout the simulation. RMSD analysis can indicate if the simulation has equilibrated — its fluctuations towards the end of the simulation are around some thermal average structure. Changes of the order of 1-3 Å are perfectly acceptable for small, globular proteins. Changes much larger than that, however, indicate that the protein is undergoing a large conformational change during the simulation. It is also important that your simulation converges — the RMSD values stabilize around a fixed value. If the RMSD of the protein is still increasing or decreasing on average at the end of the simulation, then your system has not equilibrated, and your simulation may not be long enough for rigorous analysis.

<u>Ligand RMSD</u>: Ligand RMSD (right *Y*-axis) indicates how stable the ligand is with respect to the protein and its binding pocket. In the above plot, 'Lig fit Prot' shows the RMSD of a ligand when the protein-ligand complex is first aligned on the protein backbone of the reference and then the RMSD of the ligand heavy atoms is measured. If the values observed are significantly larger than the RMSD of the protein, then it is likely that the ligand has diffused away from its initial binding site.

Protein RMSF

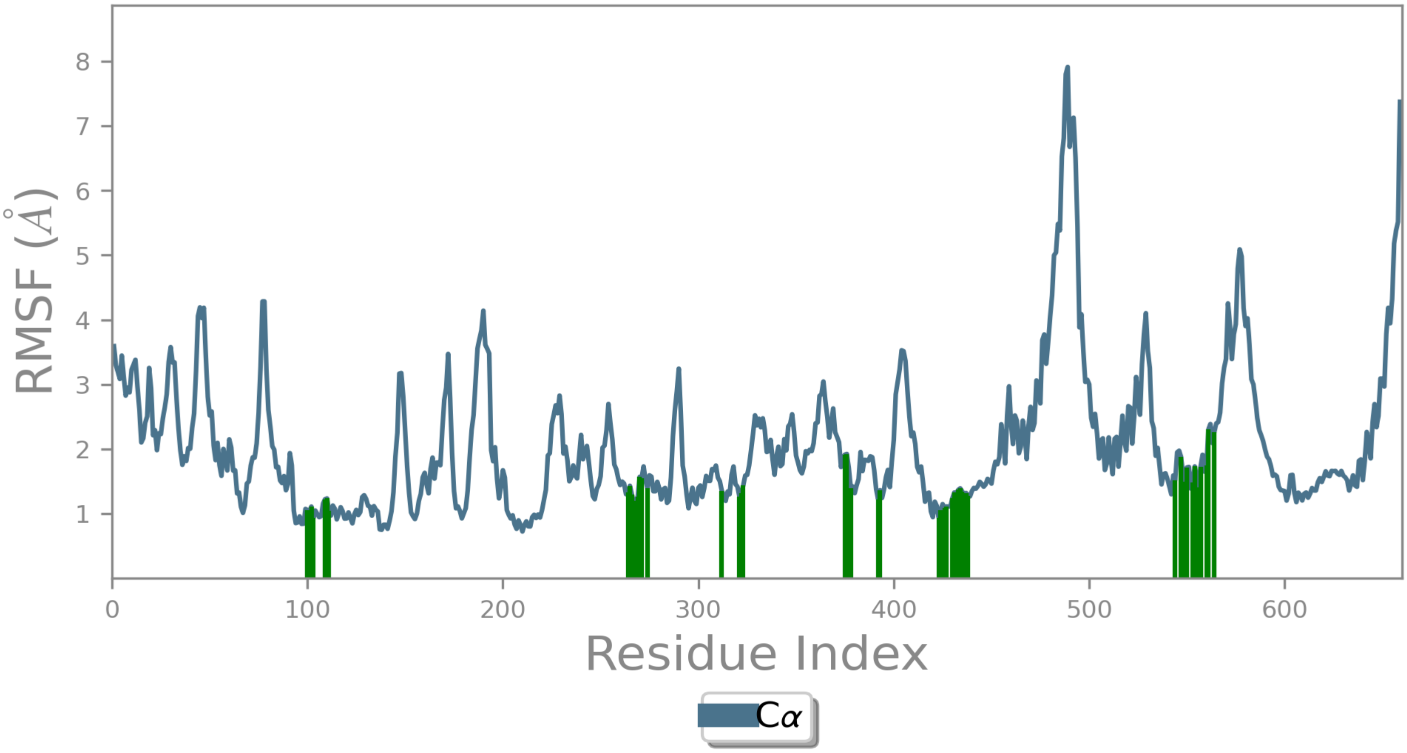

The Root Mean Square Fluctuation (RMSF) is useful for characterizing local changes along the protein chain. The RMSF for residue *i* is:

$$RMSF_i = \sqrt{\frac{1}{T}\sum_{t=1}^{T} < (r'_i(t)) - r_i(t_{ref}))^2 >}$$

where T is the trajectory time over which the RMSF is calculated, t_{ref} is the reference time, r_i is the position of residue *i*; *r'* is the position of atoms in residue *i* after superposition on the reference, and the angle brackets indicate that the average of the square distance is taken over the selection of atoms in the residue.

On this plot, peaks indicate areas of the protein that fluctuate the most during the simulation. Typically you will observe that the tails (*N*- and *C*-terminal) fluctuate more than any other part of the protein. Secondary structure elements like alpha helices and beta strands are usually more rigid than the unstructured part of the protein, and thus fluctuate less than the loop regions.

Ligand Contacts: Protein residues that interact with the ligand are marked with green-colored vertical bars.

Protein Secondary Structure

% Helix	% Strand	% Total SSE
40.08	10.94	51.02

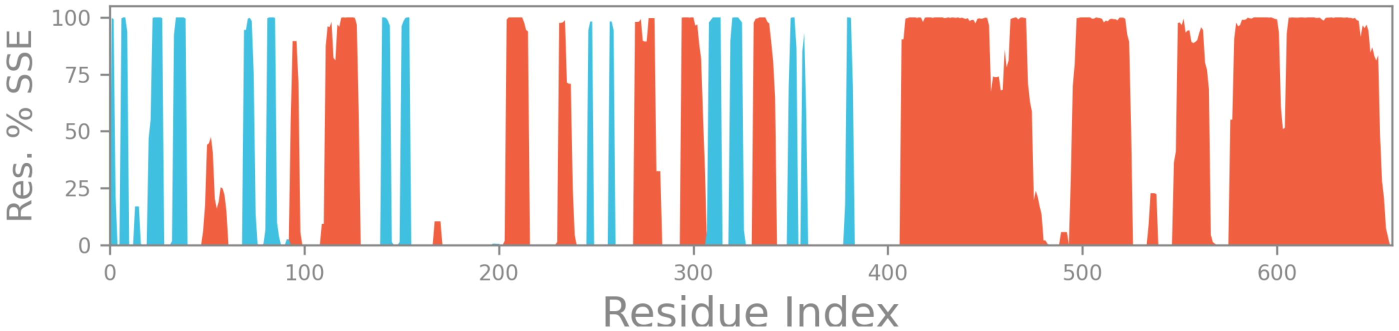

Protein secondary structure elements (SSE) like alpha-helices and beta-strands are monitored throughout the simulation. The plot above reports SSE distribution by residue index throughout the protein structure. The plot below summarizes the SSE composition for each trajectory frame over the course of the simulation, and the plot at the bottom monitors each residue and its SSE assignment over time.

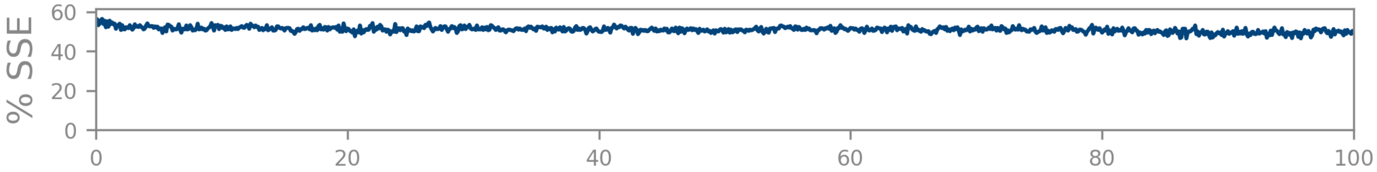

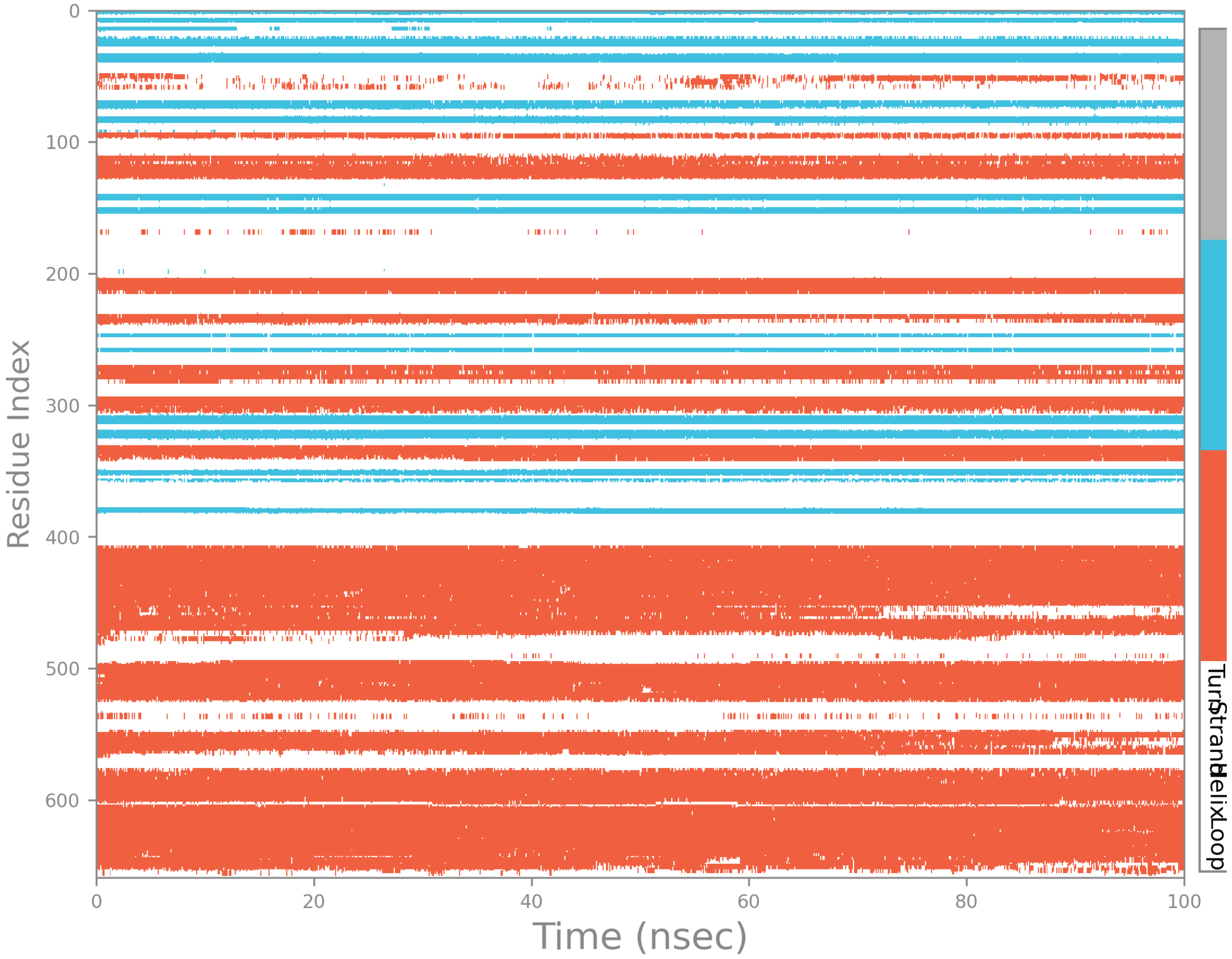

Ligand RMSF

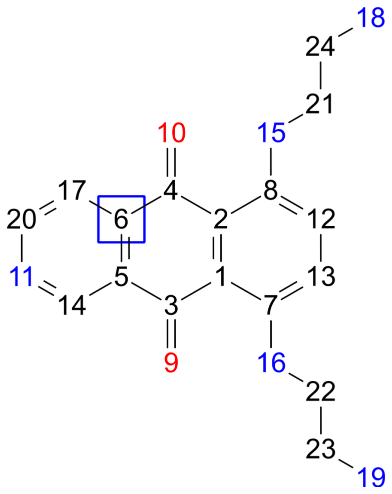

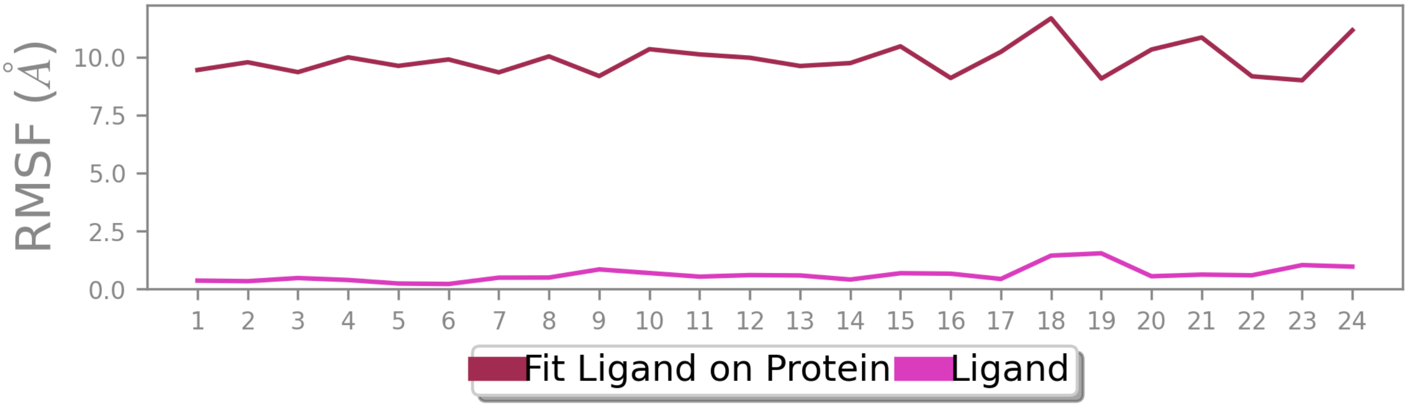

The Ligand Root Mean Square Fluctuation (L-RMSF) is useful for characterizing changes in the ligand atom positions. The RMSF for atom *i* is:

$$RMSF_i = \sqrt{\frac{1}{T}\sum_{t=1}^{T}(r_i'(t)) - r_i(t_{ref}))^2}$$

where *T* is the trajectory time over which the RMSF is calculated, t_{ref} is the reference time (usually for the first frame, and is regarded as the *zero* of time); *r* is the position of atom *i* in the reference at time t_{ref}, and *r'* is the position of atom *i* at time *t* after superposition on the reference frame.

Ligand RMSF shows the ligand's fluctuations broken down by atom, corresponding to the 2D structure in the top panel. The ligand RMSF may give you insights on how ligand fragments interact with the protein and their entropic role in the binding event. In the bottom panel, the 'Fit Ligand on Protein' line shows the ligand fluctuations, with respect to the protein. The protein-ligand complex is first aligned on the protein backbone and then the ligand RMSF is measured on the ligand heavy atoms. 'Ligand' line shows fluctuations where the ligand in each frame is aligned on the ligand in the reference frame, and its fluctuations are measured for the ligand heavy atoms. These RMSF values reflect the internal atom fluctuations of the ligand.

Protein-Ligand Contacts

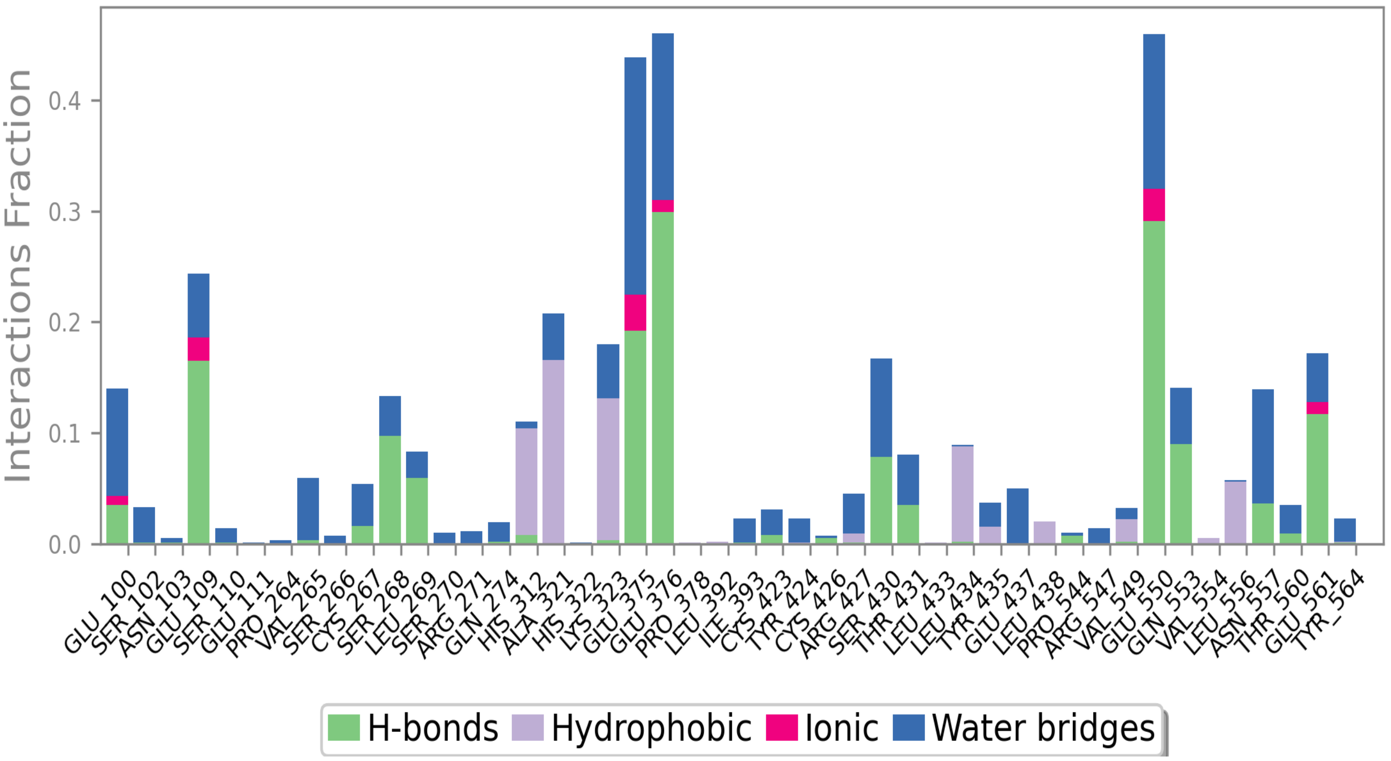

Protein interactions with the ligand can be monitored throughout the simulation. These interactions can be categorized by type and summarized, as shown in the plot above. Protein-ligand interactions (or 'contacts') are categorized into four types: Hydrogen Bonds, Hydrophobic, Ionic and Water Bridges. Each interaction type contains more specific subtypes, which can be explored through the 'Simulation Interactions Diagram' panel. The stacked bar charts are normalized over the course of the trajectory: for example, a value of 0.7 suggests that 70% of the simulation time the specific interaction is maintained. Values over 1.0 are possible as some protein residue may make multiple contacts of same subtype with the ligand.

<u>Hydrogen Bonds</u>: (H-bonds) play a significant role in ligand binding. Consideration of hydrogen-bonding properties in drug design is important because of their strong influence on drug specificity, metabolization and adsorption. Hydrogen bonds between a protein and a ligand can be further broken down into four subtypes: backbone acceptor; backbone donor; side-chain acceptor; side-chain donor.
The current geometric criteria for protein-ligand H-bond is: distance of 2.5 Å between the donor and acceptor atoms (D—H···A); a donor angle of ≥120° between the donor-hydrogen-acceptor atoms (D—H···A); and an acceptor angle of ≥90° between the hydrogen-acceptor-bonded_atom atoms (H···A—X).

<u>Hydrophobic contacts</u>: fall into three subtypes: π-Cation; π-π; and Other, non-specific interactions. Generally these type of interactions involve a hydrophobic amino acid and an aromatic or aliphatic group on the ligand, but we have extended this category to also include π-Cation interactions.
The current geometric criteria for hydrophobic interactions is as follows: π-Cation — Aromatic and charged groups within 4.5 Å; π-π — Two aromatic groups stacked face-to-face or face-to-edge; Other — A non-specific hydrophobic sidechain within 3.6 Å of a ligand's aromatic or aliphatic carbons.

<u>Ionic interactions</u>: or polar interactions, are between two oppositely charged atoms that are within 3.7 Å of each other and do not involve a hydrogen bond. We also monitor Protein-Metal-Ligand interactions, which are defined by a metal ion coordinated within 3.4 Å of protein's and ligand's heavy atoms (except carbon). All ionic interactions are broken down into two subtypes: those mediated by a protein backbone or side chains.

<u>Water Bridges</u>: are hydrogen-bonded protein-ligand interactions mediated by a water molecule. The hydrogen-bond geometry is slightly relaxed from the standard H-bond definition.
The current geometric criteria for a protein-water or water-ligand H-bond are: a distance of 2.8 Å between the donor and acceptor atoms (D—H···A); a donor angle of ≥110° between the donor-hydrogen-acceptor atoms (D—H···A); and an acceptor angle of ≥90° between the hydrogen-acceptor-bonded_atom atoms (H···A—X).

Protein-Ligand Contacts (cont.)

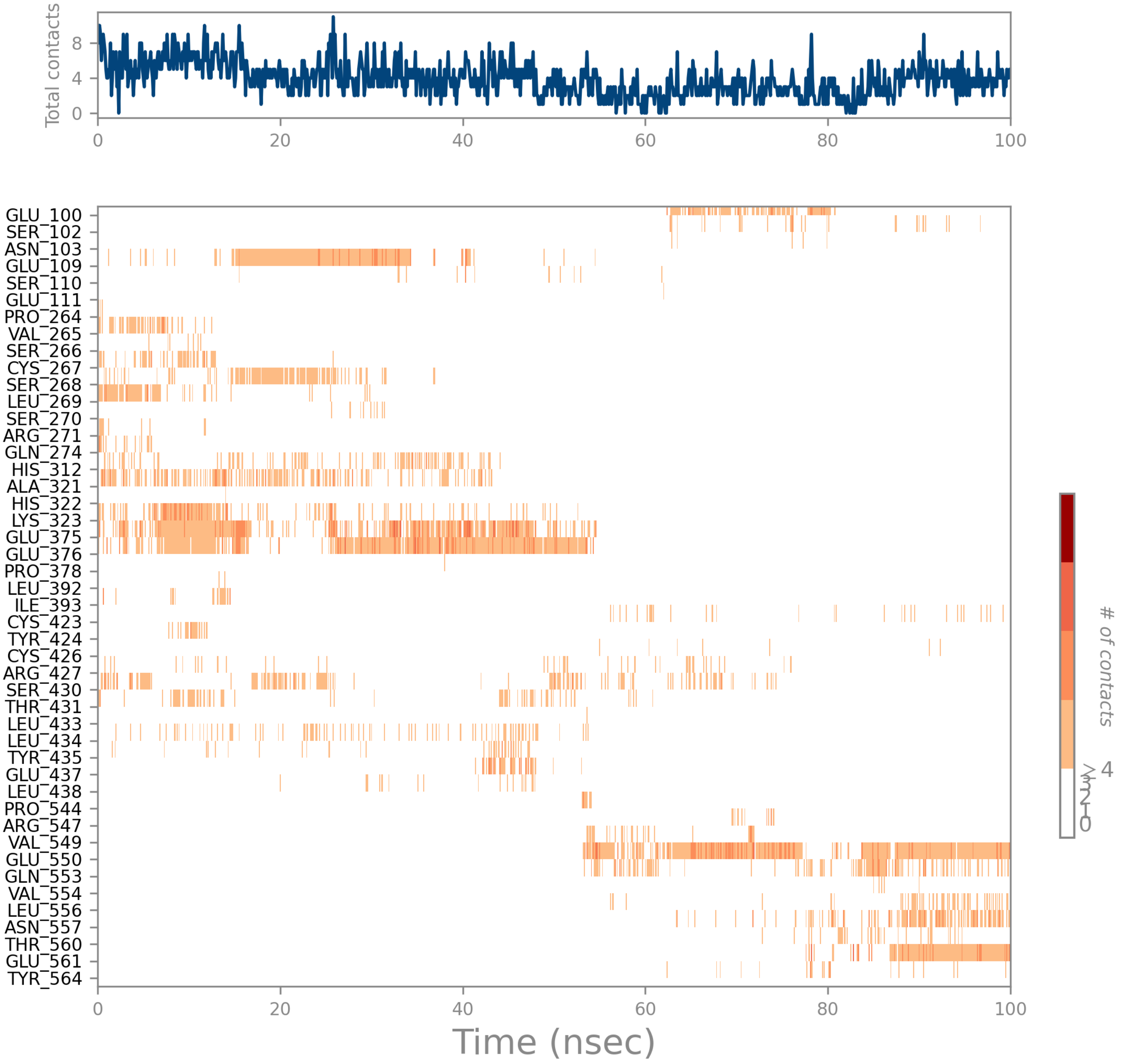

A timeline representation of the interactions and contacts (**H-bonds**, **Hydrophobic**, **Ionic**, **Water bridges**) summarized in the previous page. The top panel shows the total number of specific contacts the protein makes with the ligand over the course of the trajectory. The bottom panel shows which residues interact with the ligand in each trajectory frame. Some residues make more than one specific contact with the ligand, which is represented by a darker shade of orange, according to the scale to the right of the plot.

Ligand-Protein Contacts

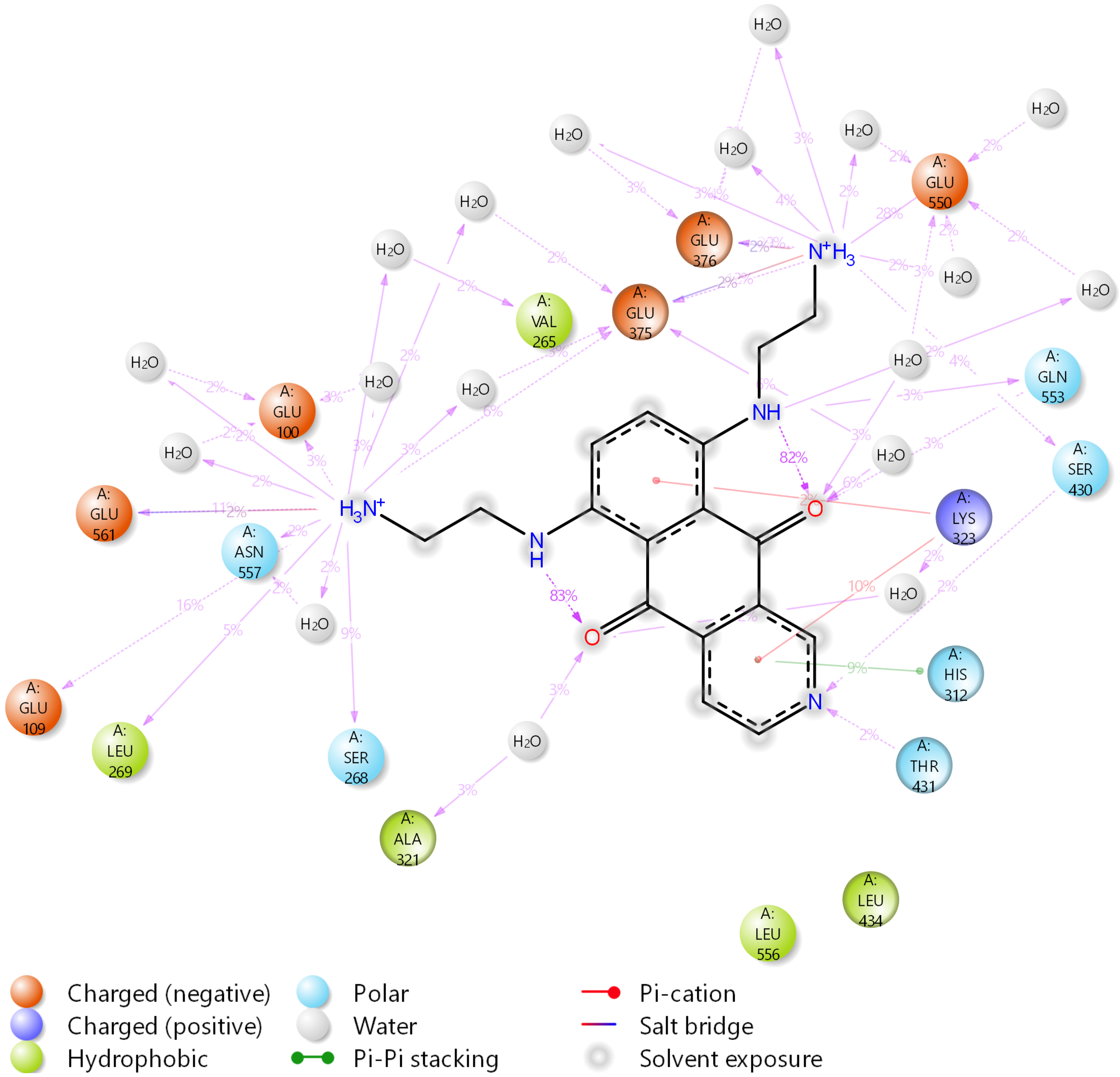

A schematic of detailed ligand atom interactions with the protein residues. Interactions that occur more than **2.0%** of the simulation time in the selected trajectory (0.00 through 100.00 nsec), are shown.
Note: it is possible to have interactions with >100% as some residues may have multiple interactions of a single type with the same ligand atom. For example, the ARG side chain has four H-bond donors that can all hydrogen-bond to a single H-bond acceptor.

Ligand Torsion Profile

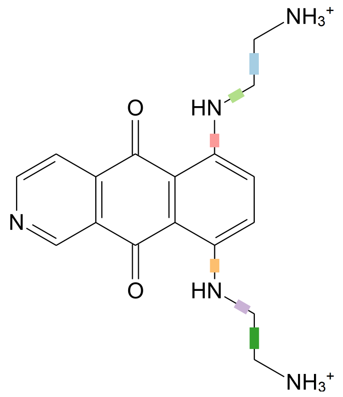

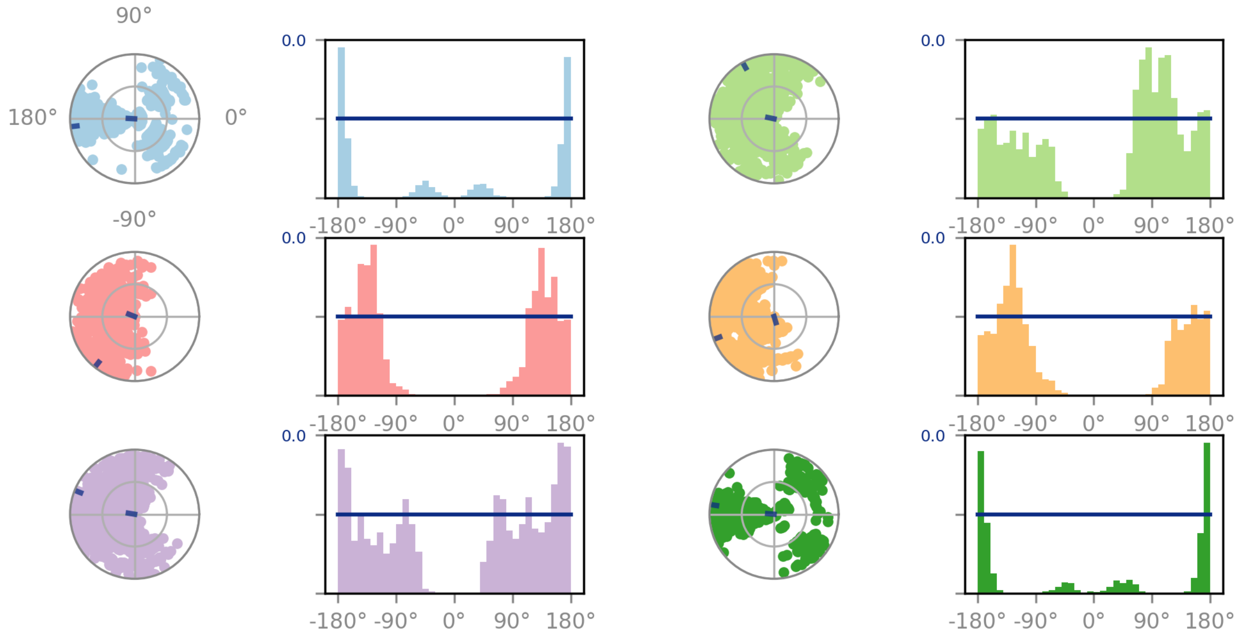

The ligand torsions plot summarizes the conformational evolution of every rotatable bond (RB) in the ligand throughout the simulation trajectory (0.00 through 100.00 nsec). The top panel shows the 2d schematic of a ligand with color-coded rotatable bonds. Each rotatable bond torsion is accompanied by a dial plot and bar plots of the same color.

Dial (or radial) plots describe the conformation of the torsion throughout the course of the simulation. The beginning of the simulation is in the center of the radial plot and the time evolution is plotted radially outwards.

The bar plots summarize the data on the dial plots, by showing the probability density of the torsion. If torsional potential information is available, the plot also shows the potential of the rotatable bond (by summing the potential of the related torsions). The values of the potential are on the left *Y*-axis of the chart, and are expressed in *kcal/mol*. Looking at the histogram and torsion potential relationships may give insights into the conformational strain the ligand undergoes to maintain a protein-bound conformation.

Ligand Properties

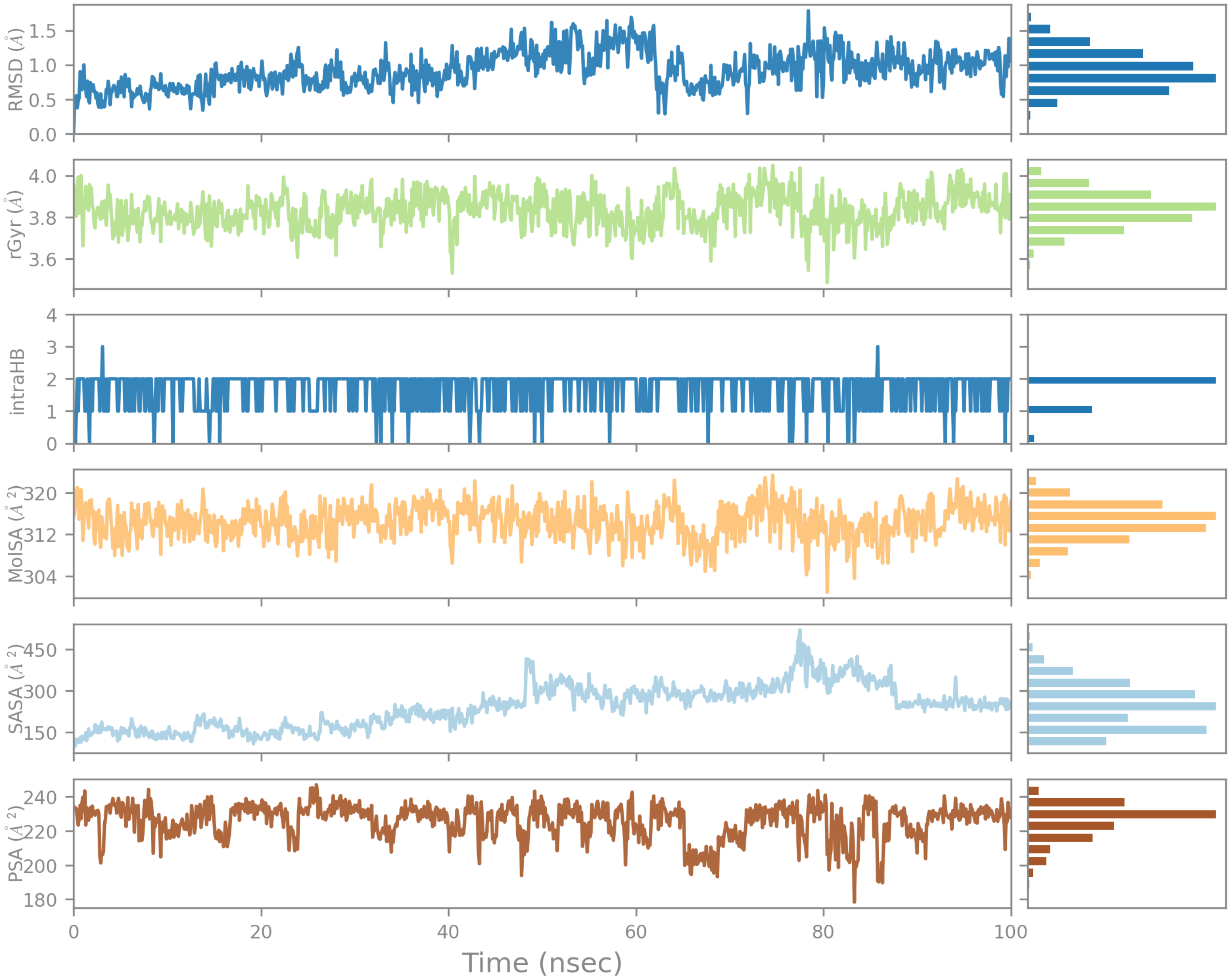

Ligand RMSD: Root mean square deviation of a ligand with respect to the reference conformation (typically the first frame is used as the reference and it is regarded as time *t*=0).

Radius of Gyration (rGyr): Measures the 'extendedness' of a ligand, and is equivalent to its principal moment of inertia.

Intramolecular Hydrogen Bonds (intraHB): Number of internal hydrogen bonds (HB) within a ligand molecule.

Molecular Surface Area (MolSA): Molecular surface calculation with 1.4 Å probe radius. This value is equivalent to a van der Waals surface area.

Solvent Accessible Surface Area (SASA): Surface area of a molecule accessible by a water molecule.

Polar Surface Area (PSA): Solvent accessible surface area in a molecule contributed only by oxygen and nitrogen atoms.
