# Supplementary figures and images for: Delineating Pixantrone Maleate’s adroit activity against cervical cancer proteins through multitargeted docking-based MM\GBSA, QM-DFT and MD simulation
Source: PLoS One. 2023 Dec 15;18(12):e0295714. doi: 10.1371/journal.pone.0295714 (PMC10723688; doi:10.1371/journal.pone.0295714)

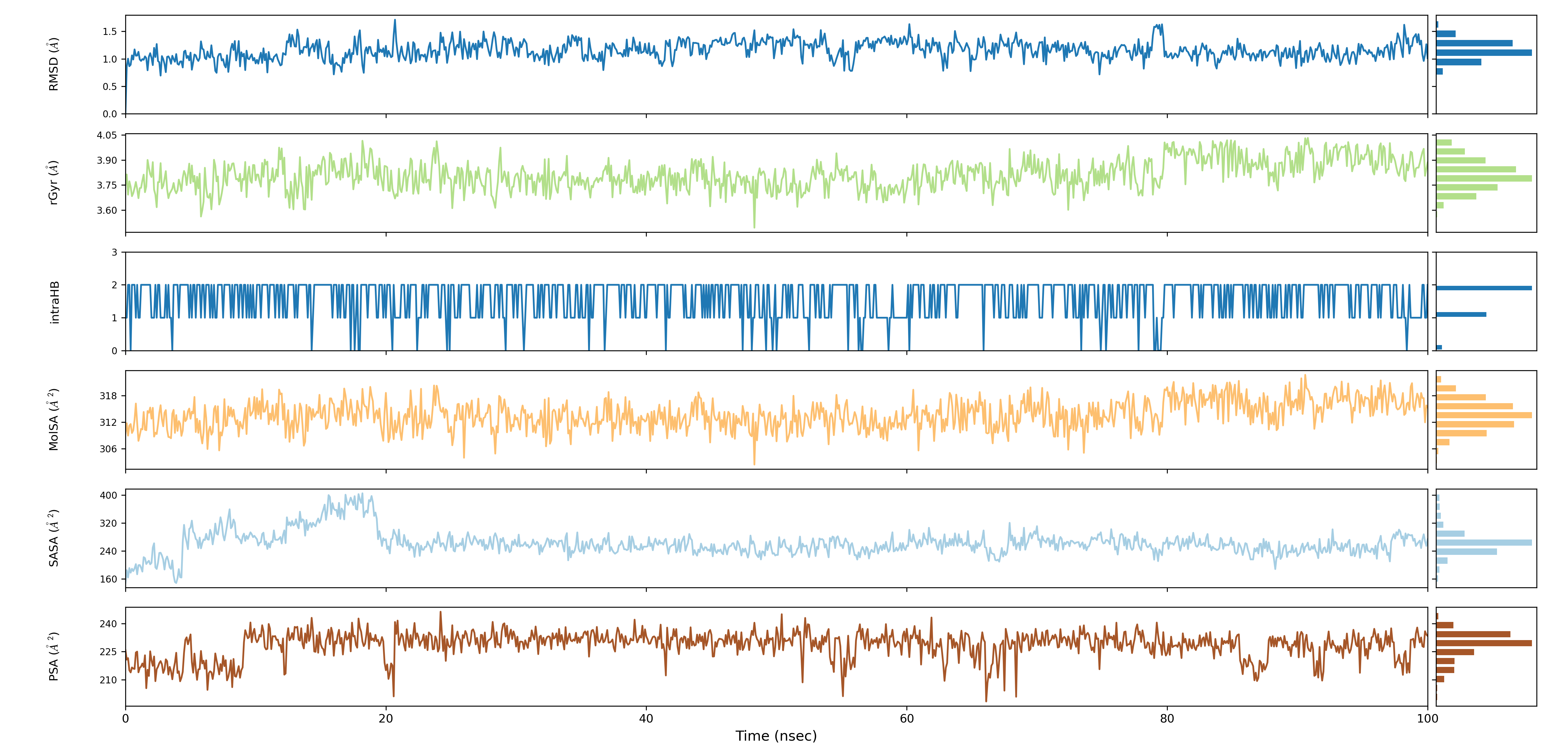

Supplement: S1 Dataset — (ZIP) [file pone.0295714.s001.zip › Data_1_5VBN/images/L-Properties.png]

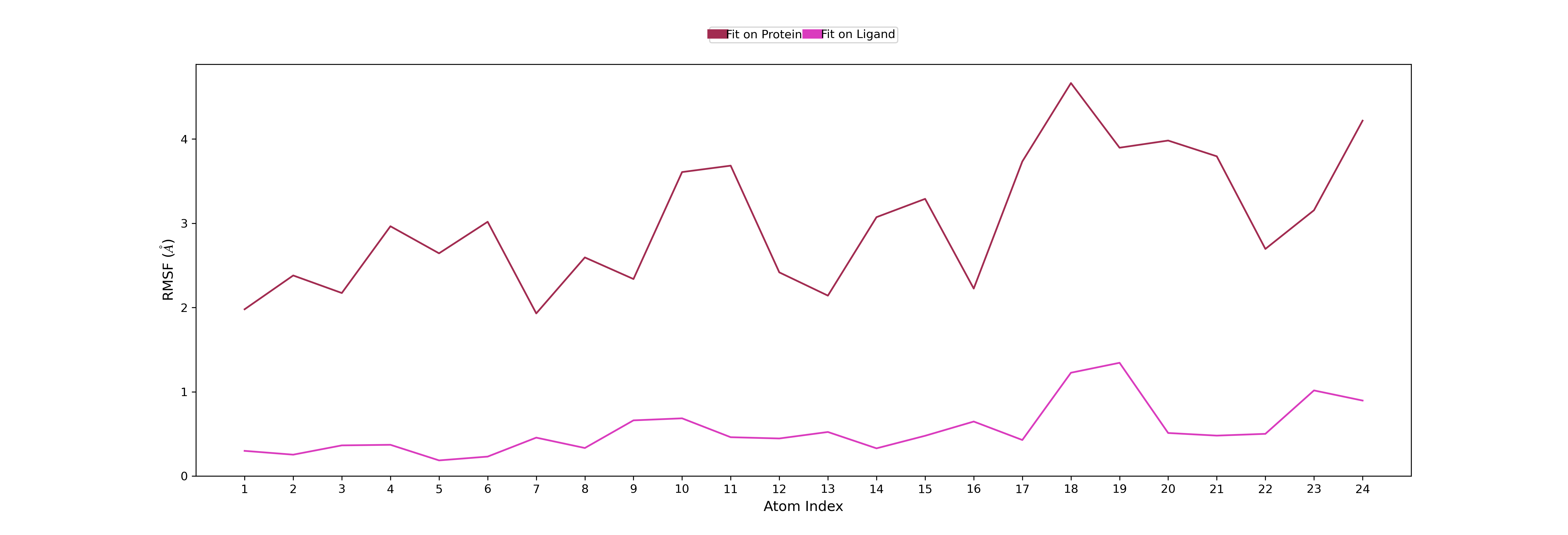

Supplement: S1 Dataset — (ZIP) [file pone.0295714.s001.zip › Data_1_5VBN/images/L-RMSF.png]

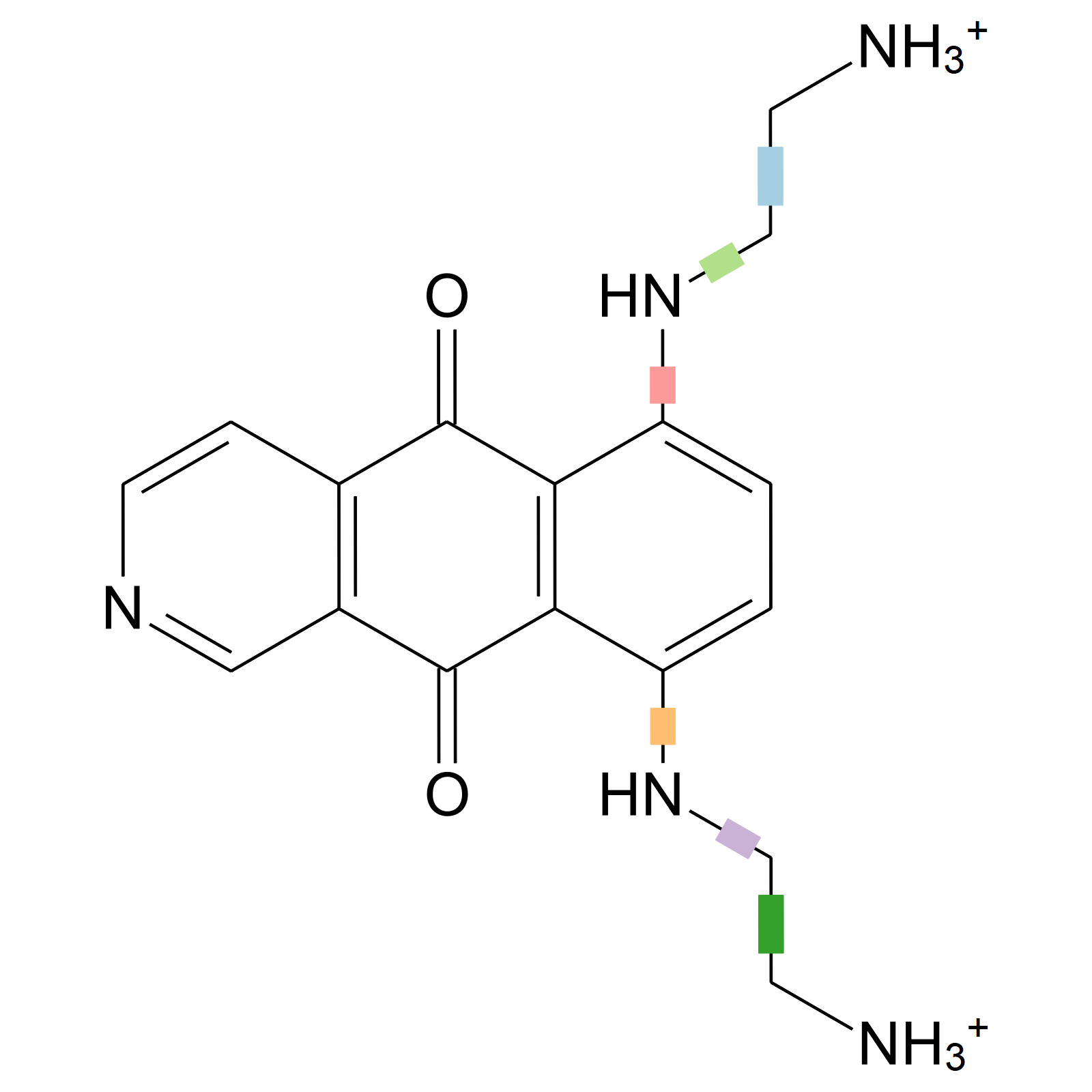

Supplement: S1 Dataset — (ZIP) [file pone.0295714.s001.zip › Data_1_5VBN/images/L-Torsions-2d.png]

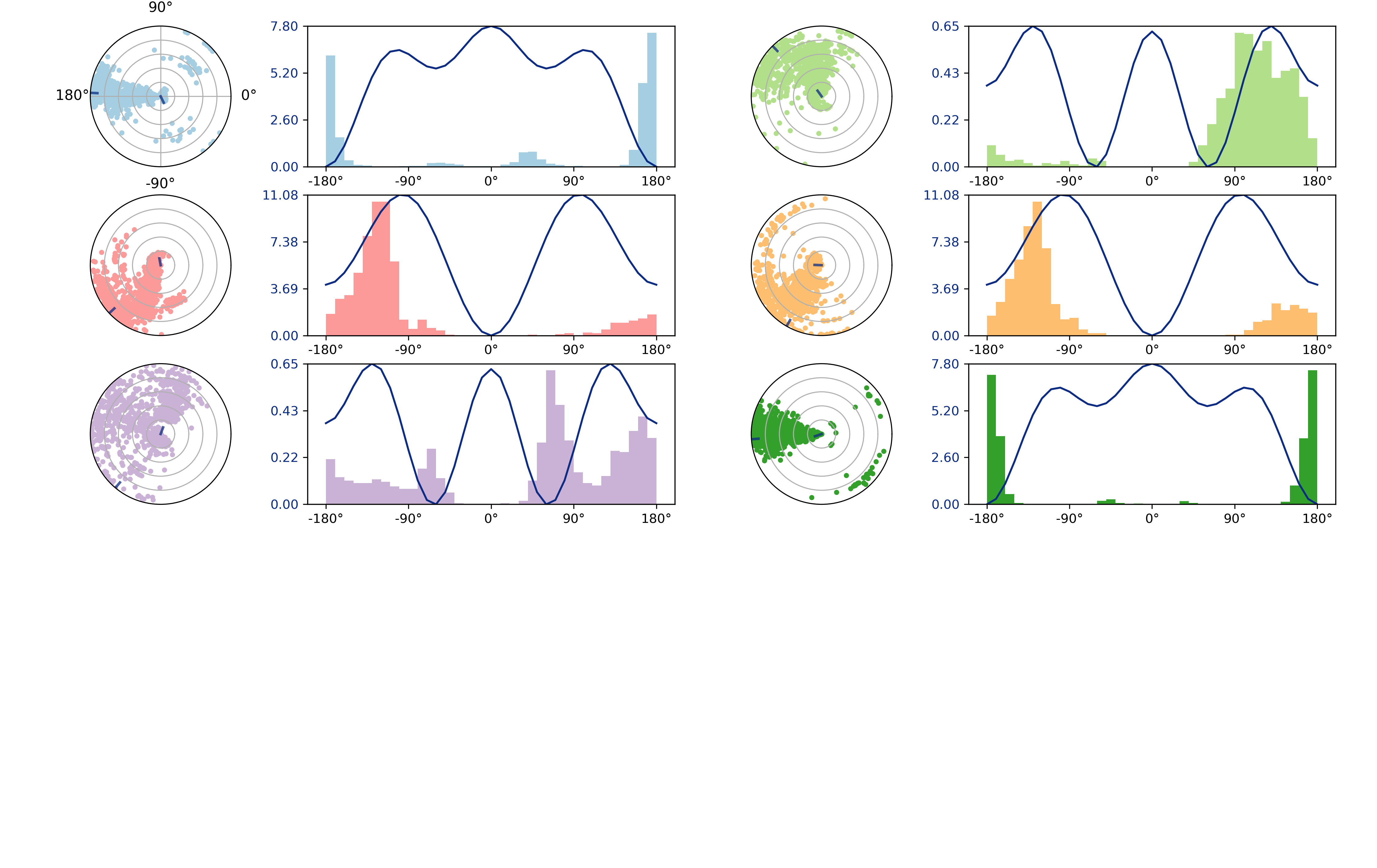

Supplement: S1 Dataset — (ZIP) [file pone.0295714.s001.zip › Data_1_5VBN/images/L-Torsions.png]

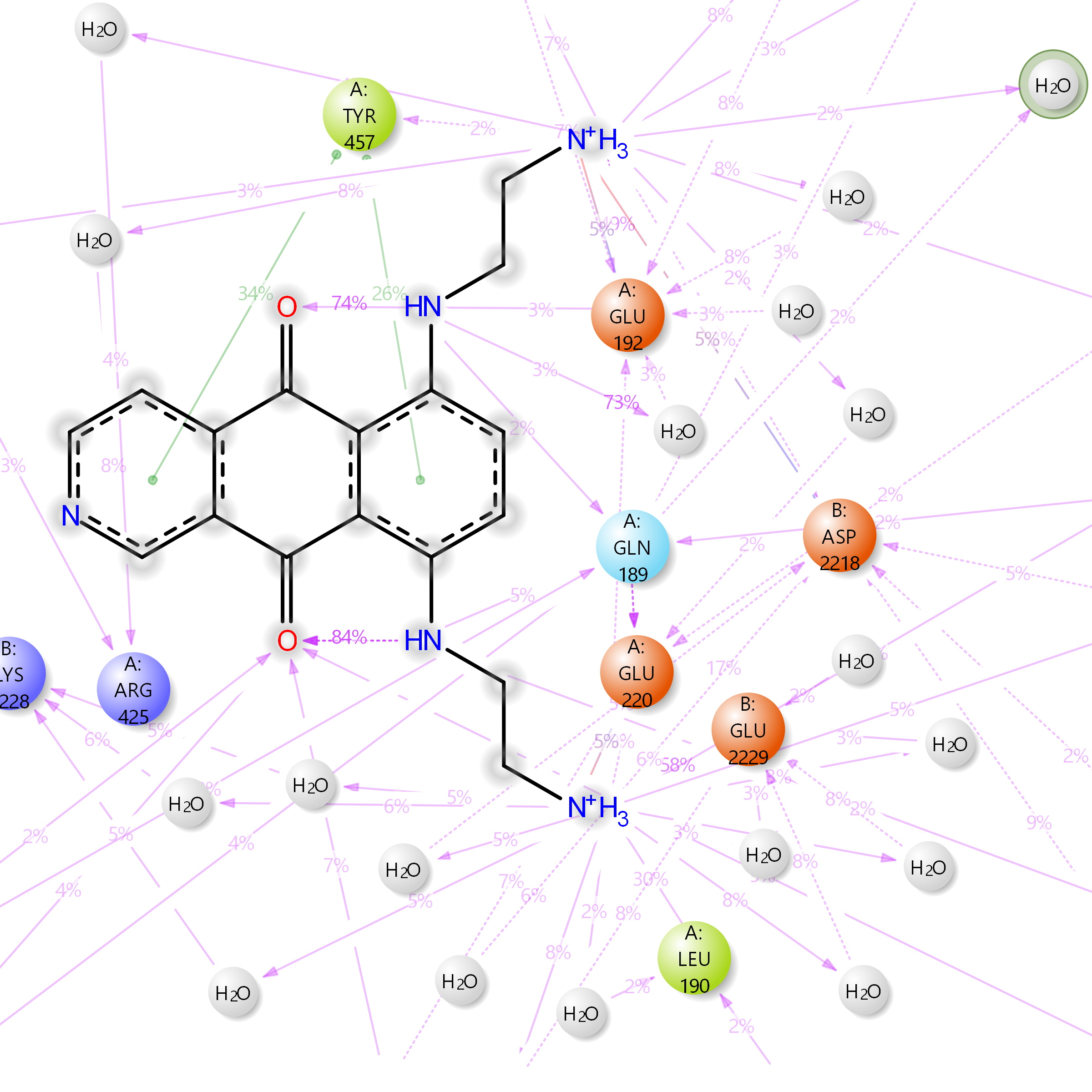

Supplement: S1 Dataset — (ZIP) [file pone.0295714.s001.zip › Data_1_5VBN/images/LP-Contacts_2d-Summary.png]

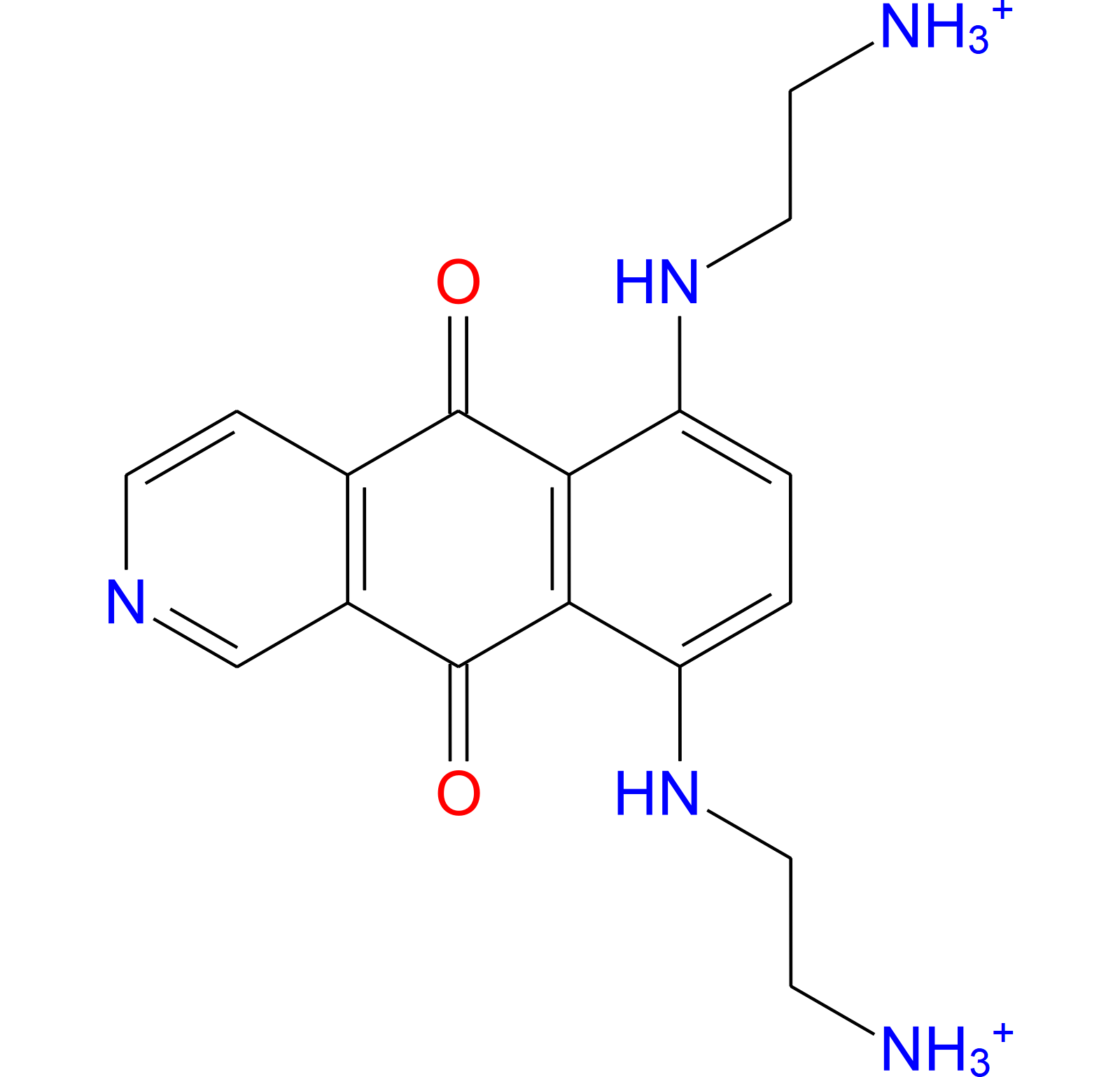

Supplement: S1 Dataset — (ZIP) [file pone.0295714.s001.zip › Data_1_5VBN/images/L_2d_main.png]

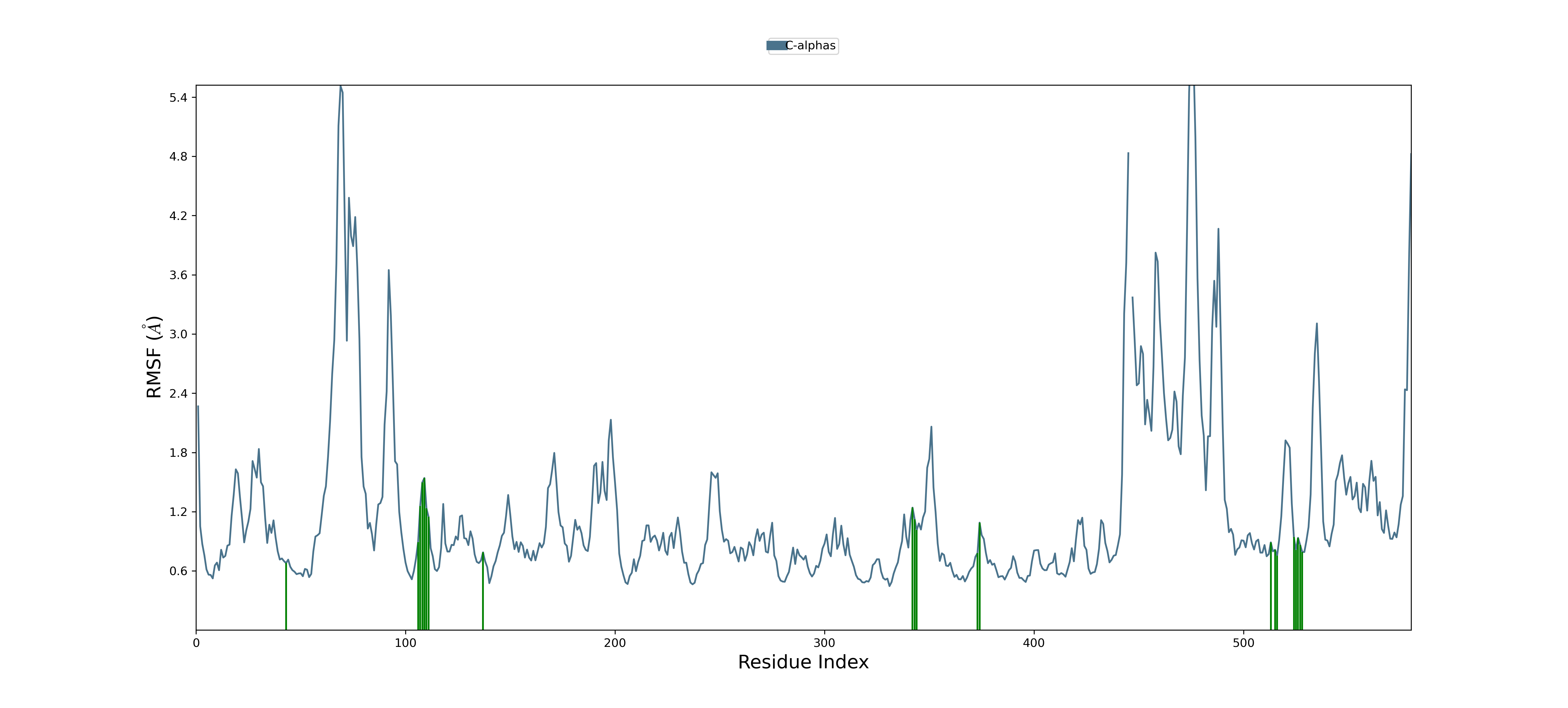

Supplement: S1 Dataset — (ZIP) [file pone.0295714.s001.zip › Data_1_5VBN/images/P-RMSF.png]

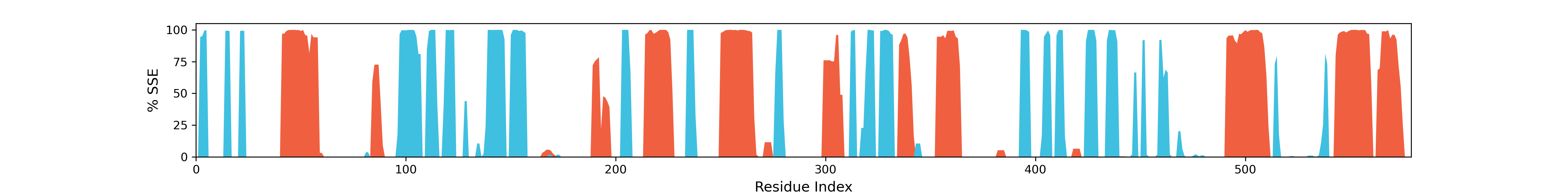

Supplement: S1 Dataset — (ZIP) [file pone.0295714.s001.zip › Data_1_5VBN/images/P-SSE_Histogram.png]

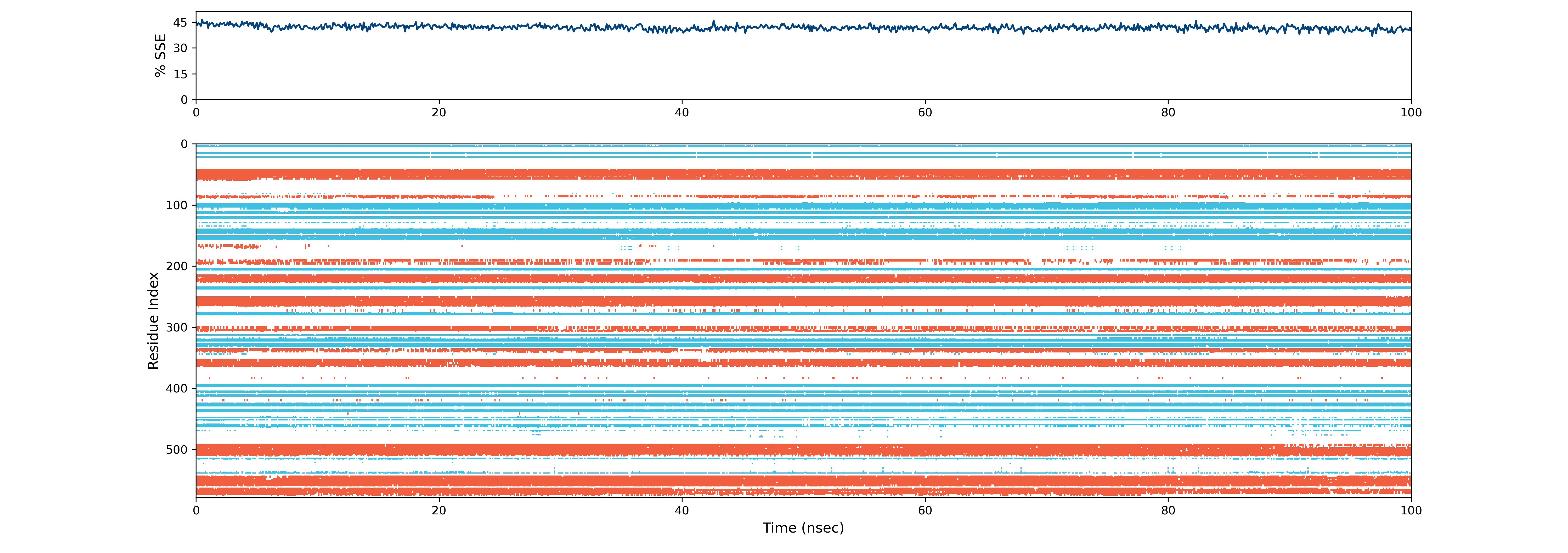

Supplement: S1 Dataset — (ZIP) [file pone.0295714.s001.zip › Data_1_5VBN/images/P-SSE_Timeline.png]

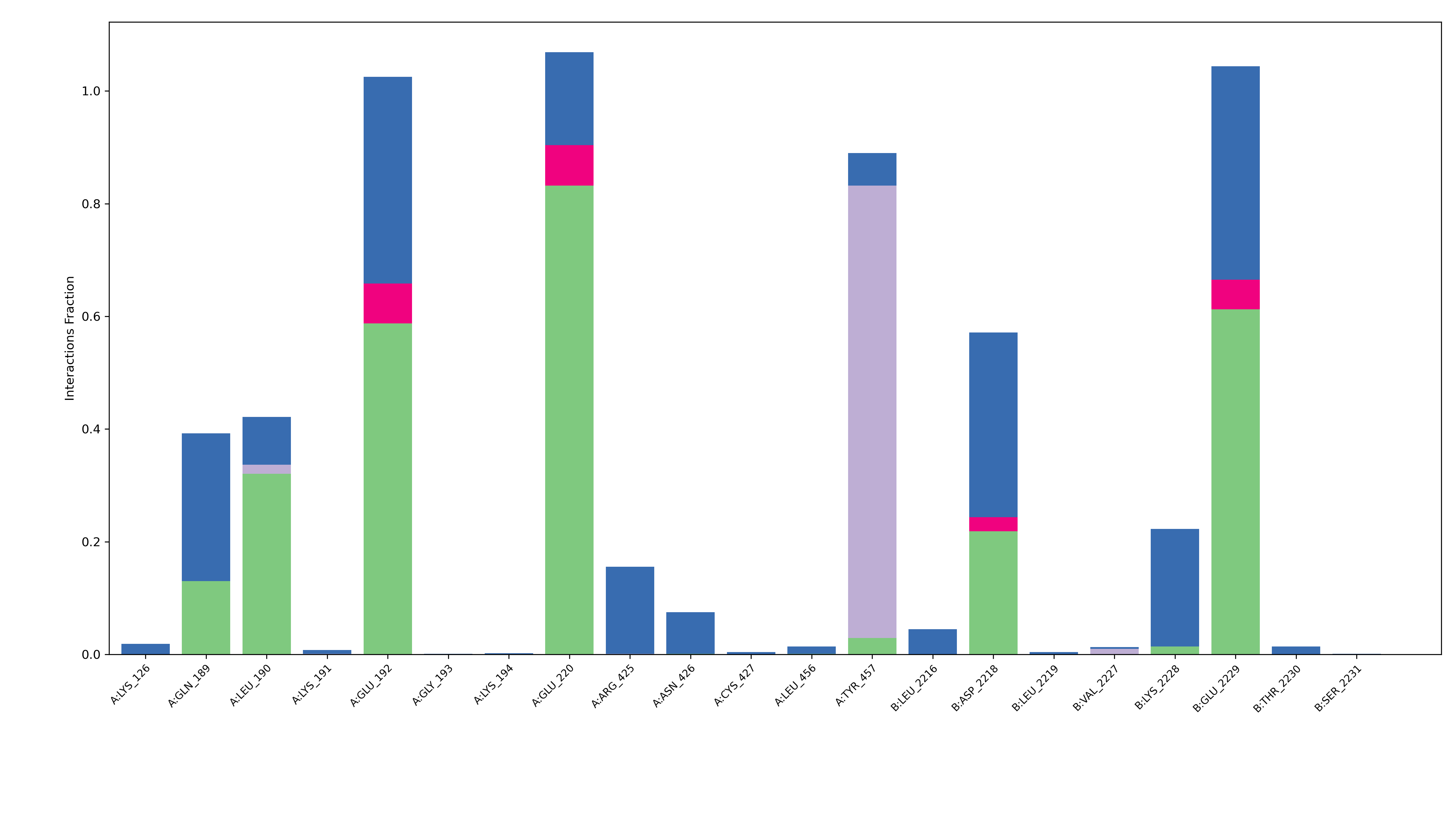

Supplement: S1 Dataset — (ZIP) [file pone.0295714.s001.zip › Data_1_5VBN/images/PL-Contacts_Histogram.png]

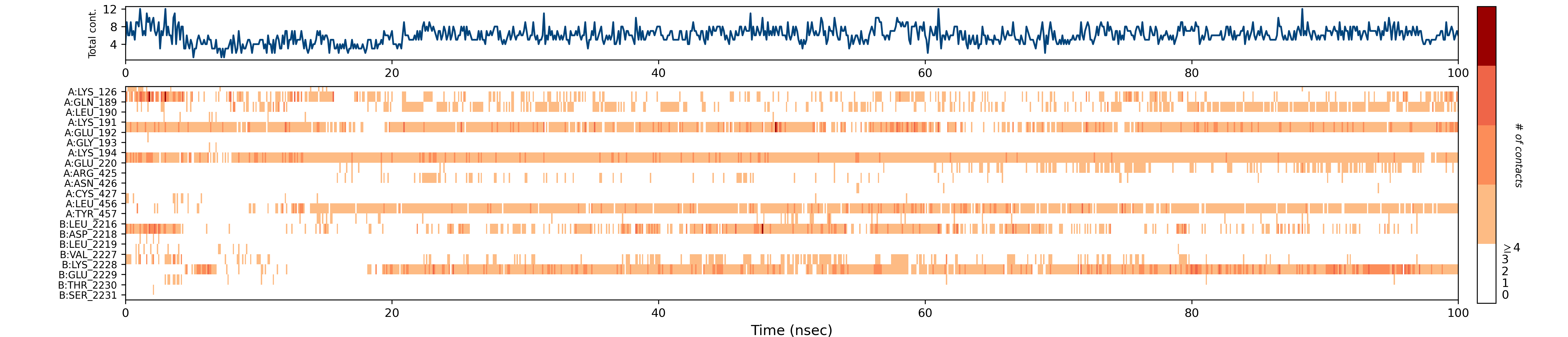

Supplement: S1 Dataset — (ZIP) [file pone.0295714.s001.zip › Data_1_5VBN/images/PL-Contacts_Timeline.png]

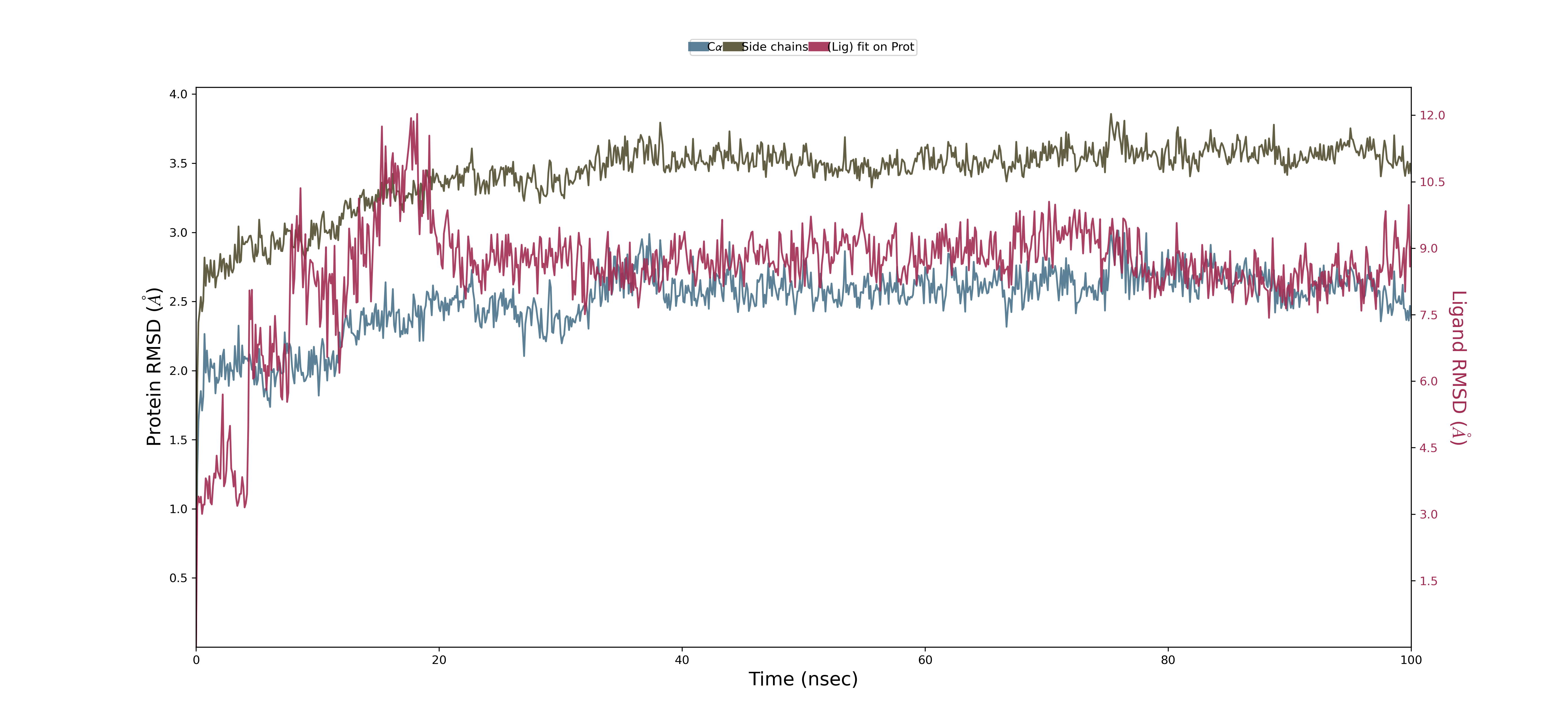

Supplement: S1 Dataset — (ZIP) [file pone.0295714.s001.zip › Data_1_5VBN/images/PL-RMSD.png]

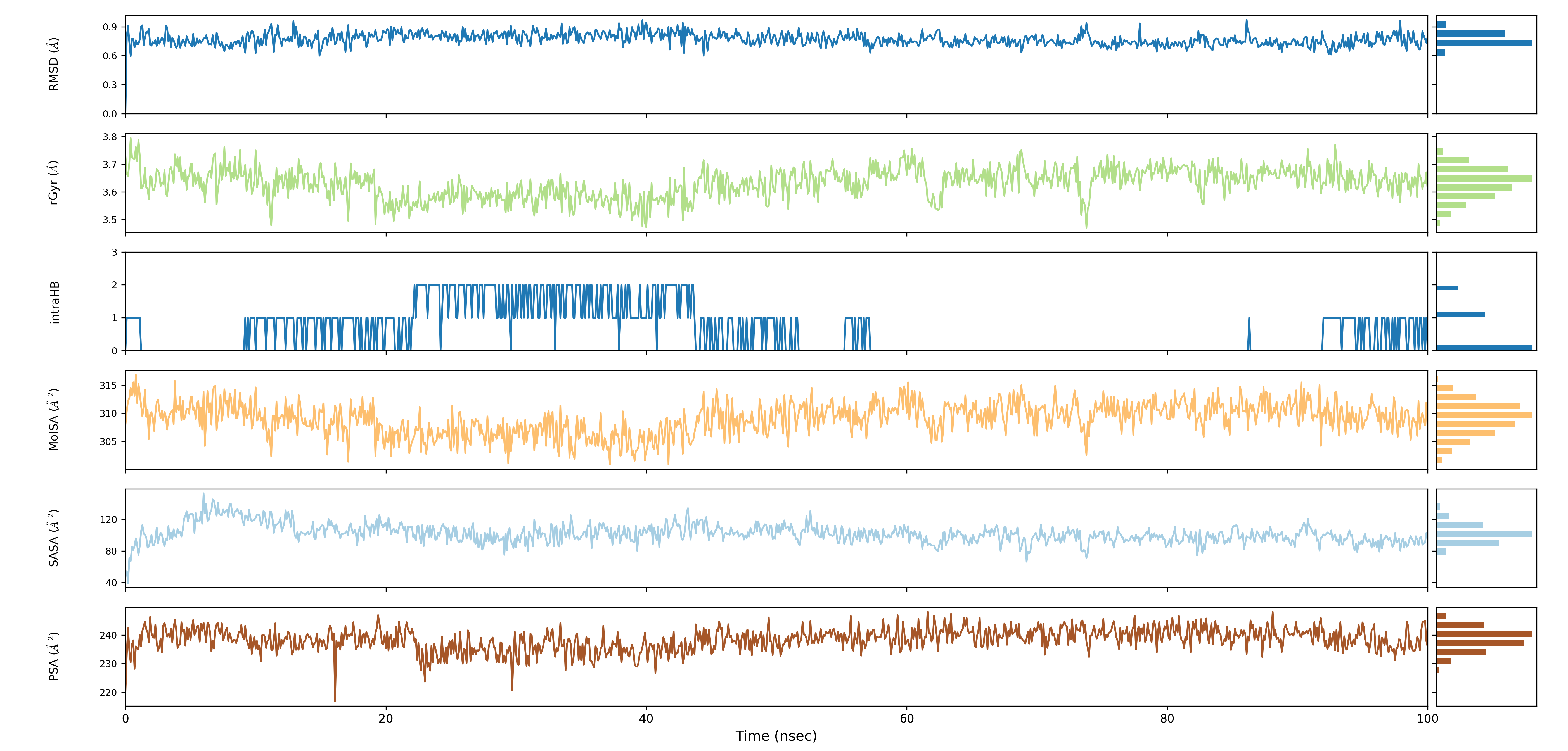

Supplement: S1 Dataset — (ZIP) [file pone.0295714.s001.zip › Data_2_6UJB/images/L-Properties.png]

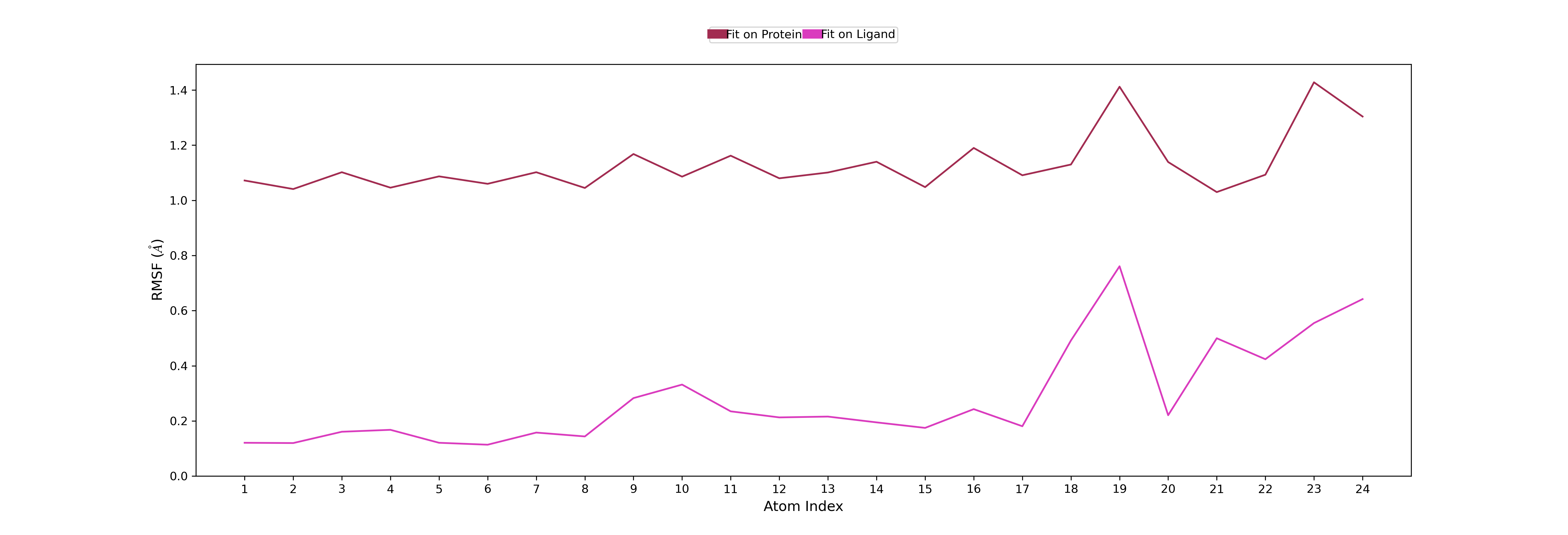

Supplement: S1 Dataset — (ZIP) [file pone.0295714.s001.zip › Data_2_6UJB/images/L-RMSF.png]

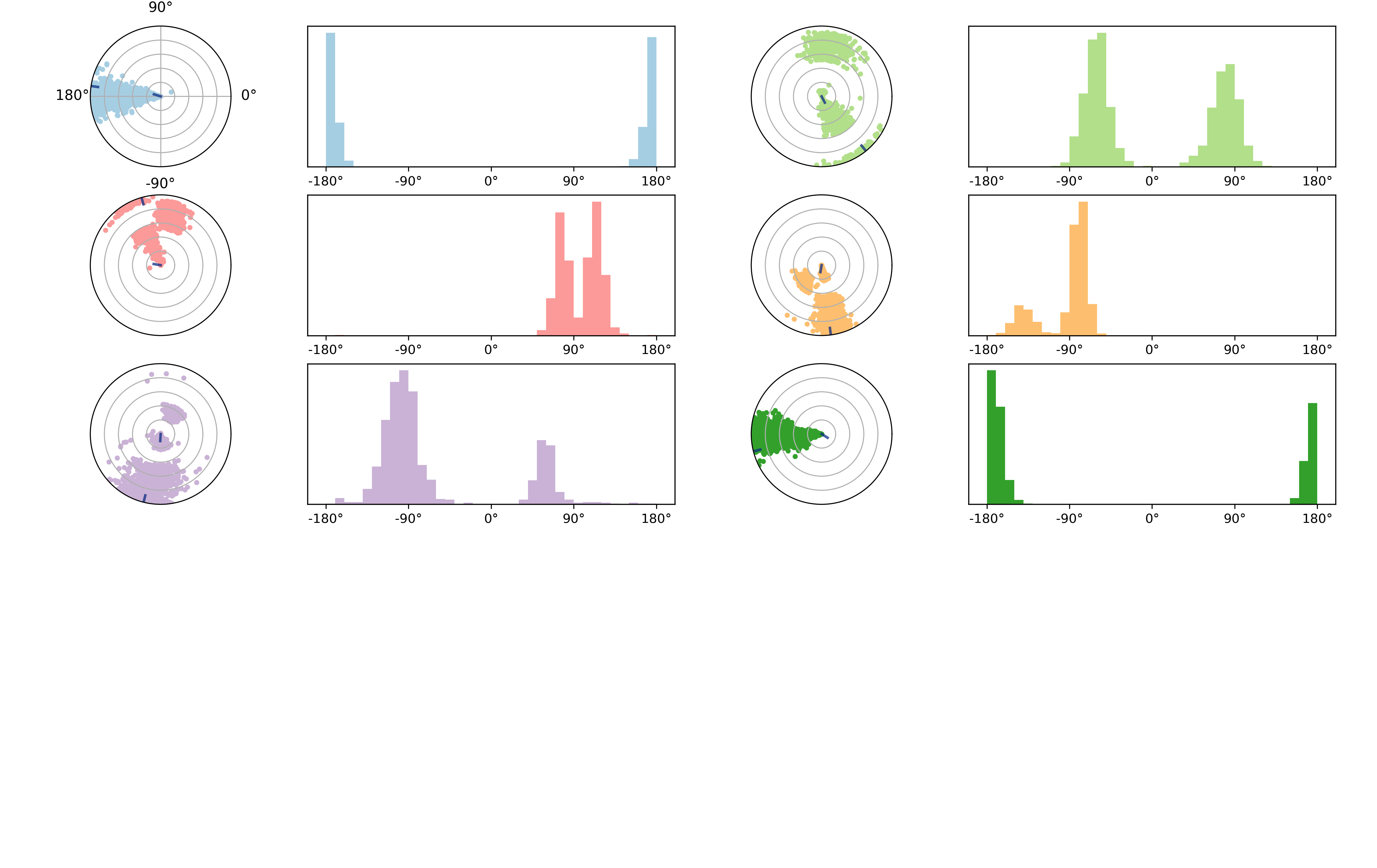

Supplement: S1 Dataset — (ZIP) [file pone.0295714.s001.zip › Data_2_6UJB/images/L-Torsions.png]

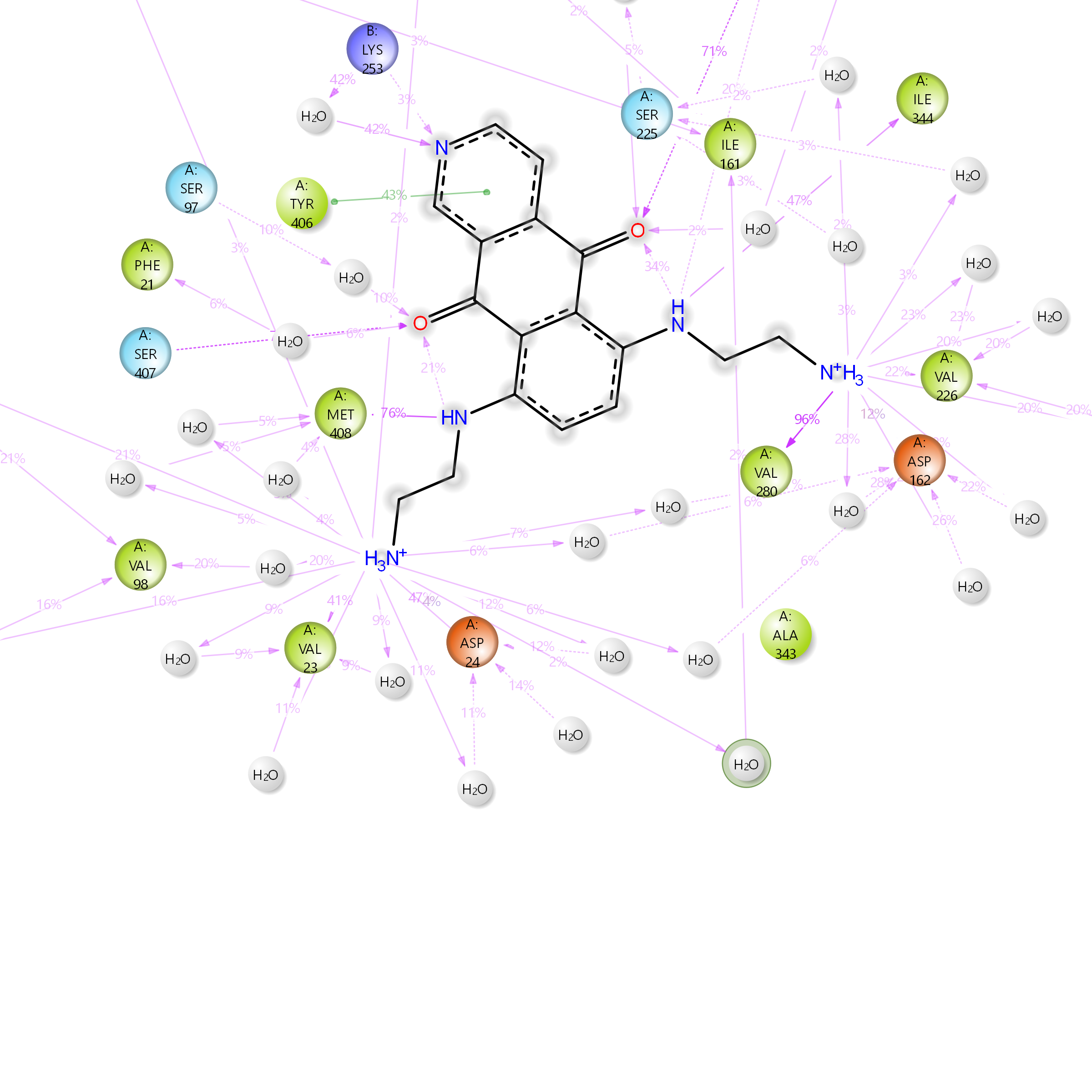

Supplement: S1 Dataset — (ZIP) [file pone.0295714.s001.zip › Data_2_6UJB/images/LP-Contacts_2d-Summary.png]

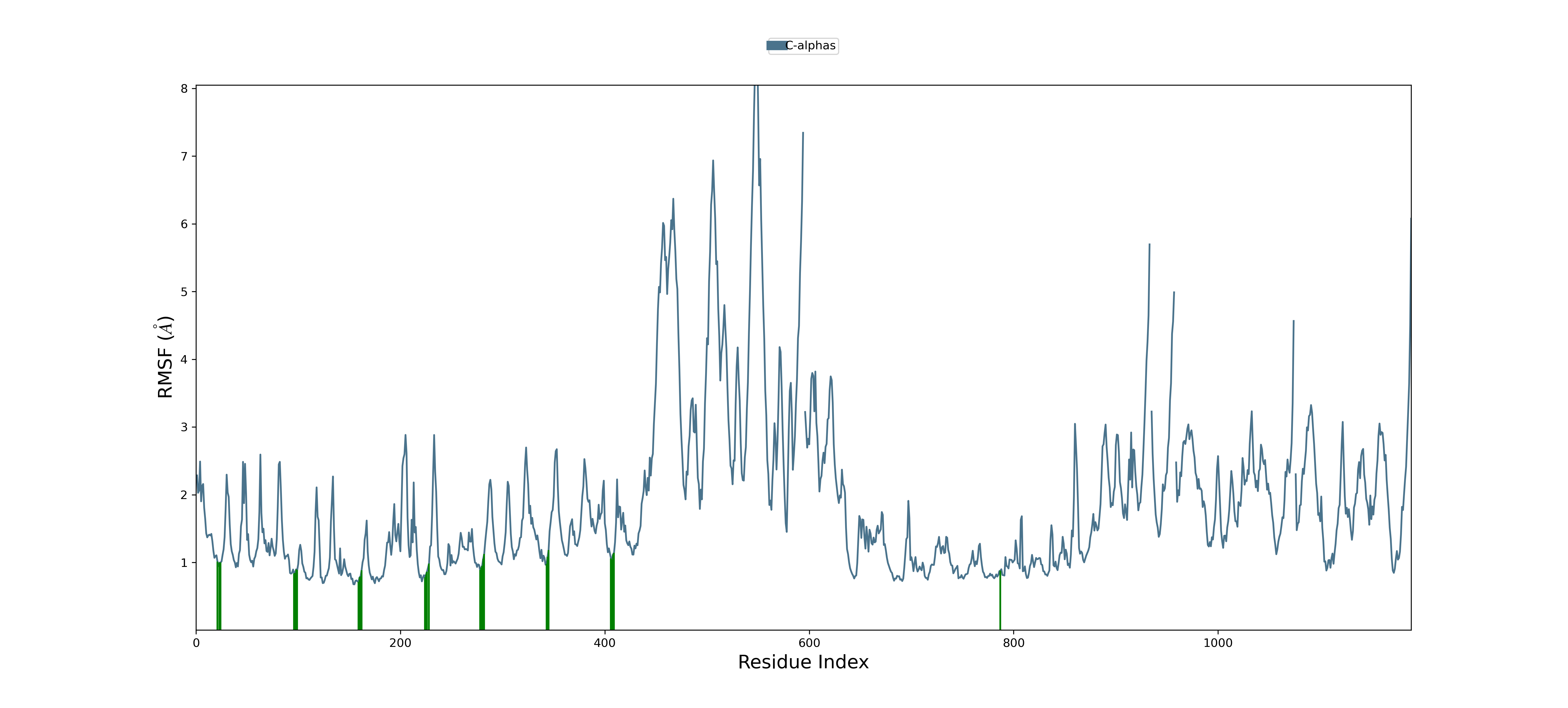

Supplement: S1 Dataset — (ZIP) [file pone.0295714.s001.zip › Data_2_6UJB/images/P-RMSF.png]

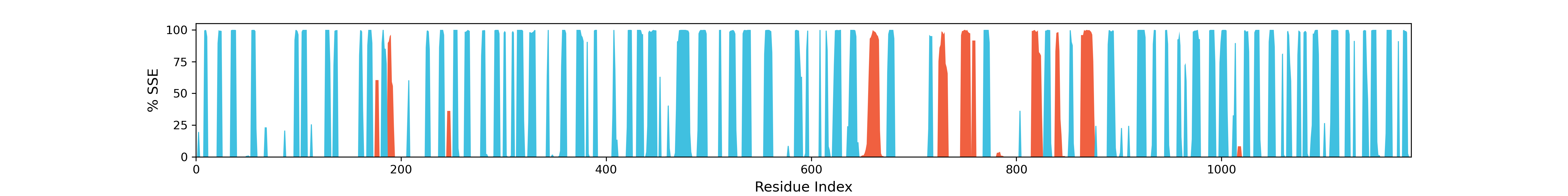

Supplement: S1 Dataset — (ZIP) [file pone.0295714.s001.zip › Data_2_6UJB/images/P-SSE_Histogram.png]

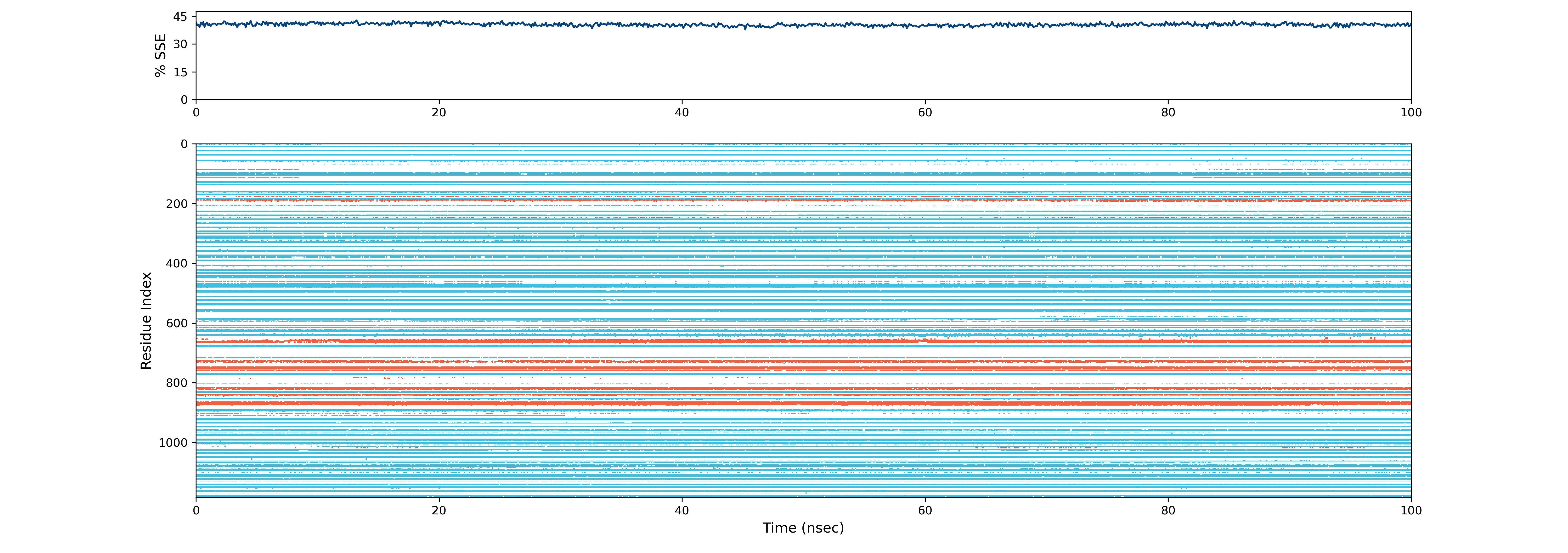

Supplement: S1 Dataset — (ZIP) [file pone.0295714.s001.zip › Data_2_6UJB/images/P-SSE_Timeline.png]

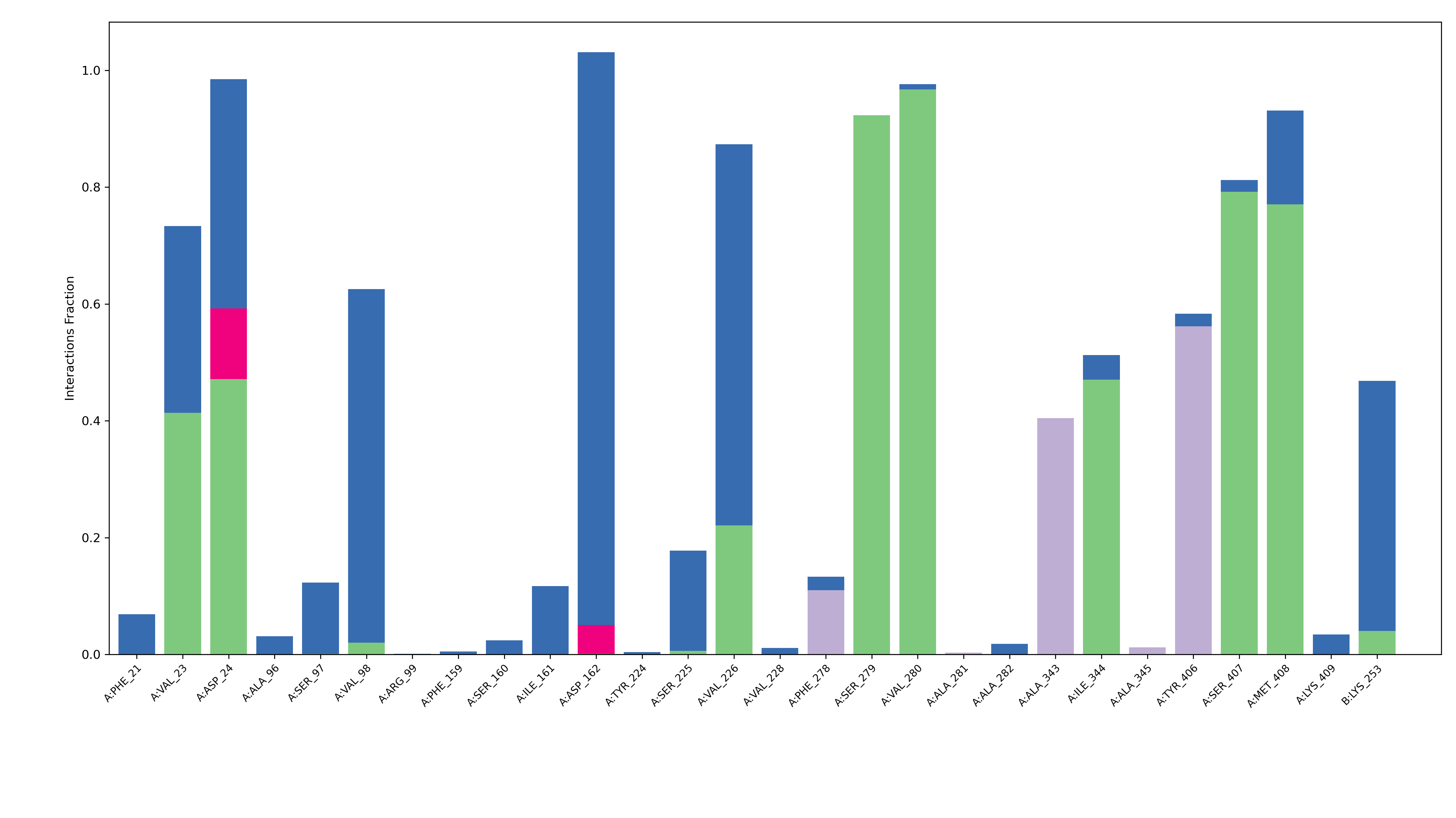

Supplement: S1 Dataset — (ZIP) [file pone.0295714.s001.zip › Data_2_6UJB/images/PL-Contacts_Histogram.png]

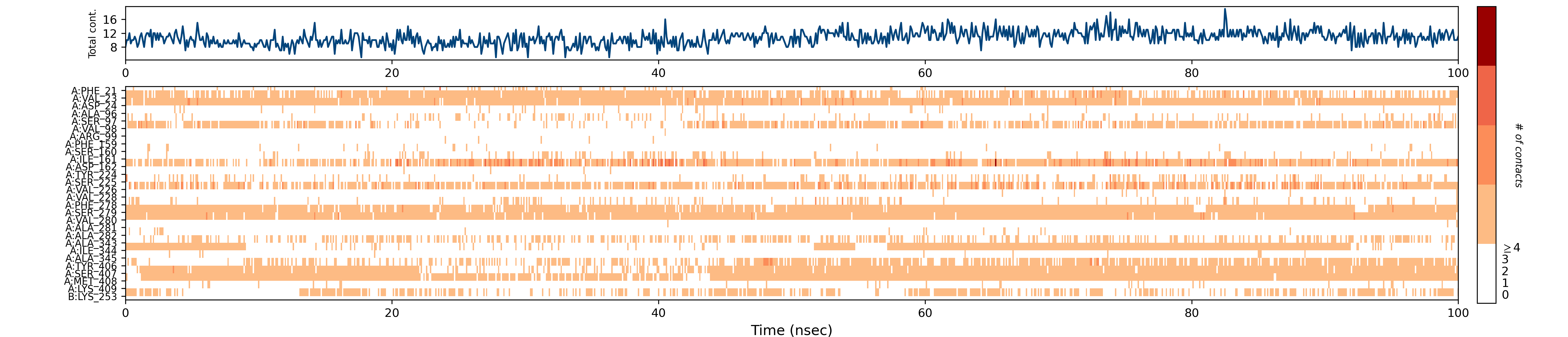

Supplement: S1 Dataset — (ZIP) [file pone.0295714.s001.zip › Data_2_6UJB/images/PL-Contacts_Timeline.png]

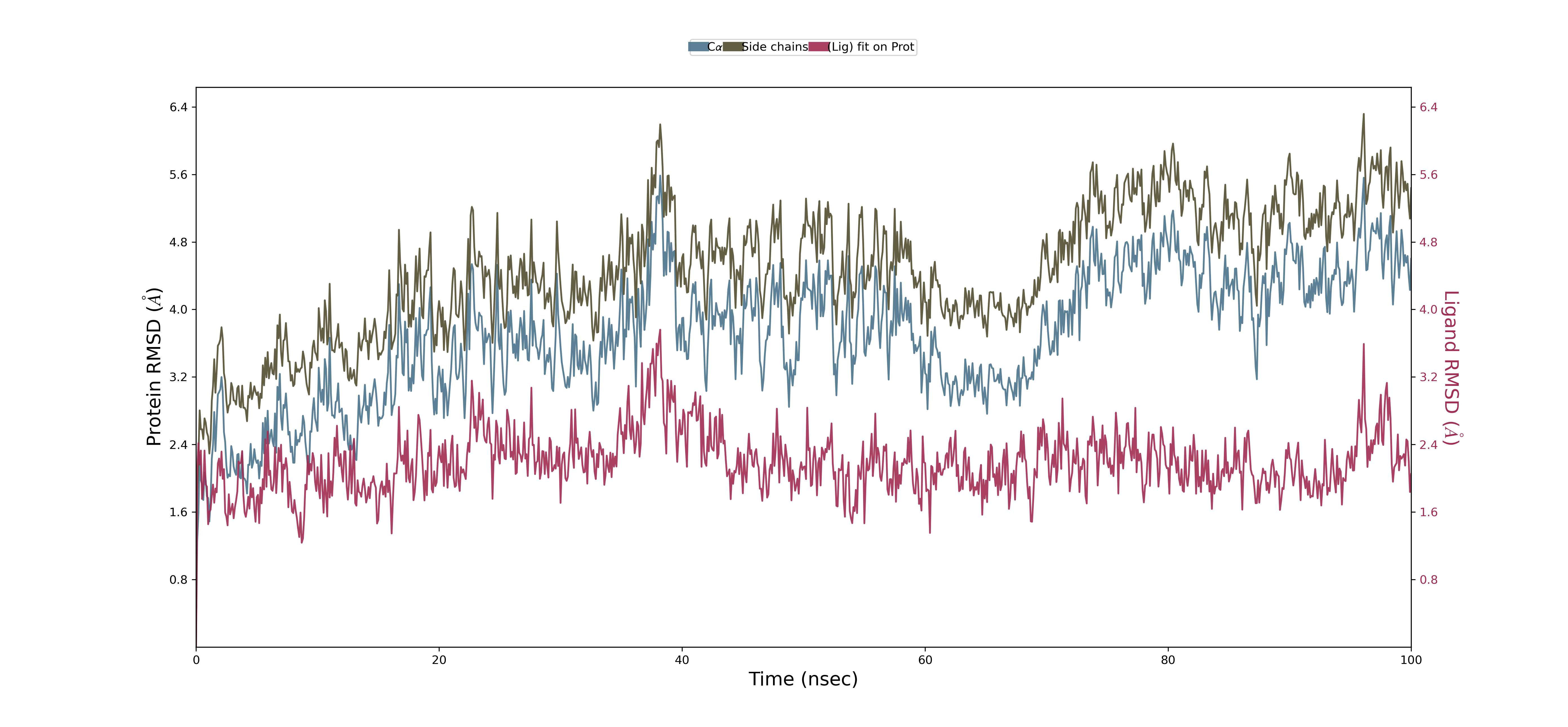

Supplement: S1 Dataset — (ZIP) [file pone.0295714.s001.zip › Data_2_6UJB/images/PL-RMSD.png]

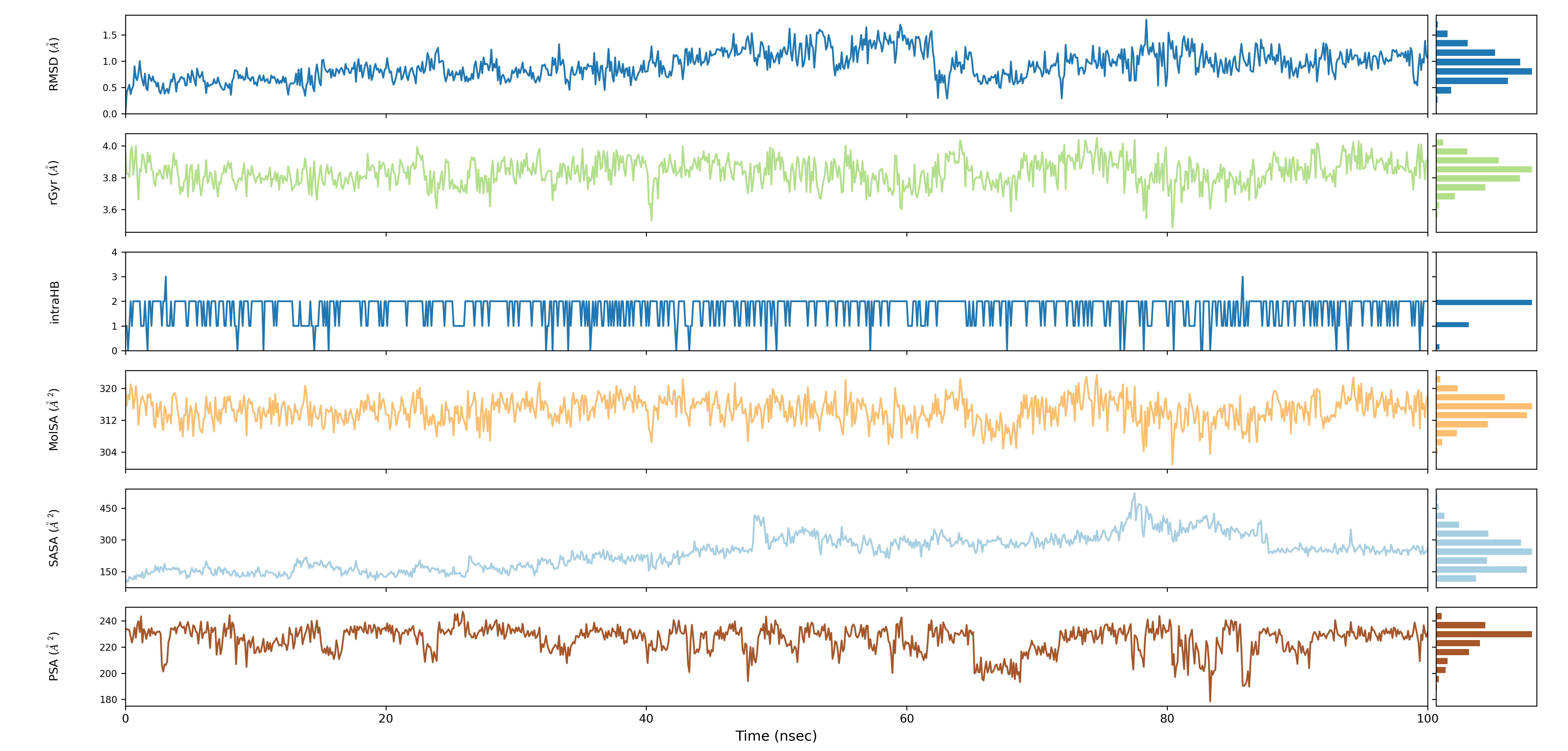

Supplement: S1 Dataset — (ZIP) [file pone.0295714.s001.zip › Data_3_6NT9/images/L-Properties.png]

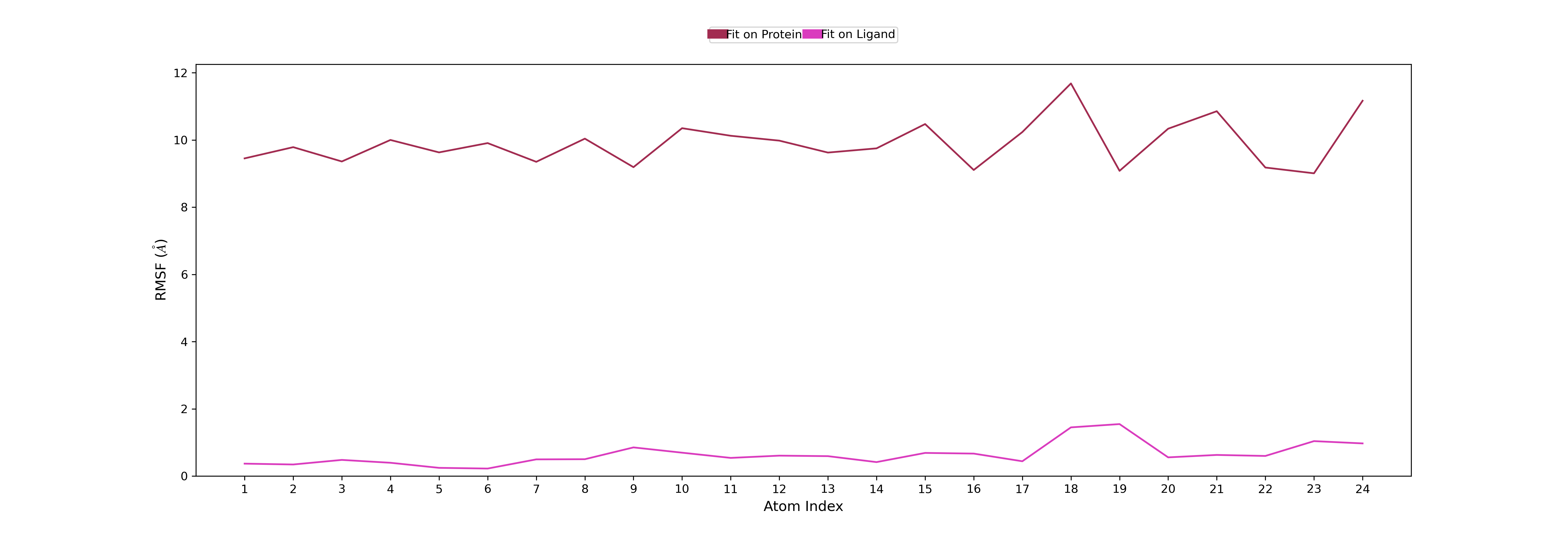

Supplement: S1 Dataset — (ZIP) [file pone.0295714.s001.zip › Data_3_6NT9/images/L-RMSF.png]

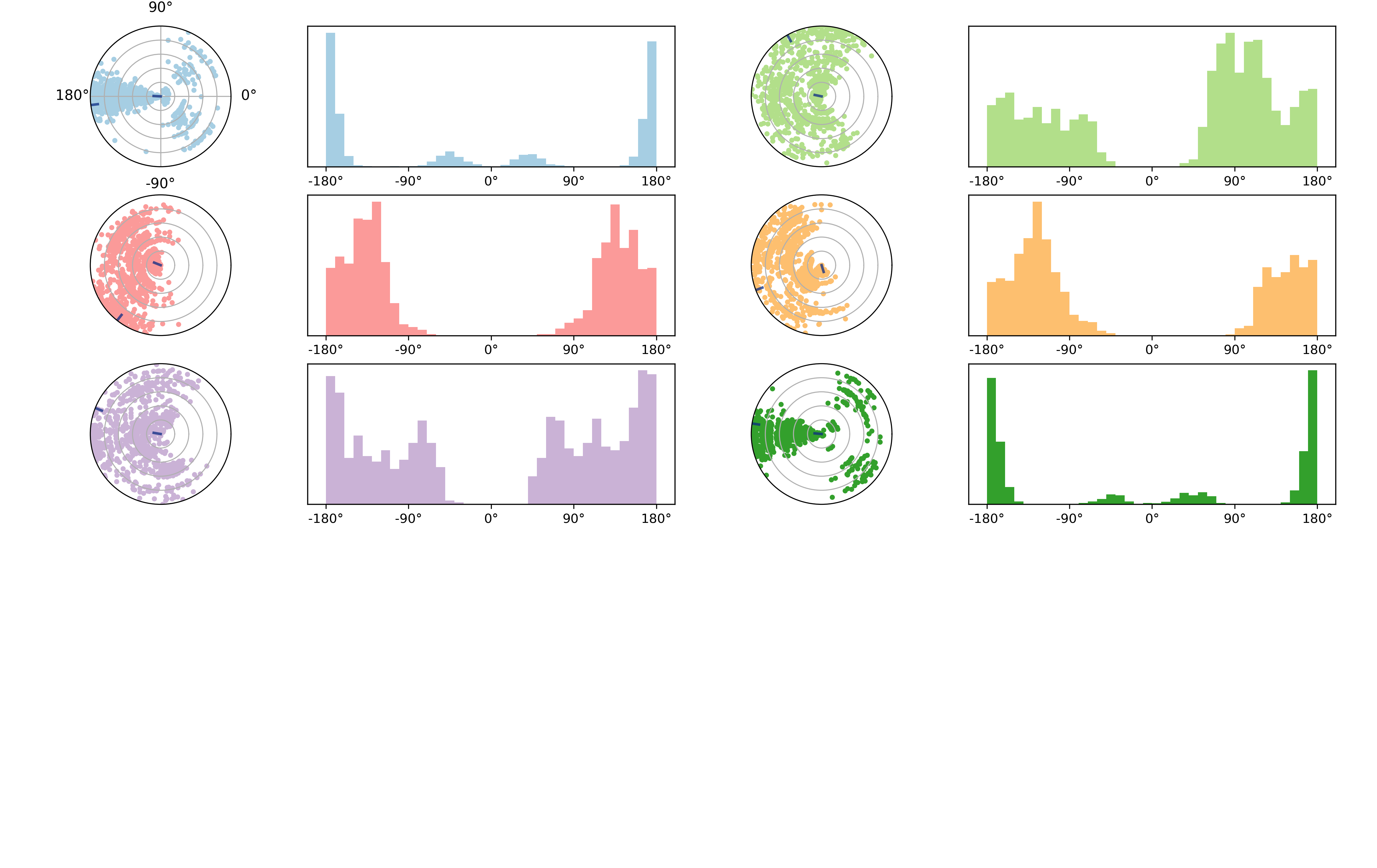

Supplement: S1 Dataset — (ZIP) [file pone.0295714.s001.zip › Data_3_6NT9/images/L-Torsions.png]

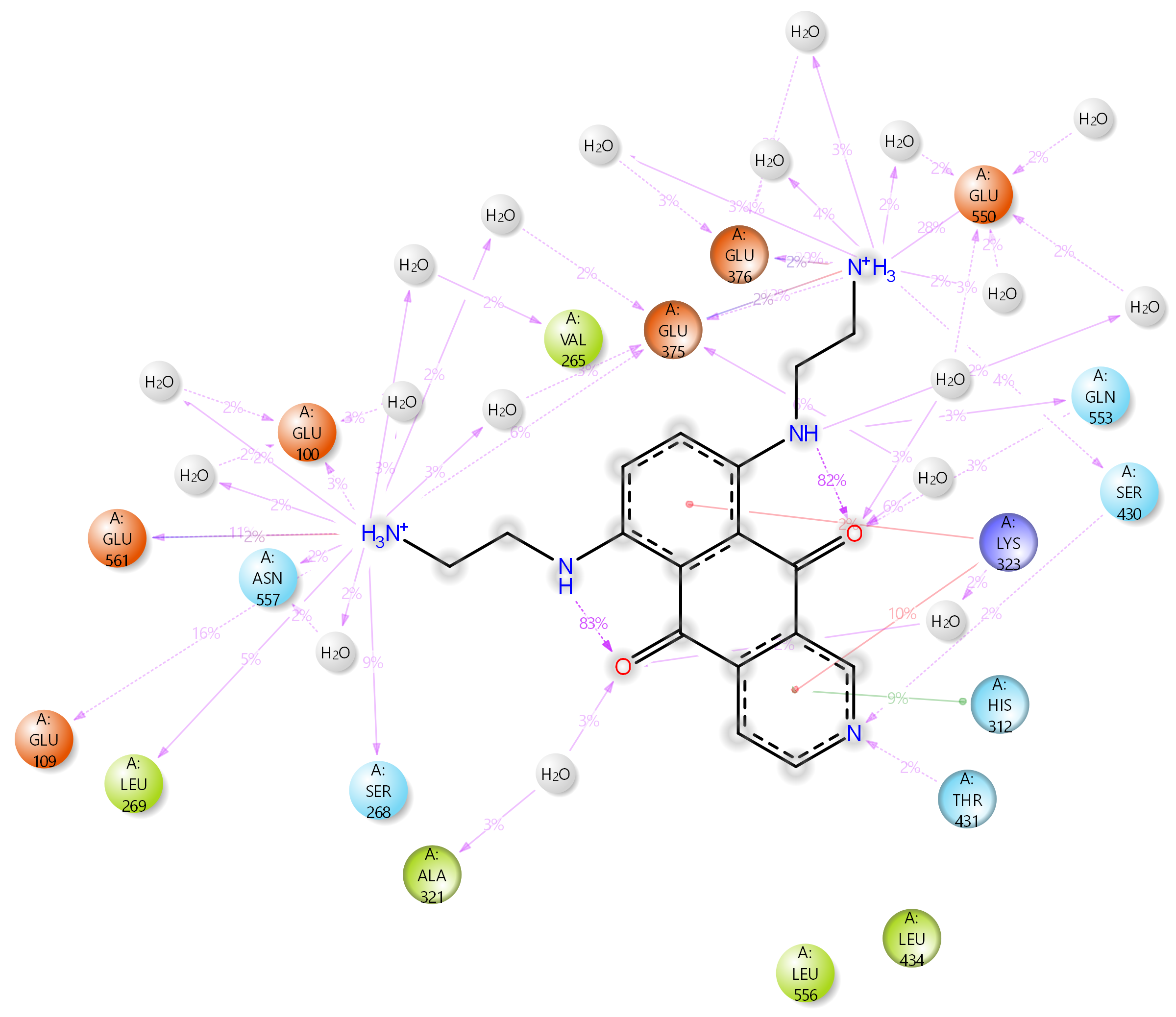

Supplement: S1 Dataset — (ZIP) [file pone.0295714.s001.zip › Data_3_6NT9/images/LP-Contacts_2d-Summary.png]

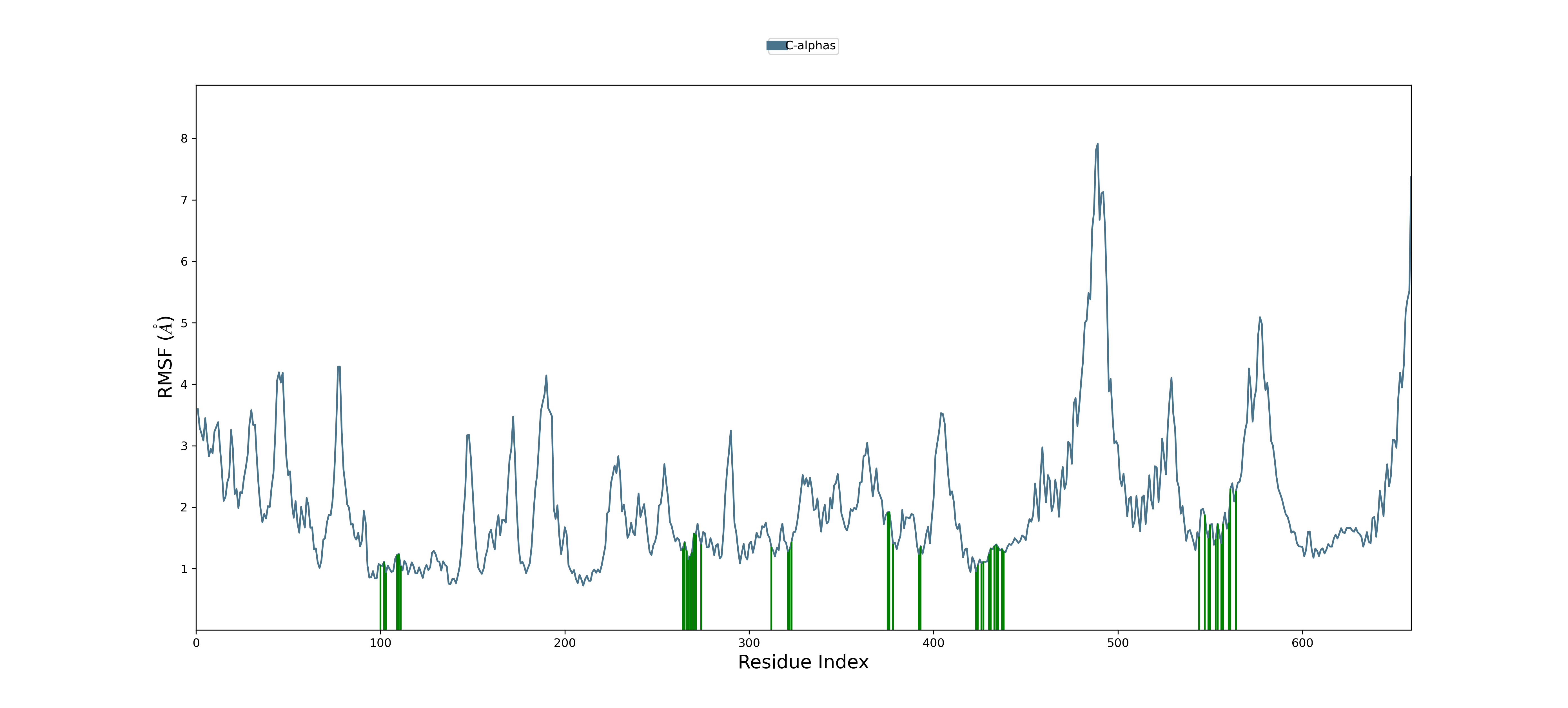

Supplement: S1 Dataset — (ZIP) [file pone.0295714.s001.zip › Data_3_6NT9/images/P-RMSF.png]

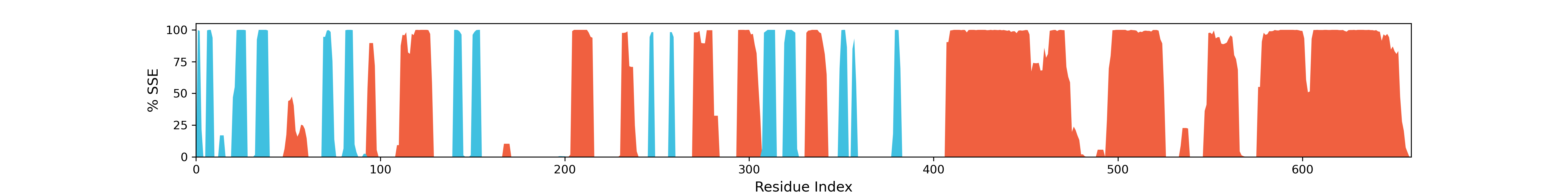

Supplement: S1 Dataset — (ZIP) [file pone.0295714.s001.zip › Data_3_6NT9/images/P-SSE_Histogram.png]

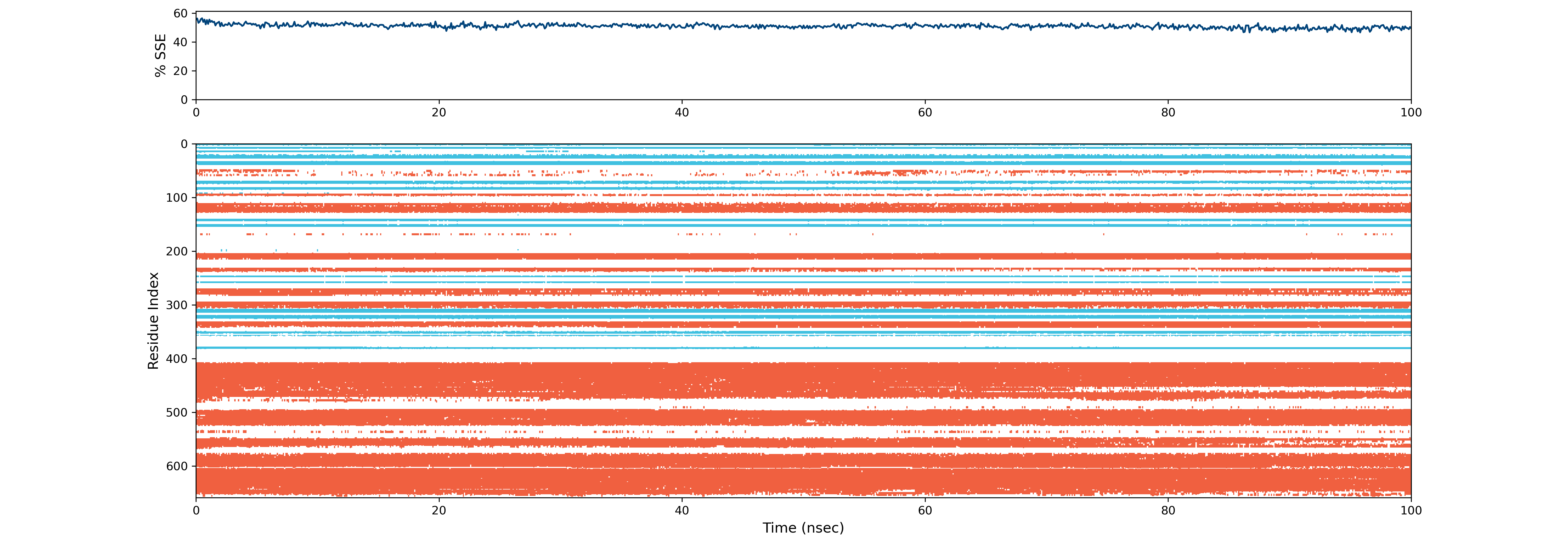

Supplement: S1 Dataset — (ZIP) [file pone.0295714.s001.zip › Data_3_6NT9/images/P-SSE_Timeline.png]

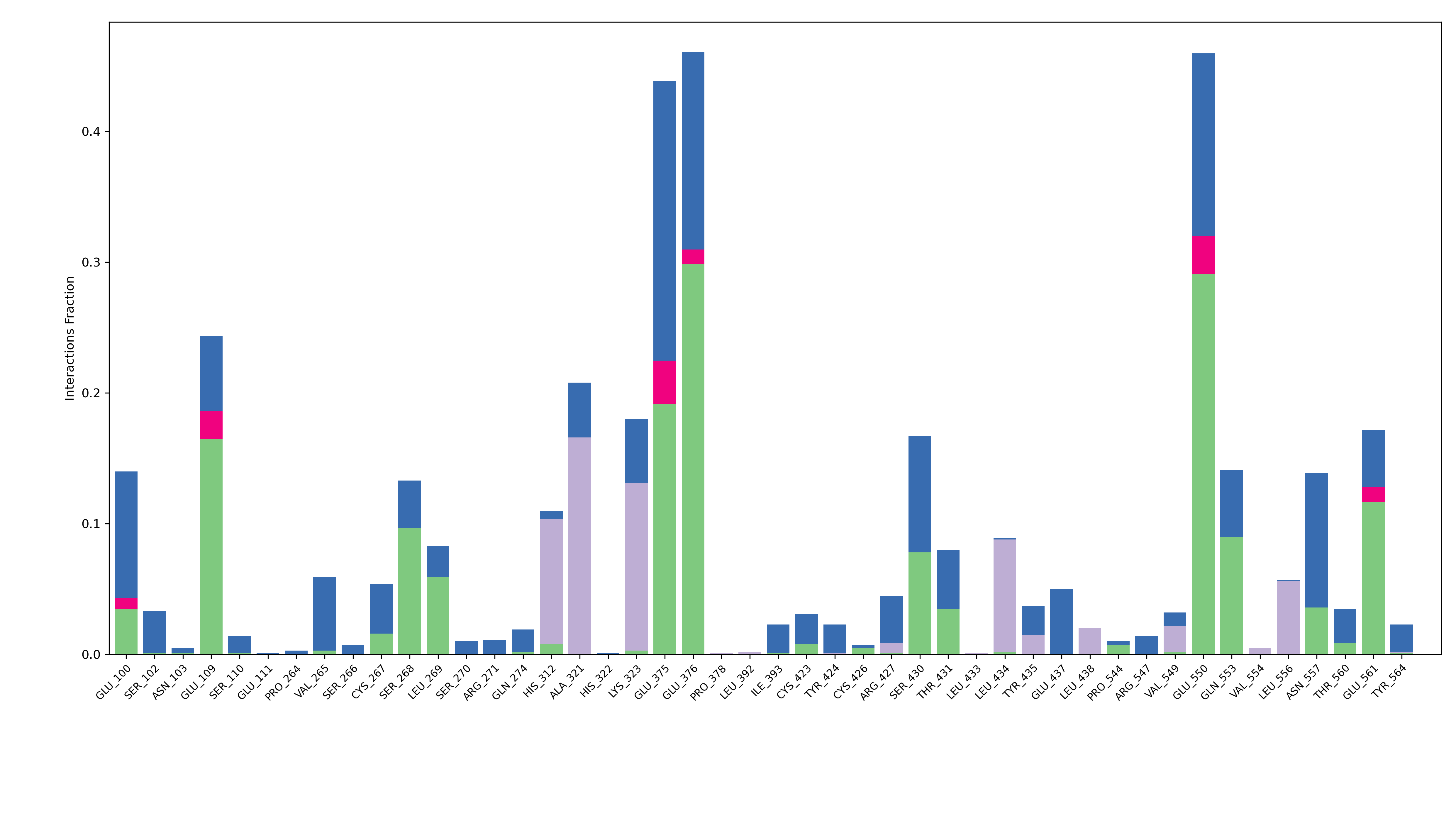

Supplement: S1 Dataset — (ZIP) [file pone.0295714.s001.zip › Data_3_6NT9/images/PL-Contacts_Histogram.png]

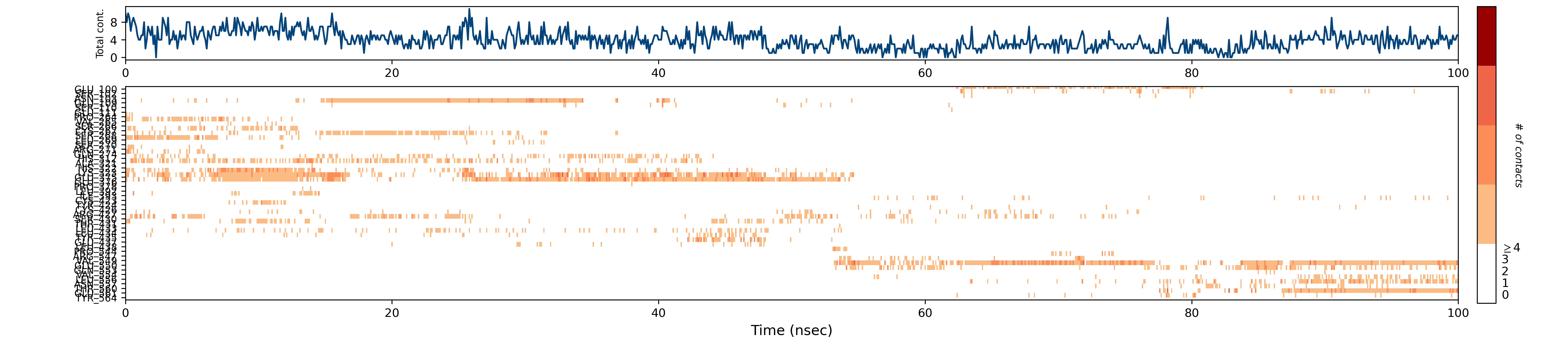

Supplement: S1 Dataset — (ZIP) [file pone.0295714.s001.zip › Data_3_6NT9/images/PL-Contacts_Timeline.png]

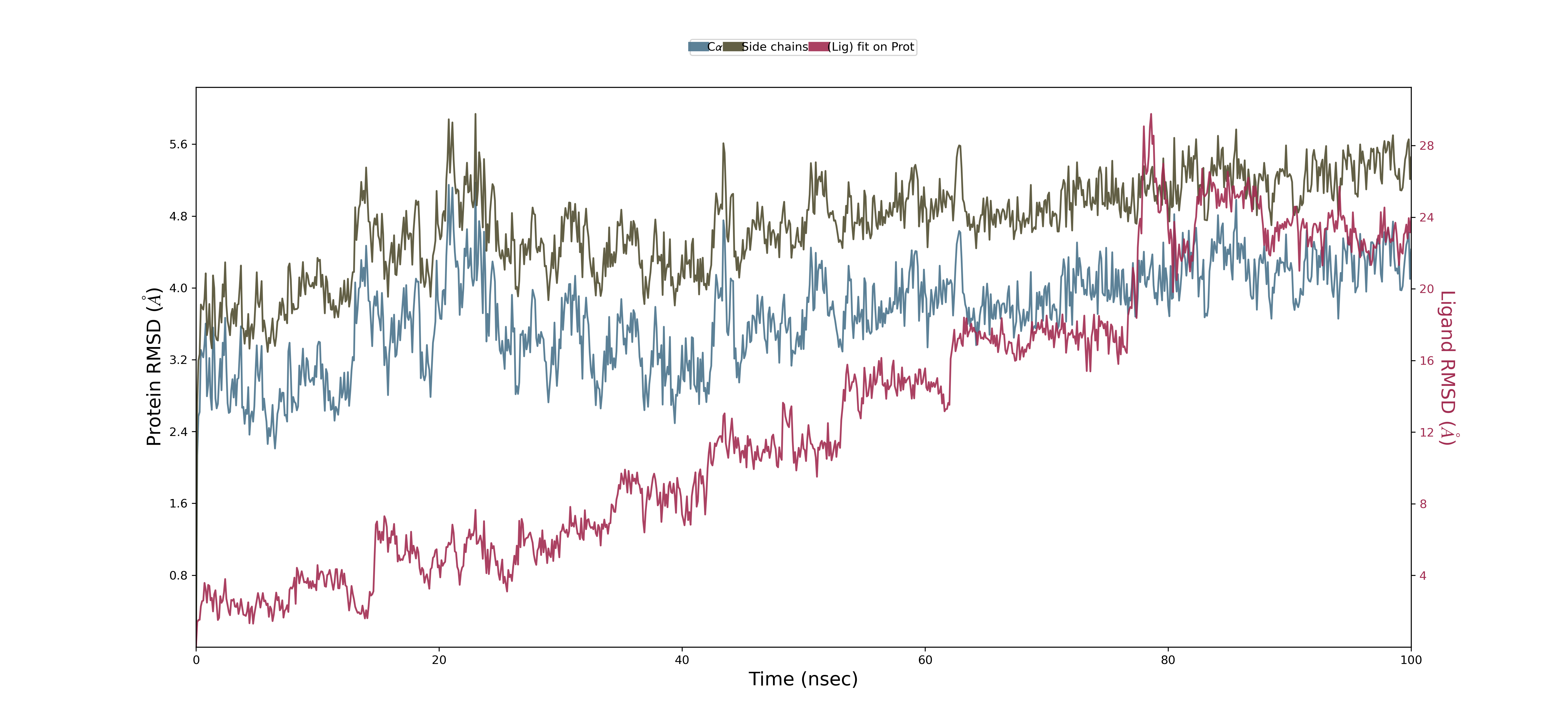

Supplement: S1 Dataset — (ZIP) [file pone.0295714.s001.zip › Data_3_6NT9/images/PL-RMSD.png]
